# Assessing the implementation of the REference FRame Alignment MEthod to compare differences in tibio-femoral kinematics during gait using five different marker sets

**DOI:** 10.3389/fbioe.2025.1530365

**Published:** 2025-04-02

**Authors:** Ariana Ortigas-Vásquez, Ann-Kathrin Einfeldt, Yasmin Haufe, Michael Utz, Eike Jakubowitz, Adrian Sauer

**Affiliations:** ^1^ Research and Development, Aesculap AG, Tuttlingen, Germany; ^2^ Department of Orthopaedic and Trauma Surgery, Musculoskeletal University Center Munich, Campus Grosshadern, Ludwig Maximilians University Munich, Munich, Germany; ^3^ Department of Orthopaedic Surgery, Laboratory for Biomechanics and Biomaterials, Hannover Medical School, Hannover, Germany

**Keywords:** kinematics, gait analysis, motion capture, marker set, joint angles, knee, tibio femoral joint

## Abstract

**Introduction:** Gait analysis plays a key role in improving our understanding of joint kinematics during locomotion, often by leveraging marker-based systems. Accessibility to marker-based systems is nevertheless limited, as they are usually associated with high equipment costs, large space requirements, and the need for lengthy data processing. These restrictions have therefore driven the need for tools that facilitate the interpretation and comparison of openly accessible kinematic datasets, even in cases where the data have been collected using distinct equipment and/or protocols.

**Methods:** This study addresses variations in kinematic data arising from the use of different marker sets, focusing specifically on the tibio-femoral joint kinematics of 15 healthy subjects during treadmill walking. By simultaneously capturing joint motion using five distinct marker sets, we were able to confirm the presence of visible differences in the raw kinematic outputs prior to data optimisation, despite their representing the same underlying motion. We subsequently implemented the REference FRame Alignment MEthod (REFRAME) to account for signal differences linked to inconsistent local reference frame orientations.

**Results and Discussion:** After REFRAME optimisation, improved convergence of the kinematic signals was observed, confirming that the differences observed in raw signals stemmed primarily from differing reference frame orientations, rather than genuine variations in joint motion. This study highlights REFRAME's potential to enhance comparability across biomechanical datasets, thus facilitating robust inter-laboratory comparisons and supporting reliable interpretations of data in clinical and research applications.

## 1 Introduction

Gait analysis involves observing, quantifying and interpreting musculoskeletal movement patterns during locomotion. A fundamental component of gait analysis is hence the assessment of joint kinematics, which focuses on the relative movement of bone segments that compose a body joint, during a specific activity. The most widely used methods of quantitative gait analysis tend to rely on optoelectronic motion capture systems. In the absence of more invasive systems leveraging, e.g., bone pins or fluoroscopic imaging, despite known limitations and the recent increased popularity of alternatives (like marker-less or inertial-measurement-unit-based systems), the gold standard has long been considered by many to be marker-based stereophotogrammetry, whereby the 3D coordinates of passive retroreflective markers (carefully positioned to indicate key anatomical landmarks) are tracked over time. Notably, quantitative gait analysis methods are often far from straightforward; they usually involve using expensive, cumbersome equipment, occupying a lot of space, following time-consuming and error-prone experimental protocols, and spending several hours attempting to reliably process and interpret complex multi-dimensional data.

Given the numerous difficulties associated with not only collecting reliable kinematic data, but also gaining access to a fully equipped gait analysis laboratory in the first place, effective data sharing and the availability of openly accessible datasets are particularly important in the field of movement biomechanics. In fact, numerous research groups have acknowledged this need and published fully documented open-access kinematic datasets in response (Bertaux et al., 2022; Lencioni et al., 2019; Moreira et al., 2021; Bahadori et al., 2021; Fakoorian et al., 2020). Furthermore, curated repositories for easy access to these and other datasets have been conveniently compiled by both individual members of the biomechanics community (Modense, 2023; Kirtley, 2023; Jung, 2023), as well as the International Society of Biomechanics (International Society of Biomechanics, 2023). The open exchange of kinematic data is undeniably valuable to the scientific community, as it enables the verification of past results, optimises the use of resources by avoiding data re-collection, and allows research to reach and be used by larger audiences (Tenopir et al., 2011). Adequate interpretation of shared data, however, importantly requires careful consideration of the extent to which different datasets are comparable.

In the context of clinical movement biomechanics, the collection and processing of gait trial data using an optical marker-based system usually involves following a detailed protocol, where that protocol encompasses a number of elements (Ferrari et al., 2008), including: 1) which marker set was used (i.e., the specific configuration of retroreflective markers on the subject’s body that were tracked), 2) how calibration trials were performed (if any), 3) what underlying biomechanical model was considered (indicating, for example, how many degrees of freedom each joint was assigned), 4) the exact definition of joint axes and local reference frames, and finally, 5) the joint rotation and translation conventions used to derive the output kinematic signals (e.g., Grood and Suntay (Grood and Suntay, 1983)). The extent to which joint kinematics stemming from the implementation of different protocols can be compared has been previously explored in multiple studies (e.g., (Ferrari et al., 2008; Kerkhoff et al., 2020; Kainz et al., 2016; Mantovani and Lamontagne, 2017; Benedetti et al., 2013) with Kerkhoff et al. and Ferrari et al. having compared six and five different marker sets, respectively). Notably, if we consider that minor adaptations (e.g., placing the same marker on the skin vs. on a wand) are enough to make an existing marker set “different” from the original, we could hypothetically implement “different” marker sets while keeping most (if not all) other elements of our gait analysis protocol (i.e., calibration trials, biomechanical model and joint axes definitions) fundamentally the same. Nevertheless, the use of different marker sets is usually inherently also associated with differences in other protocol components, such as underlying biomechanical model and/or local reference frame definitions. Both Kerkhoff et al. (Kerkhoff et al., 2020) and Ferrari et al. (Ferrari et al., 2008) clearly reported that the marker set (or “protocol” in Ferrari et al.) undeniably influenced gait analysis results, at the very least in terms of the quantification of out-of-sagittal-plane rotations of the knee joint. These studies reported some marker sets led to “contradictory” or “opposite” tibio-femoral ab/adduction patterns, as well as differences in waveform trends and ranges of motion in ab/adduction and int/external rotation. In both publications, the authors suggest that rather than being caused by differences in the anatomical landmarks represented by individual markers *per se*, these inconsistencies are likely primarily the result of differences in the underlying joint model definitions associated with the different marker sets. This conclusion is strongly supported by the findings of our recent work, which clearly demonstrates the considerable effect that even minor variations in the position and orientation of local coordinate systems can have on the associated kinematic signals (Postolka et al., 2022; Ortigas-Vásquez et al., 2023; Ortigas-Vásquez et al., 2025). Reliance on a different biomechanical model inherently implies the exact definition of each local segment reference frame is probably likewise different, thus feasibly leading to distinct representations of what may be the same underlying joint motion pattern. Although the International Society of Biomechanics had previously aimed to address part of these potential sources of variation by referencing the work of Grood and Suntay (Grood and Suntay, 1983) as their recommended approach for describing tibio-femoral kinematics (Wu et al., 2002), the method endorsed a clear rotation sequence convention but still allowed for variation in the exact orientation of the local segment’s axes. Consequently, even methods that adhere to Grood and Suntay’s recommended rotation sequence may still not be able to produce consistent frame orientations (Ortigas-Vásquez et al., 2025).

For a single joint movement sequence, we can reasonably expect the use of different marker sets to capture and estimate kinematic signals from that motion to lead to visually different results, even though the underlying motion pattern is indeed one and the same (Ferrari et al., 2008; Kerkhoff et al., 2020). This poses a principal challenge to efforts favouring the open exchange of data in the biomechanics field, as kinematic datasets stemming from different sources are likely to be associated with different marker sets and motion capture protocols, especially considering that resources, staff experience, and equipment preferences vary between laboratories. There is therefore a pressing need for an approach that enables a common repeatable representation of joint movement patterns, regardless of the underlying experimental protocol and equipment used to capture them. The REference FRame Alignment MEthod (REFRAME) was recently demonstrated to achieve this objective for three sets of tibio-femoral joint kinematics that had all been collected using the same data capture protocol, but three distinct axis definitions (Ortigas-Vásquez et al., 2025; Ortigas-Vásquez et al., 2024).

In the current study, we now aim to explore REFRAME’s ability to account for possible differences in the local segment reference frame orientations associated with the use of different marker sets. Expected to lead to signal convergence for common underlying joint motion patterns, even if obtained following different protocols, the implementation of REFRAME in this study will provide new insight into how we can better compare joint motions associated with different experimental setups. Therefore, in this study, we compare the rotational tibio-femoral kinematic signals of 15 healthy subjects during treadmill walking using five distinct marker sets, both before and after REFRAME implementation. The exact origin position and axis orientations of the local segment reference frames depended on the chosen marker set, each marker’s exact placement, and the calculation methods associated with the corresponding underlying model. This study focuses on the relative orientations of the segment frames between the different marker sets and their subsequent implications on kinematic signals and tibio-femoral joint angles during treadmill gait.

## 2 Materials and methods

### 2.1 Marker models

**FIGURE 1:**
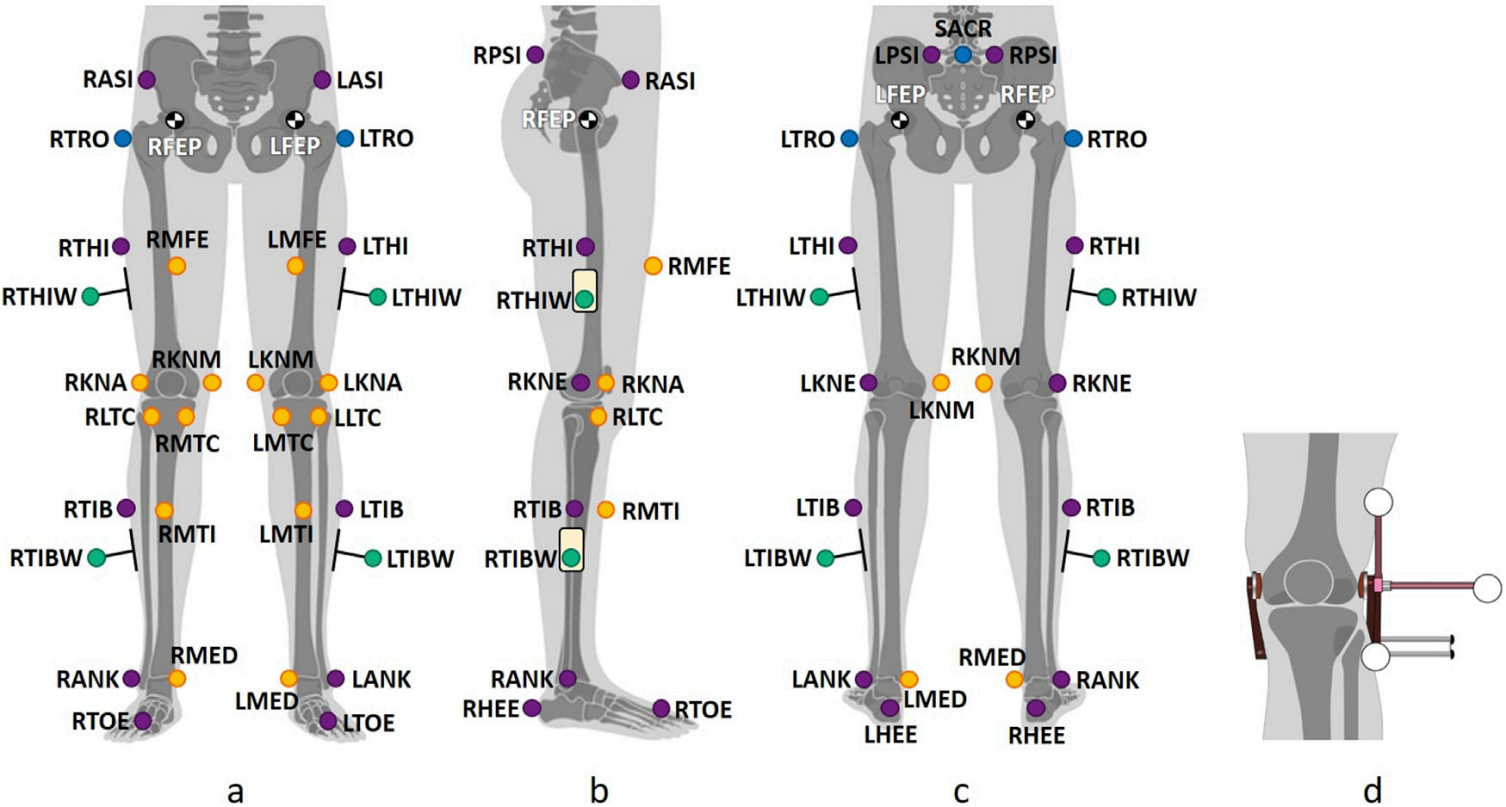
Marker placement in frontal **(a)**, sagittal right **(b)**, and dorsal **(c)** views. The basic *PiG* model (Davis et al., 1991) uses 16 markers (purple), which are either supplemented or replaced by specific markers (cmp. Table 1) to generate *PiG wand* (green), *MA* (Stief et al., 2013) (blue), and *MiKneeSoTA* (Einfeldt et al., 2024) (yellow). LFEP and RFEP represent the hip joint centers, determined using anthropometric regression equations (Davis et al., 1991), scaled based on manually measured distances (e.g., leg length) for most marker sets, or on marker positions (e.g., LTRO and RTRO on the greater trochanters) for *MA*. The positioning of the Knee Alignment Device during the static trial for *KAD* is shown in **(d)**. Adapted with permission from Figure 1 in (Einfeldt et al., 2024).

Five different marker sets (Figure 1; Table 1) were simultaneously used to capture the joint kinematics of both knees on each of 15 asymptomatic subjects (age: 27 ± 7 years, body mass: 70.6 ± 12.6 kg, BMI: 22.6 ± 3.1 kg/m^2^) during treadmill walking. All subjects provided their informed consent, and the study protocol and all methods used in this study complied with the principles of the Declaration of Helsinki. The study was approved by the ethics committee of the Hannover Medical School (ethics vote no.: 10,861, approval date: 12-04-2023).

**TABLE 1:** List of markers shown in Figure 1: Markers 1–8 (blue) are used for the basic *PiG* model (Davis et al., 1991). In *PiG wand*, markers 3 and 5 (purple) are replaced by markers 9 and 10 (green). The *MA* model (Stief et al., 2013) adds marker 11 (blue) to *PiG*, replaces left and right markers 2 (purple) with markers 12 (blue), and uses markers 15 and 19 during the static trial. The *MiKneeSoTA* model (Einfeldt et al., 2024) includes all markers except 3, 5, 11, and 12.

No.	Abbr	Definition	Landmark
1	LASI, RASI	Left, Right Anterior Superior Iliac	
2	LPSI, RPSI	Left, Right Posterior Superior Iliac	
3	LTHI, RTHI	Left, Right Thigh	lower lateral thigh
4	LKNE, RKNE	Left, Right Knee	lateral flexion-extension axis of the knee
5	LTIB, RTIB	Left, Right Tibia	lower lateral shank
6	LANK, RANK	Left, Right Ankle	lateral malleolus, transmalleolar axis
7	LHEE, RHEE	Left, Right Heel	posterior calcaneous, same height as LTOE, RTOE
8	LTOE, RTOE	Left, Right Toe	2nd metatarsal head
9	LTHIW, RTHIW	Left, Right Thigh (Wand)	lower lateral thigh
10	LTIBW, RTIBW	Left, Right Tibia (Wand)	lower lateral shank
11	SACR	Mid Sacrum	midpoint between left and right posterior superior iliac
12	LTRO, RTRO	Left, Right Greater Trochanter	prominence of the greater trochanter
13	LMFE, RMFE	Left, Right Mid Femur	mid-thigh, where the quadriceps belly crosses the femur
14	LMTI, RMTI	Left, Right Mid Tibia	medial surface of the tibia
15	LKNM, RKNM	Left, Right Knee Medial	medial flexion-extension axis of the knee
16	LLTC, RLTC	Left, Right Lateral Tibial Condylus	anterio-lateral tibial condyle
17	LMTC, RMTC	Left, Right Medial Tibial Condylus	anterio-medial tibial condyle
18	LKNA, RKNA	Left, Right Knee Anterior	lateral epicondyle anterior to LKNE, RKNE
19	LMED, RMED	Left, Right Medial Ankle	medial malleolus, transmalleolar axis

Three of the marker sets used were variations of the standard Helen Heyes Model, also known as Plug-in-Gait (PiG) (Davis et al., 1991; Vicon, 2021). The first marker set, *“PiG,”* was the standard PiG model consisting of 16 markers (Figure 1; Table 1; in purple) – four waist markers (LASI, RASI, LPSI, RPSI), two thigh markers (LTHI, RTHI), two knee markers (LKNE, RKNE), two shank markers (LTIB, RTIB), two ankle markers (LANK, RANK) and four foot markers (LHEE, RHEE, LTOE, and RTOE). The second marker set, *“PiG wand”,* replaced skin markers on the thigh and shank with wand markers (in green; Figure 1; Table 1). The third variation of the standard PiG marker set, “*KAD,”* consisted of the PiG model with the Knee Alignment Device (KAD, Figure 1) (Motion Lab Systems, Inc., Baton Rouge LA, USA). The KAD was only used in the static trial to define the knee axis, while for the dynamic trials the KAD was removed and the same markers as in *PiG wand* were used.

The fourth marker set, *“MA,”* consisted of 29 markers (Stief et al., 2013) (Figure 1; Table 1; in blue). Six of them were mounted on the upper body and are therefore not relevant for this study. On the lower body, only three markers were used to define the pelvis (LASI, RASI, SAC). For the thigh and shank segments, in addition to the markers of the standard *PiG* marker set, two extra markers were placed laterally on the greater trochanters (LTRO, RTRO) and frontally on the shins (LMTI, RMTI). During the static trial, additional medial markers were also placed along the extension of the respective joint axis on the medial side of the knee (LKNM, RKNM) and the ankle (LMED, RMED). The thigh and shank markers were wand markers. The difference between this marker set and *PiG wand* was: 1) the calculation of the hip joint center (which in the case of *MA* considered the position of the greater trochanter markers, LTRO and RTRO), and 2) the use of the medial knee markers to define the knee flexion axis (Stief et al., 2013).

The fifth marker set was the recently published *MiKneeSoTA* (Einfeldt et al., 2024), consisting of 30 markers (Figure 1; Table 1; in yellow). In addition to the 16 markers of *PiG wand*, seven additional markers were placed on each leg: one on each thigh (LMFE, RMFE), one on each shank (LMTI, RMTI), four around each knee joint (LKNA, LKNM, LLTC, LMTC, RKNA, RKNM, RLTC, RMTC), and one on each medial malleolus at the ankles (RMED, LMED). The markers of all five marker sets were placed for every trial, but only the markers specific to each marker set were used to calculate the corresponding kinematics.

### 2.2 Measurement protocol

Prior to the execution of the dynamic trials, a static trial was captured. Nine consecutive steps were recorded with a standardised gait speed of 4 km/h after 2 minutes of familiarisation walking on the treadmill. The marker trajectories were captured using an optical motion capturing system with 12 cameras (200Hz, six M3 and six M5 Miqus cams, Qualisys AB, Göteborg, Sweden). Data pre-processing was performed using the corresponding Qualisys Track Manager (QTM, Vers. 2023.2), while event detection and joint angle calculations for the PiG-based approaches (*PiG*, *KAD* and *PiG wand*) as well as *MA* were executed using Nexus (Vers. 1.8.5, Vicon Motion System Ltd., Oxford, UK). For the MiKneeSoTA model, the events and the marker trajectories of the static and the dynamic trial were exported and postprocessed using C++. Post-processing included a frame-by-frame optimisation approach to adjust best-fit cylinders that have been generated based on the relative position of the lower limb markers during the static trial (Einfeldt et al., 2024).

### 2.3 Kinematic definitions

To determine tibio-femoral joint angles from the motion capture data, the 3D coordinates of specific markers according to each marker protocol (for further details see (Davis et al., 1991; Vicon, 2021) for PiG, PiG wand and KAD, (Stief et al., 2013) for MA, and (Einfeldt et al., 2024) for MiKneeSoTA) were used to determine three orthogonal axes (one mediolateral, one anteroposterior, and one proximodistal) in order to define local reference frames for the femur and tibia at every timepoint. Kinematic signals were then determined based on the relative poses of these local reference frames.

For left knees, the mediolateral (x-axis) coordinates of markers were first inverted (i.e., multiplied by −1) so that all knees could be treated as right knees during data processing. The underlying description of rotational tibio-femoral joint kinematics was consistent for all five marker sets. Joint rotations were given for the tibial segment relative to the femoral segment, following an intrinsic XYZ Cardan sequence of positive rotations around a right-handed coordinate system equivalent to 1) knee extension – 2) tibial adduction – 3) internal tibial rotation, where the x-axis pointed laterally, the y-axis pointed anteriorly, and the z-axis pointed proximally. In figures, flexion angles were purposefully illustrated as positive (despite representing a negative rotation around the laterally pointed x-axis) to allow for easier comparison against figures in previous studies.

### 2.4 Data postprocessing using REFRAME

After five “raw” sets of kinematic signals characterising all three joint angles based on each of the five marker sets had been determined, REFRAME was then implemented. The approach highlights the importance of aiming for local segment reference frames that are consistent in orientation (as well as position, in cases where joint translations are also being analysed) when comparing kinematics signals. REFRAME acknowledges that two sets of kinematic signals could be representing the same underlying motion pattern, despite consisting of visually different curves (in shape and/or magnitude; for further details see (Ortigas-Vásquez et al., 2025; Ortigas-Vásquez et al., 2024)). Consistent local reference frame alignments are targeted by, in this case, allowing the femoral and tibial reference frames to be re-oriented relative to the segments they represent, in order to thus minimise a user-specified cost function. Here, the orientations of the tibial and femoral reference frames were optimised to find constant transformations to minimise the root-mean-square (RMS) of int/external rotations and ab/adduction (both with criteria weighting of 1) (Ortigas-Vásquez et al., 2023; Ortigas-Vásquez et al., 2025). To maintain clinical interpretability, any non-zero values for the root-mean-square error (RMSE) of the flexion angle relative to that of the reference dataset (PiG) were penalised using a weighting of 0.2, and transformations of the femoral frame around the flexion axis were restricted (Ortigas-Vásquez et al., 2024).

To quantify the differences between the kinematic signals of the distinct marker sets before and after REFRAME, RMSE values between the curves were calculated for each subject and trial. The RMS (i.e., RMSE vs. constant 0) values of ab/adduction and int/external rotation signals were additionally calculated to assess cost function parameters before vs. after REFRAME implementation. Averages were calculated across all nine trials for each of the 30 knees (2 knees * 15 subjects), as well as across all trials and subjects. The transformations of the tibial and femoral frames implemented as part of REFRAME optimisation were also reported. These angles were also expressed as an intrinsic Cardan sequence around mediolateral, anteroposterior, and longitudinal segment axes (in that order), where positive rotations were determined using the right-hand rule. Positive axis directions pointed laterally, anteriorly and proximally. All further analyses of the marker-based kinematic data (including the calculation of averages and standard deviations, plotting of time-series kinematic signals, and calculation of RMS and RMSE values) were executed using MATLAB (vR2022a; The Mathworks Inc., Natick, Massachusetts, United States).

## 3 Results

**FIGURE 2:**
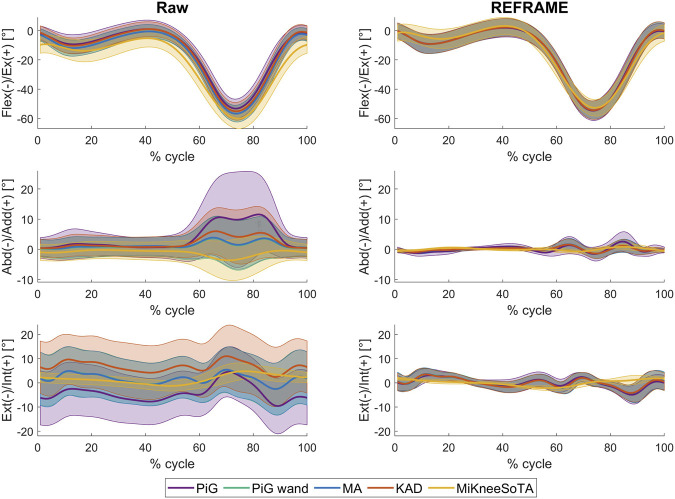
Mean ± standard deviation of tibio-femoral joint angles (in degrees), calculated across all knees and trials, for each of the five marker sets: *PiG* (purple), *PiG wand* (green), *MA* (blue), *KAD* (orange), and *MiKneeSoTA* (yellow), both before (left; “Raw”) and after (right; “REFRAME”) REFRAME implementation.

Prior to REFRAME implementation, mean rotational tibio-femoral kinematic signals and the corresponding standard deviations calculated across all knees and trials revealed visible differences between marker sets for ab/adduction and int/external rotation especially (Figure 2; left). The largest differences between marker sets in ab/adduction were noticeable during the swing phase (approximately from 60% to 90% of the gait cycle) and corresponded with the occurrence of peak flexion values, a likely indication of cross-talk artefact. The magnitudes of these differences were quantified by RMSE values, which on average ranged between 0.1° and 12.7° (Table 2; in grey). All five marker set signals demonstrate a generally neutral varus/valgus alignment at the beginning and end of the gait cycle, with four out of five tending towards adduction during the swing phase (*MiKneeSoTA* tended slightly toward abduction instead). Standard deviations in ab/adduction were largest for *PiG*, reflecting increased variability across knees and/or trials for that marker set.

**TABLE 2:** Root-mean-square error (RMSE) values in degrees for flexion/extension, ab/adduction and int/external rotation before (grey), and after (green) REFRAME implementation for all possible pairwise combinations of the marker sets.

		RMSE vs				
		PiG	PiG wand	MA	KAD	MiKneeSoTA	
PiG	Flexion/Extension	-	3.6 ± 2.0	3.6 ± 2.0	3.6 ± 2.1	10.0 ± 4.8	
	Ab/Adduction	-	5.9 ± 3.2	5.8 ± 3.2	6.2 ± 3.6	8.7 ± 4.0	
	Int/External Rotation	-	7.9 ± 4.3	7.9 ± 4.3	12.7 ± 10.4	11.9 ± 6.3	
PiG wand	Flexion/Extension	0.9 ± 0.4	-	0.1 ± 0.1	2.4 ± 1.5	7.9 ± 4.3	
	Ab/Adduction	0.9 ± 0.4	-	0.3 ± 0.6	2.9 ± 1.7	4.7 ± 1.9	
	Int/External Rotation	1.6 ± 0.5	-	0.3 ± 0.2	8.9 ± 7.9	10.0 ± 5.2	
MA	Flexion/Extension	0.9 ± 0.4	0.1 ± 0.1	-	2.5 ± 1.5	7.9 ± 4.3	
	Ab/Adduction	1.0 ± 0.4	0.1 ± 0.1	-	2.9 ± 1.7	4.8 ± 1.9	
	Int/External Rotation	1.7 ± 0.5	0.2 ± 0.2	-	8.9 ± 8.0	10.0 ± 5.2	
KAD	Flexion/Extension	1.0 ± 0.4	0.2 ± 0.1	0.2 ± 0.1	-	8.9 ± 4.7	
	Ab/Adduction	1.0 ± 0.4	0.2 ± 0.1	0.2 ± 0.1	-	6.1 ± 2.4	
	Int/External Rotation	1.7 ± 0.5	0.4 ± 0.3	0.4 ± 0.3	-	10.2 ± 8.8	
MiKnee-SoTA	Flexion/Extension	3.3 ± 0.6	3.1 ± 0.4	3.2 ± 0.4	3.1 ± 0.4	-	
	Ab/Adduction	1.8 ± 0.7	1.3 ± 0.4	1.3 ± 0.4	1.3 ± 0.4	-	
	Int/External Rotation	3.9 ± 1.3	3.9 ± 1.3	3.9 ± 1.3	3.9 ± 1.3	-	

For int/external rotation, signal differences before REFRAME visibly resembled constant offsets across the entire gait cycle for *PiG wand*, *MA* and *KAD*. The same is true for the *PiG* signal during most of the gait cycle, except the swing phase, where the angle offset relative to the other signals is clearly not constant. Int/external rotation angles as estimated by *MiKneeSoTA* were the most constant throughout the entire gait cycle, with fewer fluctuations than the other marker sets, and demonstrating a smaller standard deviation.

As part of REFRAME optimisation, rotational transformations were applied to the femoral and tibial local reference frames associated with each marker set. Mean non-zero transformation absolute magnitudes varied between 0.2° and 15.3° (Table 3). The resulting optimised kinematic signals demonstrated considerable improvement in agreement across marker sets after REFRAME implementation (Figure 2; right). The observed improvement in agreement between kinematic signals was corroborated by RMSE values ranging on average between 0.1° and 3.9° after REFRAME (Table 2; in green). Kinematic signals for four out of five marker sets were effectively in full agreement after frame optimisation, with *MiKneeSoTA* being generally similar on average, but with fewer fluctuations, especially at heel strike and during the swing phase.

**TABLE 3:** Mean REFRAME transformations (in degrees) with standard deviations for the femoral (top) and tibial (bottom) segment reference frame. Rotations are given around the raw axes of the segment frames pointing laterally (x), anteriorly (y) and proximally (z).

	Femoral frame transformations				
	PiG	PiG wand	MA	KAD	MiKneeSoTA
Rx [°]	−0.6 ± 1.8	−2.9 ± 2.8	−3.0 ± 2.8	−1.4 ± 3.6	−8.2 ± 6.6
Ry [°]	4.1 ± 9.5	1.1 ± 5.4	1.2 ± 5.2	0.6 ± 5.9	5.3 ± 4.9
Rz [°]	−15.3 ± 14.5	−2.7 ± 8.8	−2.8 ± 8.8	−6.0 ± 9.4	0.9 ± 5.8

	Tibial Frame Transformations				
	PiG	PiG wand	MA	KAD	MiKneeSoTA
Rx [°]	0.0 ± 0.0	0.0 ± 0.0	0.0 ± 0.0	0.0 ± 0.0	0.0 ± 0.0
Ry [°]	3.0 ± 9.6	0.3 ± 5.1	0.3 ± 5.2	−0.2 ± 5.3	5.7 ± 6.5
Rz [°]	−8.9 ± 14.6	−4.0 ± 10.0	−4.0 ± 10.0	−12.1 ± 13.6	0.7 ± 6.3

## 4 Discussion

In this study, we explored the hypotheses that: 1) optical marker-based motion capture could lead to visually different sets of rotational kinematic signals for a common underlying motion depending on the specific marker set used, and 2) REFRAME implementation could quantify how much of these differences could have resulted from inconsistent reference frame orientations between marker sets. Differences in the tibio-femoral rotation signals of 30 knees captured during treadmill walking using five distinct marker sets were systematically evaluated both before and after implementation of the REFRAME approach. Our findings indicated that the use of different marker sets indeed initially result in different kinematic signals for a common underlying joint motion pattern, and that in the absence of dedicated post-processing protocols to e.g., account for non-rigid marker displacements (i.e., soft-tissue artefacts) these differences principally stem from differences in local reference frame orientation, as demonstrated by signal convergence after REFRAME.

Prior to REFRAME implementation, the sets of kinematic signals associated with the five different marker sets (Figure 2; left) corroborate the findings of Kerkhoff et al. (2020) and Ferrari et al. (2008), illustrating that the choice of marker set can indeed influence raw (i.e., non-optimised or non-standardised) gait analysis results. Moreover, “contradictory” patterns in ab/adduction, as previously reported by these studies, were also observed in our results, with mean curves for *PiG*, *PiG wand*, *MA* and *KAD* tending towards adduction during the swing phase, and *MiKneeSoTA* tending towards abduction (Figure 2; left). Knee-specific results (Supplementary Figures S2–S31) revealed similar effects were observable in marker sets other than *MiKneeSoTA*; e.g., mean curves for knee 2 showed *PiG* clearly tended towards abduction during the swing phase, while *KAD* instead tended towards adduction (Supplementary Figures S2).

Results after REFRAME implementation strongly supported the hypothesis that initial differences between the kinematic signals associated with distinct marker sets were merely the result of inconsistencies in joint axis alignments. Moreover, these differences in kinematic signals could be easily reconciled with known distinctions between the marker sets. These effects were especially evident for int/external rotation angles (as described in the following paragraphs), which are a direct result of the orientation of the tibial frame’s mediolateral axis relative to the analogous femoral frame axis.

**FIGURE 3:**
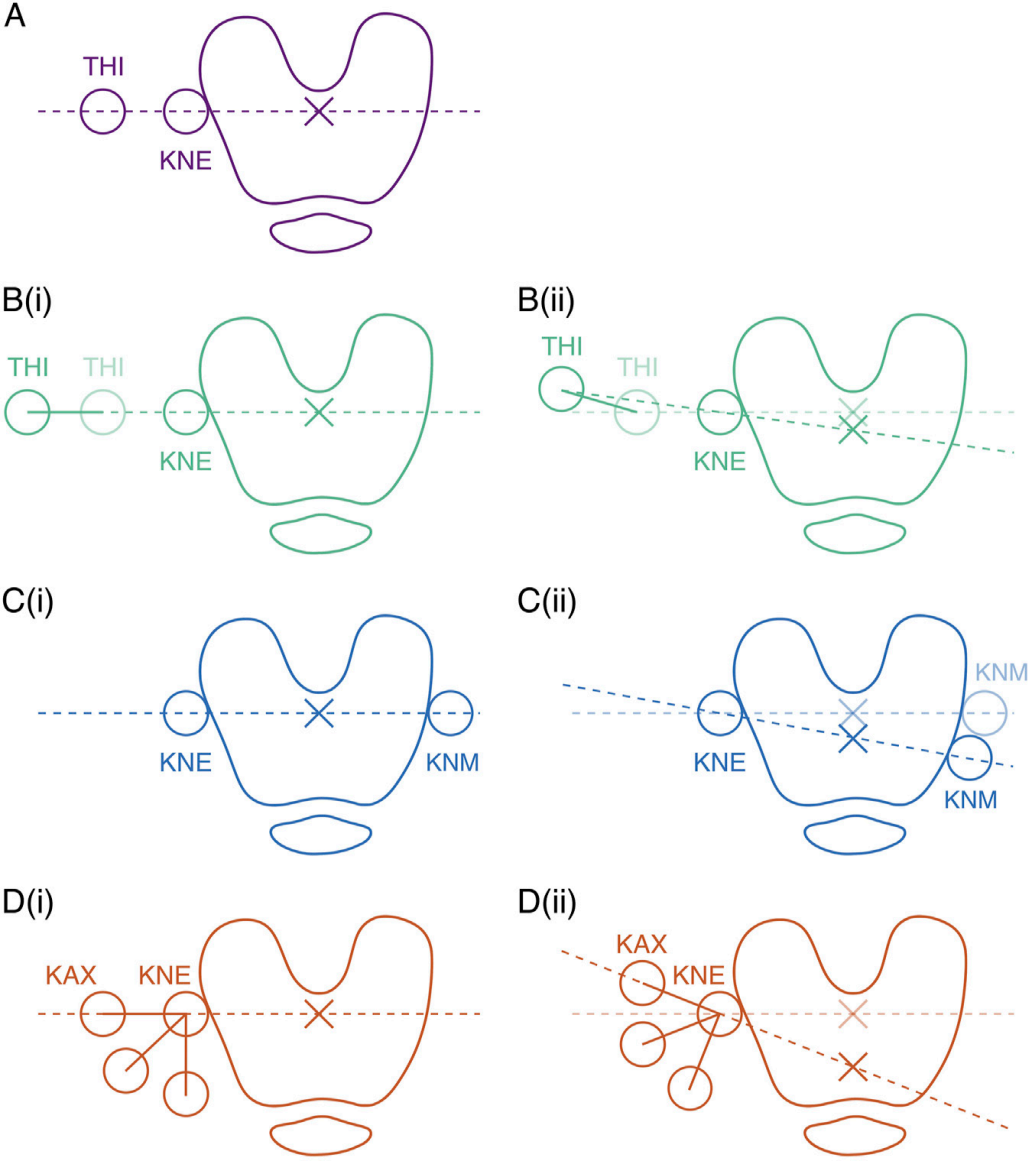
Markers influencing the orientation of the femoral mediolateral axis in the transverse plane for **(A)** *PiG*, **(B)** *PiG wand*, **(C)** *MA*, and **(D)** KAD. In **(B–D)**, **(i)** illustrates marker configurations assuming identical axis alignment than with *PiG*, while **(ii)** illustrates marker configurations assuming a different axis alignment that with *PiG*. Adapted from Figure 11.4 in (Baker, 2013). Note: figure is not to scale.

In the case of *PiG*, the orientation of the femoral mediolateral axis in the transverse plane is defined by two key markers: THI and KNE (Figure 3A). The same applies to *PiG* *wand*, with the main difference that rather than being a skin marker, THI is instead at the end of a *wand* (Figure 3B). The underlying goal of the *wand* is to improve the accuracy with which the frontal femur plane is defined (Leboeuf and Sangeux, 2023). If the THI skin marker of the *PiG* marker set had already been “accurately” positioned to begin with, then *PiG wand* would logically find the same femoral mediolateral axis (Figure 3Bi), and therefore the same int/external rotation angle as with *PiG.* The assumption, however, is that *PiG* wand finds a *more* accurate frontal plane than *PiG*, based on a “better” positioned THI wand marker. Average signals across subjects and trials show a more internally rotated knee (tibia relative to femur; i.e., a more externally rotated femoral ML axis) with *PiG wand* than with *PiG* (Figure 2), suggesting a more posteriorly positioned THI wand marker vs THI skin marker (Figure 3Bii).

The *MA* marker set relies on the same KNE skin marker as *PiG* and *PiG wand* to determine the orientation of the femoral ML axis in the transverse plane. However, instead of the THI marker, *MA* considers the position of an additional medial femoral condyle marker (KNM) to define the primary joint axis (Figure 3C). If the KNM marker were to lie along the line that crosses both THI and KNE, this would lead to the same femoral ML axis, and by extension also the same int/external knee angles, as *PiG* and *PiG wand* (Figure 3Ci). The average int/external rotation signals (Figure 2) indicate the *MA* marker set tended to estimate a more internally rotated knee than *PiG*, and only very slightly more internally rotated knee than *PiG wand*, which in turn suggest a more anteriorly positioned KNM marker (Figure 3Cii). Notably, for most knees, *PiG wand* and *MA* usually yielded extremely similar results (RMSEs <1°, or as little as < 0.1° for knees 4-6, 12-14, and 19-21; Supplementary Figures S5–S7S13–S15, S20–S22; Supplementary Tables S14, S17, S20. S38, S41, S44, S59, S62, S56), even before REFRAME implementation, suggesting the kinematics stemming from these two systems could be considered somewhat comparable even in the absence of methods like REFRAME. Nevertheless, this was not always the case (e.g., knees 17, 18; Supplementary Figures S18 and S19; Supplementary Tables S53, S56), so REFRAME application is still recommended to reach reliable conclusions.

**FIGURE 4:**
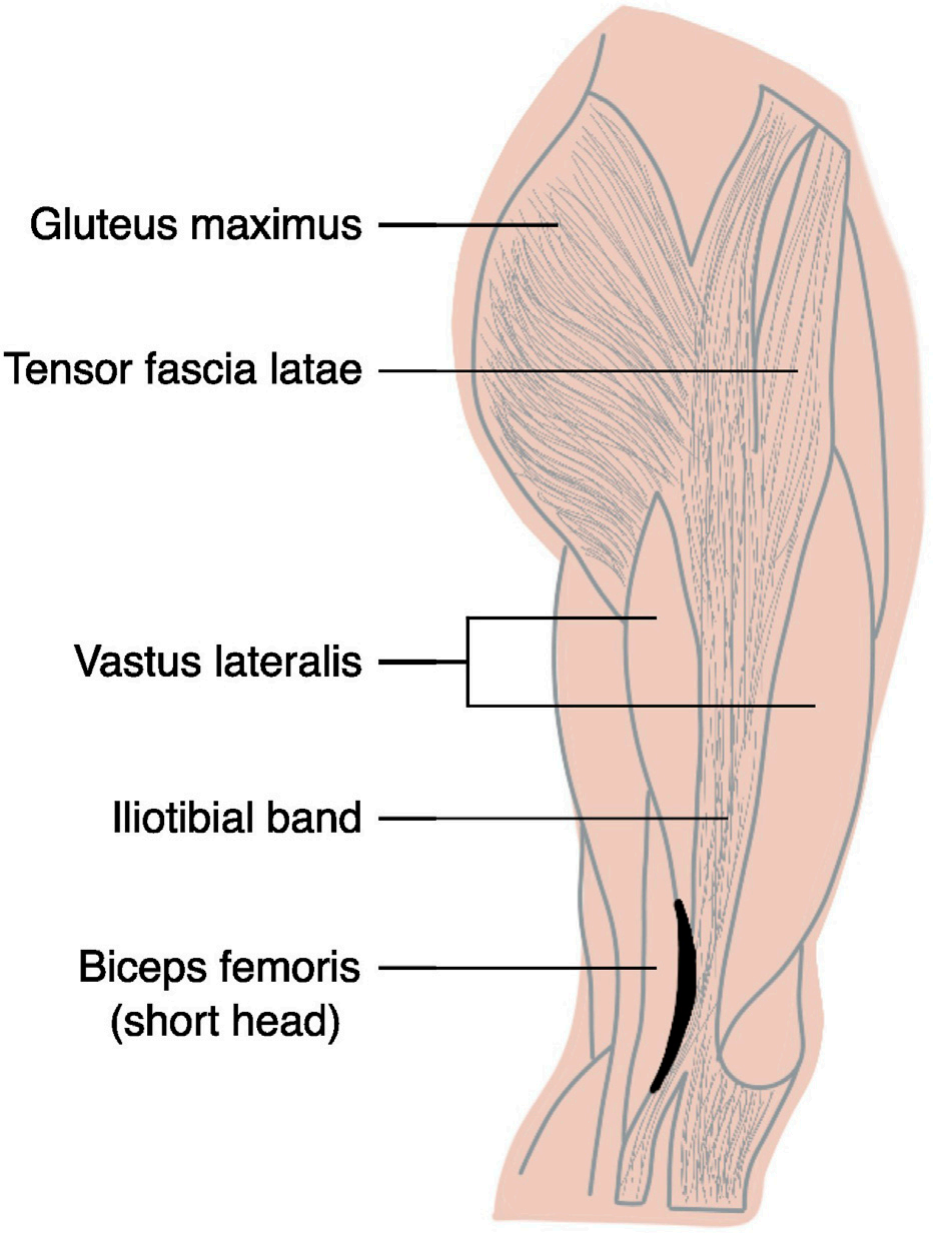
Muscular anatomy of the lateral thigh. Shaded black area designates the groove between the iliotibial band and the biceps femoris where the lateral jaw of the KAD’s clamp is hypothesised to have settled into. See accompanying video footage provided in Supplementary Video S1, especially between 00:01:50 and 00:02:00.

Finally, instead of using the THI and KNE skin markers, the *KAD* marker set relies on a dedicated alignment device with three physical markers (KAX, KD1 and KD2) and one virtual marker (KNE). The orientation of the femoral ML axis in the transverse plane with KAD is then determined by the position of KAX and the virtual KNE marker (Figure 3Di). On average, *KAD* angles for int/external rotation were the highest (i.e., most internally rotated) across all marker sets. If we were to continue assuming consistently oriented tibial frames for all marker sets, *KAD* would hence have to be associated with the most externally rotated femoral ML axis (Figure 3Dii). However, as demonstrated by the frame transformation values applied to the femoral reference frame with REFRAME (Table 3), the *KAD*-based femoral frame had to be externally rotated (indicated by the negative mean Rz value for *KAD*) to achieve signal convergence with the other marker sets, implying that the KAD-based frame had had a more internally rotated orientation to begin with. A potential explanation for a more internally rotated knee axis with *KAD* relates to the device’s attachment mechanism. The KAD component resembles a clamp, which users are instructed to carefully place by ensuring the external pad lays on the same point on which the KNE skin marker would be [i.e., the lateral femoral epicondyle (see page 16 in (Vicon, 2021)]. The spring force effectively pulls the jaws of the clamp towards each other in such a way that the lateral contact point favours as medial a position as possible. The external pad could plausibly thus be pulling the skin slightly posteriorly such that the lateral jaw of the clamp can settle into the groove that lies between the iliotibial band and the bicep femoris (Figure 4; Supplementary Video S1). Upon removal of the KAD contraption in preparation for dynamic trials, that same patch of skin that was directly in contact with the clamp’s lateral jaw would be free to return to its natural, purportedly more anterior position, leading to a femoral knee axis that is perceived as internally rotated relative to the reference pose, despite actually being unchanged. Briefly, the reason for this discrepancy between the expected externally rotated *KAD* femoral axis and the kinematic signals that instead suggest a more internally rotated femoral frame instead lies in the initial orientation of the tibial frame. Presumably *even more* internally rotated in comparison, the orientation of the tibial frame could therefore logically explain why the *KAD* model was still associated with the most internally rotated knees (Supplementary Figure S1).

Up until this point, the relationship between joint angle differences and frame orientation differences had been simplified by modelling all effects on the femoral frame only. Realistically, however, differences in joint angles will result from inconsistent reference frame orientation for both the femoral and tibial segments. This is effectively illustrated by the values of the frame transformations applied during REFRAME analysis, which corroborate our previous inferences of the likely relative orientations of the local segment frames estimated by each marker set. For example, according to Figure 2, there is approximately 12° difference between the int/external knee angle estimated by *PiG* vs. *KAD* at the beginning of the gait cycle (close to 0° flexion). Similarly, Table 3 shows that, on average, REFRAME implementation to improve convergence led to rotating both *PiG* and *KAD* femoral reference frames around their respective z-axes with a net absolute difference of 8° (*PiG* rotated −14.3° minus *KAD* rotated −6.0° = −8.3°). For the tibial frames, the net absolute difference was approximately 4° (*PiG* rotated −8.2° minus *KAD* rotated −12.1 = 3.9°). Considering joint angles are given by the relative orientation of the tibial relative to the femoral segment, this ultimately yields a difference of 12° total, as initially suggested by Figure 2. Our results thus clearly demonstrate that a 12° “misalignment” between marker set reference frames around the longitudinal axis likely explains the initially observed differences between “raw” kinematic signals.

Notably, although four out of the five marker sets exhibit clearly signal convergence after REFRAME, visible differences remain for *MiKneeSoTA*. Although the mean ab/adduction and int/external rotation *MiKneeSoTA* values (both zero) after REFRAME were consistent with the results of the other marker sets (naturally, as our implementation of REFRAME minimised the RMS of both ab/adduction and int/external rotation), kinematic signals associated with *MiKneeSoTA* appeared generally more stable throughout the gait cycle and less prone to fluctuations. One hypothesis proposes that the signal fluctuations present in *PiG*, *PiG wand*, *MA*, and *KAD,* which are most noticeable in the first 20% of the gait cycle (approximately during the loading response), and between 60% and 80% of the gait cycle (approximately peak flexion) are the result of soft-tissue artefact. Given that the *MiKneeSoTA* model is associated with a dedicated algorithm for joint angle calculations that purposefully seeks to eliminate soft-tissue artefacts, this could explain the observed differences. Video footage capturing marker displacement relative to the underlying bones has been previously provided (Einfeldt et al., 2024), and technicians have reported observing a subtle oscillatory effect of some markers in certain dynamic conditions. This explanation is of course purely speculative and based on anecdotal evidence; the extent to which *MiKneeSoTA* successfully accounts for soft-tissue artefact *in vivo* still needs to be evaluated against moving dual-plane fluoroscopy in upcoming studies. Importantly, the exact implementations of the *PiG, PiG wand, MA*, and *KAD*, marker models used in this study did not include analogous post-processing methods to mitigate soft-tissue artifacts errors in marker data. Reference frame alignments were instead defined directly based on marker coordinates for these four marker sets. We presume that additionally processing the data from *PiG, PiG wand, MA,* and/or *KAD* to better fulfill the assumption of rigid body segments (using the cylinder-based model introduced with *MiKneeSoTA*, or past alternatives [ref PCT, OCST]) could potentially lead to signals that also converge with the optimised *MiKneeSoTA* kinematics after REFRAME.

Further relevant limitations that should be considered when interpreting the findings of this study include the notion that the different markers were likely affected by different magnitudes and patterns of soft-tissue artefact (for example, depending on the amount of muscle and other soft-tissue between a given marker and the underlying bone). As a result, it is very likely that the relative orientations of the local reference frames for any of the marker sets (except for *MiKneeSoTA*) would not always have been constant relative to each other over time. In contrast, the frame transformations prompted by REFRAME as part of the optimisation process would have been constant values for the entire activity cycle. Consequently, unless data had been pre-processed to ensure rigid body behaviour for all markers on a given limb segment (as with *MiKneeSoTA)*, we can expect the level of convergence achieved between reference frames from different marker sets after REFRAME optimisation may have varied slightly over the duration of each processed trial.

In this manner, this study demonstrates REFRAME’s potential to facilitate the comparison of kinematic signals of the knee joint, even for datasets stemming from laboratories with different marker sets and protocols. By accounting for signal differences that could be explained by the use of different reference frame definitions (here specifically orientations), REFRAME could help highlight any remaining “real” differences in motion. REFRAME implementation could therefore play a valuable role in enabling a more reliable comparison of kinematic signals stemming from laboratories that use different marker set protocols.

## Data Availability

The raw data supporting the conclusions of this article will be made available by the authors, without undue reservation.
