# Supplementary material for: Assessing the implementation of the REference FRame Alignment MEthod to compare differences in tibio-femoral kinematics during gait using five different marker sets
Source: Front Bioeng Biotechnol. 2025 Apr 2;13:1530365. doi: 10.3389/fbioe.2025.1530365 (PMC12000083; doi:10.3389/fbioe.2025.1530365)
Supplement: Supplementary file 1 [file DataSheet1.pdf]

Supplementary Material

Assessing the implementation of the REference FRame Alignment MEthod to compare differences in tibio-femoral kinematics during gait using five different marker sets

Ariana Ortigas-Vásquez[1,2], Ann-Kathrin Einfeldt[3], Yasmin Haufe[3], Michael Utz[1], Eike Jakubowitz[3], Adrian Sauer[1]*

[1] Research and Development, Aesculap AG, Tuttlingen, Germany;
[2] Musculoskeletal University Center Munich, Department of Orthopaedic and Trauma Surgery, Campus Grosshadern, Ludwig Maximilians University Munich, Marchioninistraße 15, 81377 Munich, Germany;
[3] Laboratory for Biomechanics and Biomaterials, Department of Orthopaedic Surgery, Hannover Medical School, Anna-von-Borries-Str. 1-7, 30625 Hannover, Germany;

Corresponding author: adrian.sauer@aesculap.de

Contents

1 Additional explanatory figures **3**

2 RMS values before and after REFRAME over whole dataset **4**

3 Individual subject results **5**

3.1 Right Knee of Subject 1 5
3.2 Left Knee of Subject 1 7
3.3 Right Knee of Subject 2 9
3.4 Left Knee of Subject 2 11
3.5 Right Knee of Subject 3 13
3.6 Left Knee of Subject 3 15
3.7 Right Knee of Subject 4 17
3.8 Left Knee of Subject 4 19
3.9 Right Knee of Subject 5 21
3.10 Left Knee of Subject 5 23
3.11 Right Knee of Subject 6 25
3.12 Left Knee of Subject 6 27
3.13 Right Knee of Subject 7 29
3.14 Left Knee of Subject 7 31
3.15 Right Knee of Subject 8 33
3.16 Left Knee of Subject 8 35
3.17 Right Knee of Subject 9 37
3.18 Left Knee of Subject 9 39
3.19 Right Knee of Subject 10 41
3.20 Left Knee of Subject 10 43
3.21 Right Knee of Subject 11 45
3.22 Left Knee of Subject 11 47
3.23 Right Knee of Subject 12 49
3.24 Left Knee of Subject 12 51
3.25 Right Knee of Subject 13 53
3.26 Left Knee of Subject 13 55
3.27 Right Knee of Subject 14 57
3.28 Left Knee of Subject 14 59
3.29 Right Knee of Subject 15 61
3.30 Left Knee of Subject 15 63

1 Additional explanatory figures

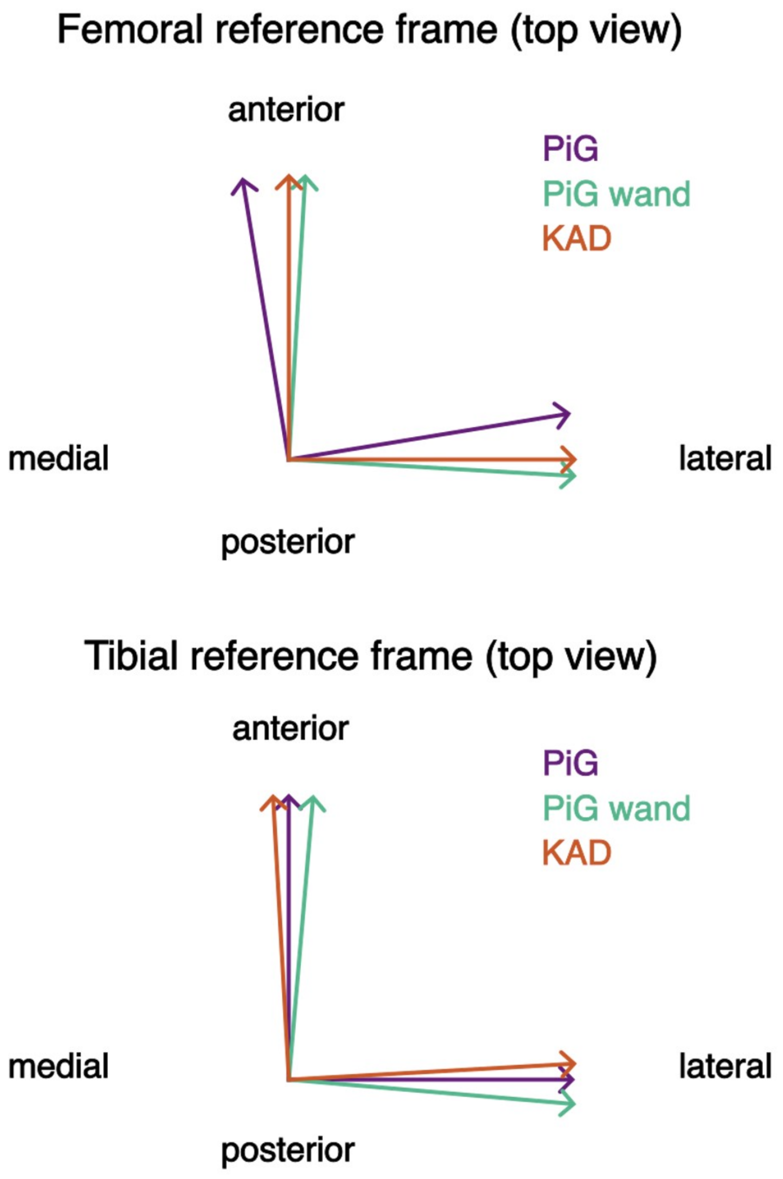

Supplementary Figure S1: Illustration of the possible relative orientations between the raw local reference frames of PiG, PiG wand, and KAD for both the femur (top) and tibia (bottom) segments (both from a top view), showing how it would be possible for the KAD femoral reference frame not to be most externally rotated while being associated with the most internally rotated mean kinematic signal (see Figure 2 in main article). The reason lies in the relative orientation of the KAD tibial reference frame, which is so internally rotated relative to the other marker sets that it still results in the most internally rotated orientation of the tibial relative to the femoral frame.

2 RMS values before and after REFRAME over whole dataset

**RMS**		Raw data	REFRAMEd data
PiG	Abd/Add	8.85 ± 3.97	1.88 ± 0.65
	Ext/Int	10.50 ± 5.51	3.18 ± 1.20
PiG wand	Abd/Add	4.50 ± 2.57	1.33 ± 0.37
	Ext/Int	8.96 ± 4.61	3.25 ± 1.15
MA	Abd/Add	4.37 ± 2.64	1.34 ± 0.37
	Ext/Int	8.96 ± 4.57	3.28 ± 1.12
KAD	Abd/Add	5.31 ± 2.81	1.30 ± 0.35
	Ext/Int	9.60 ± 9.39	3.26 ± 1.12
MiKneeSoTA	Abd/Add	3.94 ± 2.04	0.70 ± 0.20
	Ext/Int	3.26 ± 1.25	1.55 ± 0.45

Supplementary Table S1: RMS (RMSE vs. 0) of all datasets before and after application of REFRAME for the out-of-sagittal-plane rotations.

3 Individual subject results

3.1 Right Knee of Subject 1

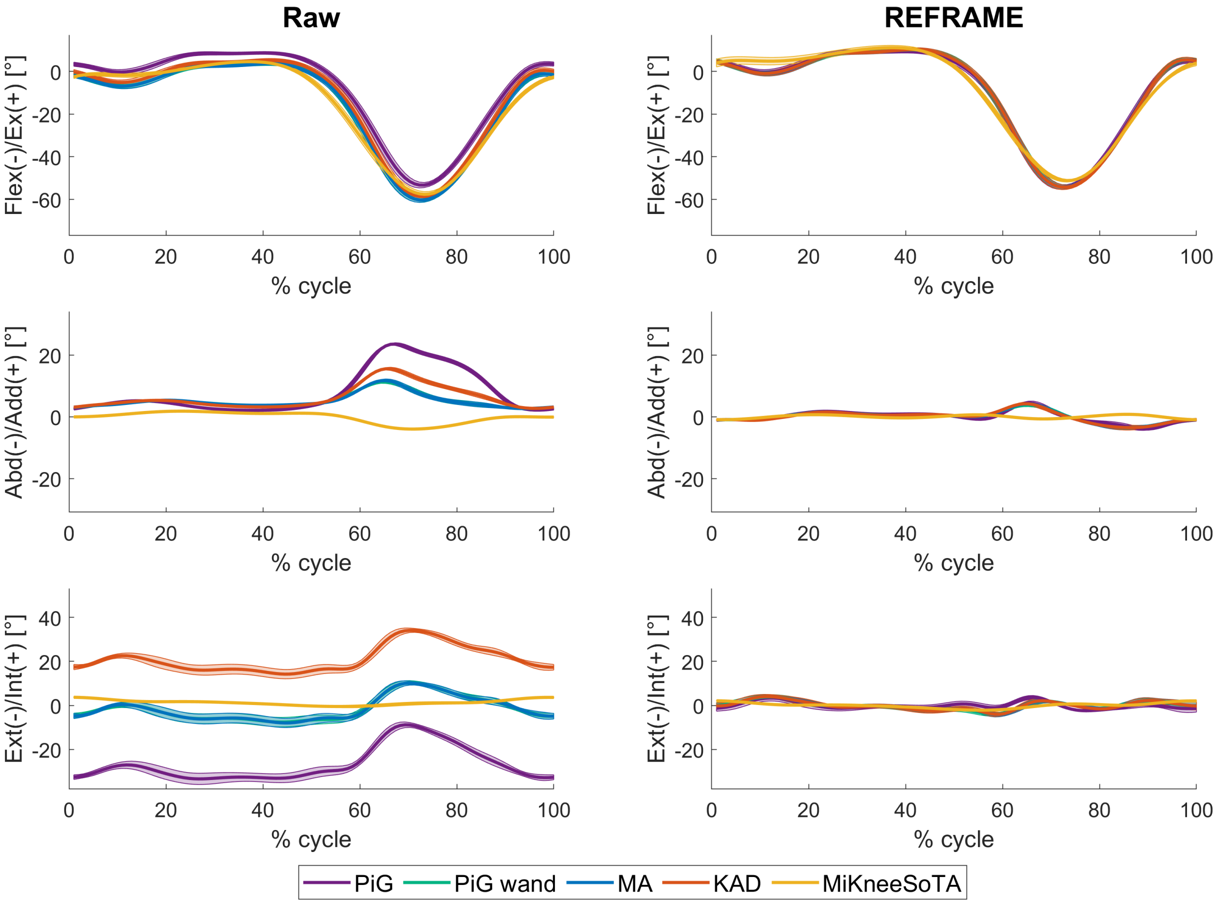

Supplementary Figure S2: Mean kinematics for all 5 markersets with standard deviation over all nine steps for knee 1 (subject 1, right). Left Column: raw kinematics, right column: REFRAMEd kinematics.

	Femur					Tibia				
markerset	PiG	PiG wand	MA	KAD	MiKneeSoTA	PiG	PiG wand	MA	KAD	MiKneeSoTA
Rx	4.0 ± 0.7	-5.8 ± 0.2	-5.8 ± 0.2	-6.7 ± 0.3	-6.5 ± 1.1	0.0 ± 0.0	0.0 ± 0.0	0.0 ± 0.0	0.0 ± 0.0	0.0 ± 0.0
Ry	12.2 ± 1.0	11.4 ± 1.3	11.3 ± 1.2	11.8 ± 1.4	0.7 ± 0.6	7.9 ± 1.1	6.6 ± 1.1	6.6 ± 1.1	7.0 ± 1.3	0.1 ± 0.6
Rz	-26.2 ± 0.7	-8.4 ± 0.6	-8.4 ± 0.6	-13.1 ± 0.5	4.8 ± 0.4	4.8 ± 0.9	-4.6 ± 0.9	-4.6 ± 0.9	-31.8 ± 0.9	3.1 ± 0.5

Supplementary Table S2: Transformations of segment frames for REFRAMEd datasets in the curves above.

**Raw data**		RMS	RMSE vs.			
			PiG wand	MA	KAD	MiKneeSoTA
PiG	Flex/Ex	22.35 ± 0.68	6.41 ± 0.07	6.41 ± 0.07	4.65 ± 0.07	7.30 ± 1.26
	Abd/Add	10.64 ± 0.16	6.16 ± 0.12	6.15 ± 0.12	4.07 ± 0.08	11.94 ± 0.20
	Ext/Int	27.78 ± 1.22	25.30 ± 0.17	25.31 ± 0.18	47.73 ± 0.17	29.20 ± 1.27
PiG wand	Flex/Ex	26.25 ± 0.69	-	0.19 ± 0.01	1.80 ± 0.01	3.49 ± 0.73
	Abd/Add	5.54 ± 0.12	-	0.14 ± 0.01	2.11 ± 0.05	6.38 ± 0.20
	Ext/Int	5.70 ± 0.69	-	0.31 ± 0.05	22.55 ± 0.02	6.35 ± 0.85
MA	Flex/Ex	26.27 ± 0.69	-	-	1.79 ± 0.01	3.56 ± 0.72
	Abd/Add	5.56 ± 0.12	-	-	2.10 ± 0.04	6.40 ± 0.20
	Ext/Int	5.70 ± 0.68	-	-	22.54 ± 0.02	6.35 ± 0.83
KAD	Flex/Ex	25.25 ± 0.68	-	-	-	4.06 ± 0.92
	Abd/Add	7.18 ± 0.12	-	-	-	8.25 ± 0.19
	Ext/Int	21.79 ± 1.01	-	-	-	20.53 ± 1.10
MiKneeSoTA	Flex/Ex	26.23 ± 0.80	-	-	-	-
	Abd/Add	1.79 ± 0.14	-	-	-	-
	Ext/Int	1.86 ± 0.14	-	-	-	-

Supplementary Table S3: RMS (RMSE vs. 0) of all datasets and the RMSEs for all combinations for the datasets shown above before REFRAME.

**REFRAMEd**		RMS	RMSE vs.			
			PiG wand	MA	KAD	MiKneeSoTA
PiG	Flex/Ex	23.11 ± 0.68	0.65 ± 0.06	0.64 ± 0.07	0.63 ± 0.07	3.28 ± 0.65
	Abd/Add	1.90 ± 0.16	0.54 ± 0.03	0.50 ± 0.03	0.54 ± 0.03	2.07 ± 0.19
	Ext/Int	1.86 ± 1.22	1.66 ± 0.12	1.66 ± 0.13	1.60 ± 0.12	2.30 ± 0.33
PiG wand	Flex/Ex	23.21 ± 0.69	-	0.19 ± 0.02	0.20 ± 0.02	3.47 ± 0.61
	Abd/Add	1.79 ± 0.12	-	0.14 ± 0.01	0.20 ± 0.02	1.96 ± 0.22
	Ext/Int	2.08 ± 0.69	-	0.30 ± 0.04	0.32 ± 0.04	1.72 ± 0.37
MA	Flex/Ex	23.23 ± 0.69	-	-	0.06 ± 0.01	3.54 ± 0.60
	Abd/Add	1.84 ± 0.12	-	-	0.14 ± 0.04	2.01 ± 0.22
	Ext/Int	2.14 ± 0.68	-	-	0.11 ± 0.02	1.80 ± 0.37
KAD	Flex/Ex	23.28 ± 0.68	-	-	-	3.54 ± 0.59
	Abd/Add	1.87 ± 0.12	-	-	-	2.03 ± 0.23
	Ext/Int	2.13 ± 1.01	-	-	-	1.82 ± 0.38
MiKneeSoTA	Flex/Ex	23.04 ± 0.80	-	-	-	-
	Abd/Add	0.54 ± 0.14	-	-	-	-
	Ext/Int	1.18 ± 0.14	-	-	-	-

Supplementary Table S4: RMS (RMSE vs. 0) of all datasets and the RMSEs for all combinations for the datasets shown above after REFRAME.

3.2 Left Knee of Subject 1

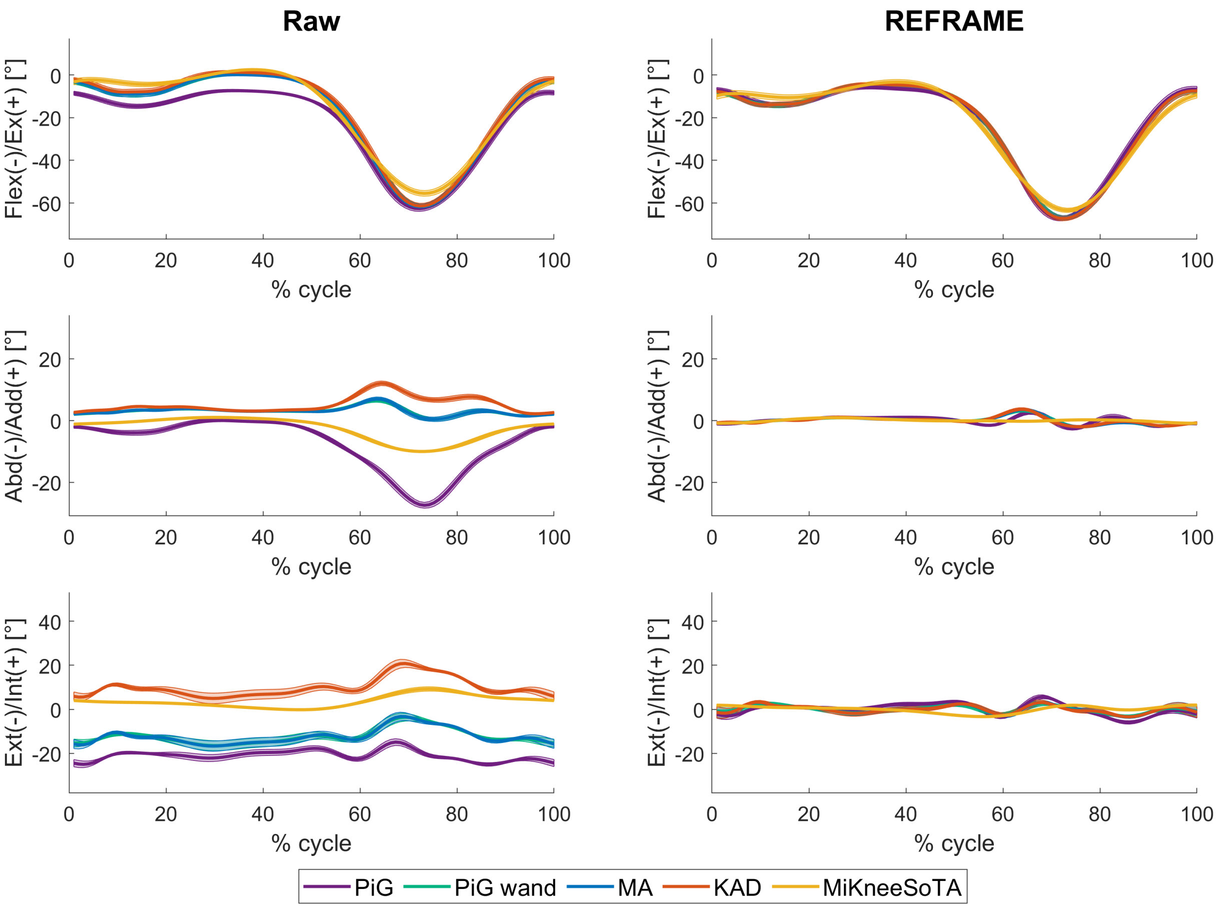

Supplementary Figure S3: Mean kinematics for all 5 markersets with standard deviation over all nine steps for knee 2 (subject 1, left). Left Column: raw kinematics, right column: REFRAMEd kinematics.

	Femur					Tibia				
markerset	PiG	PiG wand	MA	KAD	MiKneeSoTA	PiG	PiG wand	MA	KAD	MiKneeSoTA
Rx	4.9 ± 0.9	6.2 ± 0.6	6.2 ± 0.6	5.1 ± 0.3	5.8 ± 0.9	0.0 ± 0.0	0.0 ± 0.0	0.0 ± 0.0	0.0 ± 0.0	0.0 ± 0.0
Ry	16.6 ± 1.9	7.4 ± 2.0	7.4 ± 1.9	6.2 ± 2.1	11.1 ± 0.7	16.9 ± 2.1	5.3 ± 2.0	5.3 ± 2.0	4.3 ± 2.1	10.3 ± 0.9
Rz	18.0 ± 0.7	-5.1 ± 0.8	-5.1 ± 0.8	-11.2 ± 0.6	5.6 ± 0.5	42.6 ± 0.7	10.7 ± 0.8	10.7 ± 0.8	-17.5 ± 0.9	5.2 ± 0.6

Supplementary Table S5: Transformations of segment frames for REFRAMEd datasets in the curves above.

**Raw data**		RMS	RMSE vs.			
			PiG wand	MA	KAD	MiKneeSoTA
PiG	Flex/Ex	29.55 ± 0.77	5.34 ± 0.14	5.36 ± 0.14	6.27 ± 0.15	7.26 ± 0.72
	Abd/Add	11.25 ± 0.35	13.46 ± 0.19	13.44 ± 0.19	16.43 ± 0.26	6.90 ± 0.41
	Ext/Int	21.16 ± 0.32	9.21 ± 0.33	9.21 ± 0.33	31.13 ± 0.43	24.99 ± 0.36
PiG wand	Flex/Ex	27.40 ± 0.77	-	0.19 ± 0.02	0.99 ± 0.01	3.49 ± 0.64
	Abd/Add	3.30 ± 0.16	-	0.15 ± 0.03	3.01 ± 0.07	6.82 ± 0.40
	Ext/Int	12.82 ± 0.78	-	0.49 ± 0.06	22.23 ± 0.04	16.19 ± 0.70
MA	Flex/Ex	27.42 ± 0.77	-	-	0.96 ± 0.01	3.57 ± 0.63
	Abd/Add	3.32 ± 0.15	-	-	3.02 ± 0.07	6.82 ± 0.40
	Ext/Int	12.84 ± 0.77	-	-	22.22 ± 0.04	16.21 ± 0.69
KAD	Flex/Ex	26.91 ± 0.77	-	-	-	3.39 ± 0.53
	Abd/Add	5.72 ± 0.27	-	-	-	9.74 ± 0.44
	Ext/Int	10.86 ± 0.58	-	-	-	6.99 ± 0.74
MiKneeSoTA	Flex/Ex	25.69 ± 0.79	-	-	-	-
	Abd/Add	4.50 ± 0.20	-	-	-	-
	Ext/Int	4.57 ± 0.36	-	-	-	-

Supplementary Table S6: RMS (RMSE vs. 0) of all datasets and the RMSEs for all combinations for the datasets shown above before REFRAME.

**REFRAMEd**		RMS	RMSE vs.			
			PiG wand	MA	KAD	MiKneeSoTA
PiG	Flex/Ex	30.68 ± 0.77	1.29 ± 0.08	1.25 ± 0.08	1.29 ± 0.07	3.43 ± 0.60
	Abd/Add	1.25 ± 0.35	0.89 ± 0.11	0.89 ± 0.12	0.94 ± 0.12	1.16 ± 0.10
	Ext/Int	2.76 ± 0.32	1.71 ± 0.23	1.71 ± 0.24	1.75 ± 0.24	3.33 ± 0.39
PiG wand	Flex/Ex	30.90 ± 0.77	-	0.21 ± 0.02	0.24 ± 0.02	2.89 ± 0.54
	Abd/Add	1.19 ± 0.16	-	0.15 ± 0.03	0.24 ± 0.03	1.16 ± 0.19
	Ext/Int	1.86 ± 0.78	-	0.48 ± 0.06	0.49 ± 0.06	2.35 ± 0.28
MA	Flex/Ex	30.92 ± 0.77	-	-	0.11 ± 0.02	2.98 ± 0.53
	Abd/Add	1.24 ± 0.15	-	-	0.16 ± 0.03	1.22 ± 0.19
	Ext/Int	2.02 ± 0.77	-	-	0.14 ± 0.01	2.49 ± 0.28
KAD	Flex/Ex	30.99 ± 0.77	-	-	-	3.00 ± 0.51
	Abd/Add	1.27 ± 0.27	-	-	-	1.25 ± 0.21
	Ext/Int	2.02 ± 0.58	-	-	-	2.54 ± 0.28
MiKneeSoTA	Flex/Ex	30.65 ± 0.79	-	-	-	-
	Abd/Add	0.52 ± 0.20	-	-	-	-
	Ext/Int	1.55 ± 0.36	-	-	-	-

Supplementary Table S7: RMS (RMSE vs. 0) of all datasets and the RMSEs for all combinations for the datasets shown above after REFRAME.

3.3 Right Knee of Subject 2

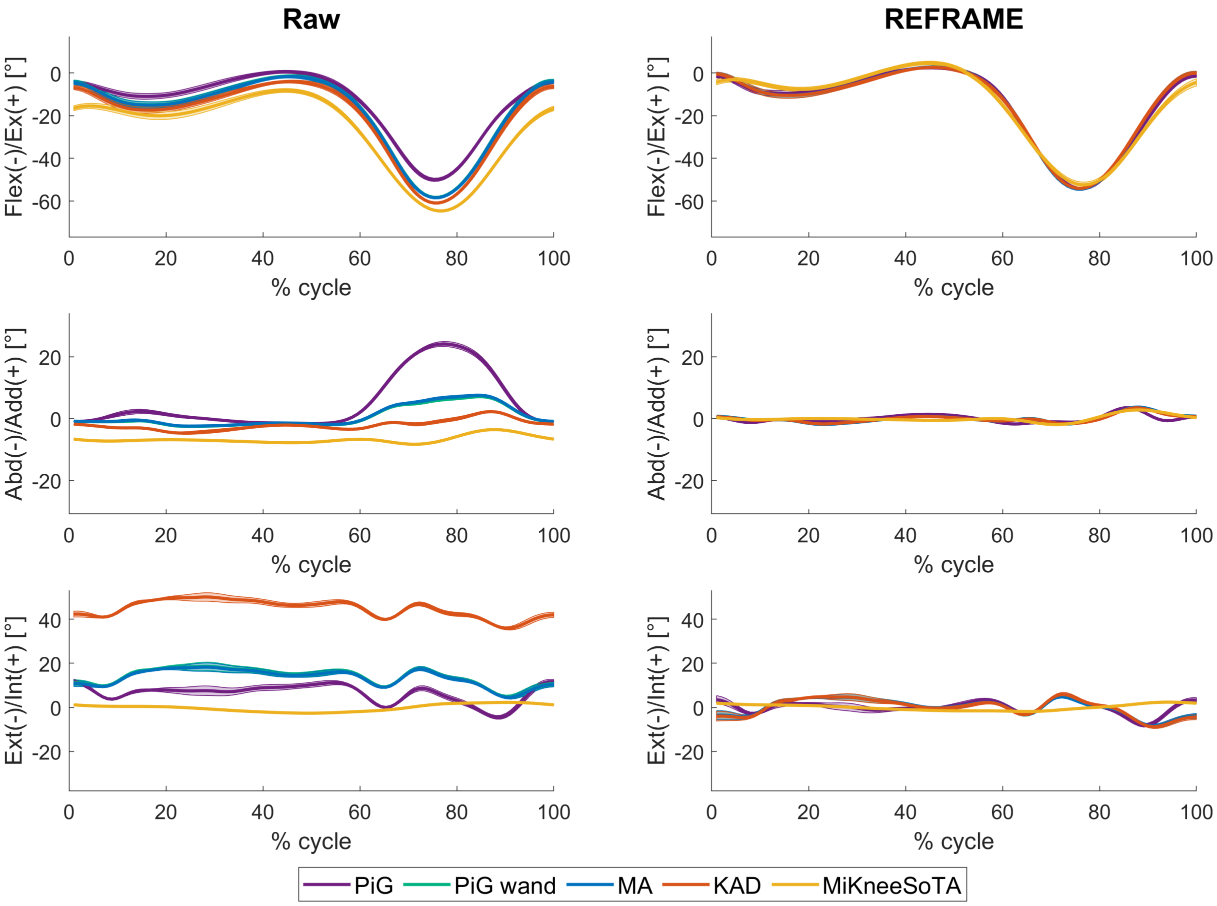

Supplementary Figure S4: Mean kinematics for all 5 markersets with standard deviation over all nine steps for knee 3 (subject 2, right). Left Column: raw kinematics, right column: REFRAMEd kinematics.

	Femur					Tibia				
markerset	PiG	PiG wand	MA	KAD	MiKneeSoTA	PiG	PiG wand	MA	KAD	MiKneeSoTA
Rx	0.9 ± 0.5	-3.0 ± 0.4	-3.2 ± 0.4	-2.3 ± 1.9	-12.4 ± 1.3	0.0 ± 0.0	0.0 ± 0.0	0.0 ± 0.0	0.0 ± 0.0	0.0 ± 0.0
Ry	-20.6 ± 2.3	-6.6 ± 1.8	-6.6 ± 1.8	-6.8 ± 1.9	2.5 ± 0.5	-19.1 ± 2.2	-4.9 ± 1.7	-5.0 ± 1.7	-6.0 ± 2.6	9.6 ± 0.5
Rz	-23.9 ± 0.6	-8.0 ± 0.3	-8.5 ± 0.3	-0.1 ± 0.5	-2.1 ± 0.3	-33.4 ± 0.8	-22.8 ± 0.8	-22.9 ± 0.7	-46.8 ± 0.7	-1.1 ± 0.3

Supplementary Table S8: Transformations of segment frames for REFRAMEd datasets in the curves above.

**Raw data**		RMS	RMSE vs.			
			PiG wand	MA	KAD	MiKneeSoTA
PiG	Flex/Ex	21.16 ± 0.40	4.82 ± 0.11	4.96 ± 0.11	7.17 ± 0.12	13.14 ± 1.10
	Abd/Add	10.18 ± 0.25	7.41 ± 0.13	7.21 ± 0.13	10.70 ± 0.16	14.73 ± 0.26
	Ext/Int	7.51 ± 0.56	8.23 ± 0.17	7.97 ± 0.17	38.58 ± 0.18	8.25 ± 0.60
PiG wand	Flex/Ex	25.68 ± 0.39	-	0.17 ± 0.00	2.60 ± 0.01	9.15 ± 1.06
	Abd/Add	3.28 ± 0.19	-	0.21 ± 0.00	3.38 ± 0.05	7.89 ± 0.14
	Ext/Int	14.33 ± 0.47	-	0.30 ± 0.01	30.94 ± 0.02	14.82 ± 0.52
MA	Flex/Ex	25.81 ± 0.39	-	-	2.43 ± 0.01	9.00 ± 1.06
	Abd/Add	3.42 ± 0.19	-	-	3.58 ± 0.06	8.09 ± 0.15
	Ext/Int	14.04 ± 0.48	-	-	31.23 ± 0.02	14.53 ± 0.52
KAD	Flex/Ex	27.70 ± 0.42	-	-	-	6.79 ± 0.96
	Abd/Add	2.73 ± 0.14	-	-	-	4.81 ± 0.12
	Ext/Int	44.90 ± 0.44	-	-	-	45.20 ± 0.49
MiKneeSoTA	Flex/Ex	32.57 ± 0.52	-	-	-	-
	Abd/Add	6.89 ± 0.07	-	-	-	-
	Ext/Int	1.64 ± 0.10	-	-	-	-

Supplementary Table S9: RMS (RMSE vs. 0) of all datasets and the RMSEs for all combinations for the datasets shown above before REFRAME.

**REFRAMEd**		RMS	RMSE vs.			
			PiG wand	MA	KAD	MiKneeSoTA
PiG	Flex/Ex	23.11 ± 0.40	0.89 ± 0.09	0.88 ± 0.09	0.92 ± 0.08	2.59 ± 0.47
	Abd/Add	1.22 ± 0.25	0.95 ± 0.04	0.95 ± 0.04	0.85 ± 0.06	1.06 ± 0.12
	Ext/Int	2.96 ± 0.56	3.01 ± 0.14	3.00 ± 0.14	3.13 ± 0.14	3.58 ± 0.25
PiG wand	Flex/Ex	23.10 ± 0.39	-	0.03 ± 0.00	0.13 ± 0.02	3.11 ± 0.52
	Abd/Add	1.32 ± 0.19	-	0.02 ± 0.00	0.14 ± 0.09	0.85 ± 0.06
	Ext/Int	3.74 ± 0.47	-	0.04 ± 0.00	0.35 ± 0.11	4.55 ± 0.41
MA	Flex/Ex	23.12 ± 0.39	-	-	0.15 ± 0.01	3.11 ± 0.52
	Abd/Add	1.33 ± 0.19	-	-	0.14 ± 0.09	0.85 ± 0.06
	Ext/Int	3.72 ± 0.48	-	-	0.36 ± 0.11	4.54 ± 0.41
KAD	Flex/Ex	23.00 ± 0.42	-	-	-	3.12 ± 0.54
	Abd/Add	1.23 ± 0.14	-	-	-	0.79 ± 0.07
	Ext/Int	3.91 ± 0.44	-	-	-	4.73 ± 0.45
MiKneeSoTA	Flex/Ex	23.28 ± 0.52	-	-	-	-
	Abd/Add	1.08 ± 0.07	-	-	-	-
	Ext/Int	1.36 ± 0.10	-	-	-	-

Supplementary Table S10: RMS (RMSE vs. 0) of all datasets and the RMSEs for all combinations for the datasets shown above after REFRAME.

3.4 Left Knee of Subject 2

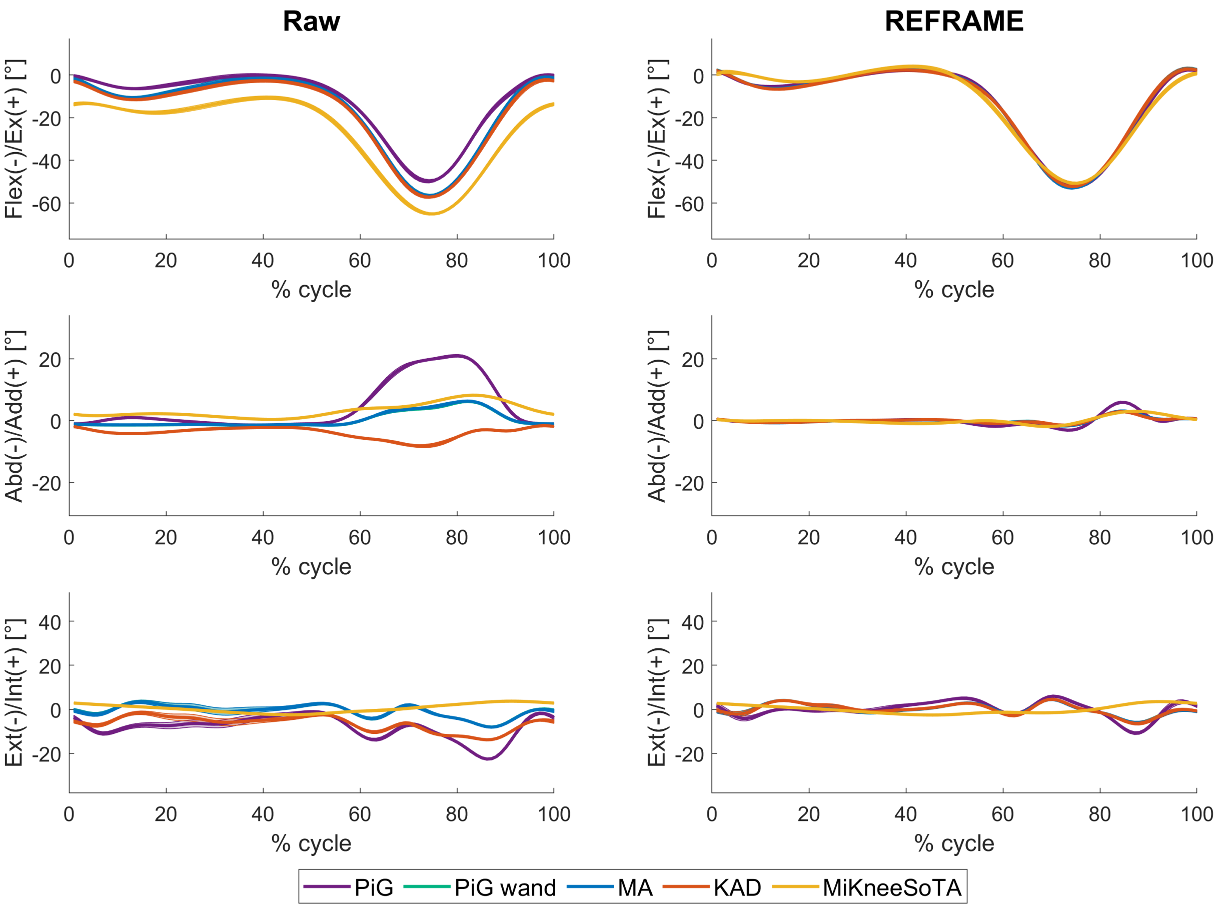

Supplementary Figure S5: Mean kinematics for all 5 markersets with standard deviation over all nine steps for knee 4 (subject 2, left). Left Column: raw kinematics, right column: REFRAMEd kinematics.

	Femur					Tibia				
markerset	PiG	PiG wand	MA	KAD	MiKneeSoTA	PiG	PiG wand	MA	KAD	MiKneeSoTA
Rx	-3.6 ± 0.2	-3.9 ± 0.1	-3.9 ± 0.1	-5.1 ± 0.1	-14.5 ± 0.7	0.0 ± 0.0	0.0 ± 0.0	0.0 ± 0.0	0.0 ± 0.0	0.0 ± 0.0
Ry	-18.6 ± 1.5	-6.2 ± 1.2	-6.2 ± 1.2	-4.9 ± 1.2	-0.2 ± 0.3	-17.8 ± 1.4	-4.9 ± 1.1	-4.9 ± 1.1	-2.2 ± 1.1	-2.0 ± 0.3
Rz	-21.9 ± 0.2	-5.8 ± 0.1	-5.9 ± 0.1	7.5 ± 0.1	-6.5 ± 0.4	-15.7 ± 0.8	-5.8 ± 0.7	-5.9 ± 0.7	12.5 ± 0.7	-6.6 ± 0.4

Supplementary Table S11: Transformations of segment frames for REFRAMEd datasets in the curves above.

Raw data		RMS	RMSE vs.			
			PiG wand	MA	KAD	MiKneeSoTA
PiG	Flex/Ex	20.30 ± 0.27	4.58 ± 0.06	4.60 ± 0.06	5.53 ± 0.07	14.94 ± 0.67
	Abd/Add	9.06 ± 0.12	6.91 ± 0.05	6.85 ± 0.05	12.56 ± 0.09	6.19 ± 0.15
	Ext/Int	9.82 ± 0.38	8.14 ± 0.11	8.12 ± 0.11	3.50 ± 0.08	11.00 ± 0.41
PiG wand	Flex/Ex	24.43 ± 0.26	-	0.03 ± 0.00	1.12 ± 0.02	11.00 ± 0.72
	Abd/Add	2.52 ± 0.12	-	0.08 ± 0.00	5.69 ± 0.05	3.12 ± 0.07
	Ext/Int	3.02 ± 0.20	-	0.05 ± 0.00	5.80 ± 0.03	4.45 ± 0.22
MA	Flex/Ex	24.45 ± 0.26	-	-	1.10 ± 0.02	10.98 ± 0.72
	Abd/Add	2.54 ± 0.11	-	-	5.76 ± 0.05	3.06 ± 0.08
	Ext/Int	3.03 ± 0.20	-	-	5.78 ± 0.03	4.47 ± 0.22
KAD	Flex/Ex	25.14 ± 0.26	-	-	-	9.92 ± 0.70
	Abd/Add	4.33 ± 0.09	-	-	-	7.93 ± 0.09
	Ext/Int	7.35 ± 0.36	-	-	-	8.47 ± 0.41
MiKneeSoTA	Flex/Ex	32.76 ± 0.42	-	-	-	-
	Abd/Add	3.89 ± 0.06	-	-	-	-
	Ext/Int	2.06 ± 0.11	-	-	-	-

Supplementary Table S12: RMS (RMSE vs. 0) of all datasets and the RMSEs for all combinations for the datasets shown above before REFRAME.

REFRAMEd		RMS	RMSE vs.			
			PiG wand	MA	KAD	MiKneeSoTA
PiG	Flex/Ex	22.14 ± 0.27	0.61 ± 0.06	0.60 ± 0.05	0.74 ± 0.08	2.50 ± 0.34
	Abd/Add	1.75 ± 0.12	0.86 ± 0.08	0.85 ± 0.08	0.91 ± 0.08	1.19 ± 0.09
	Ext/Int	3.74 ± 0.38	2.35 ± 0.08	2.34 ± 0.08	2.28 ± 0.08	5.10 ± 0.30
PiG wand	Flex/Ex	22.19 ± 0.26	-	0.03 ± 0.00	0.28 ± 0.02	2.71 ± 0.27
	Abd/Add	1.00 ± 0.12	-	0.01 ± 0.00	0.11 ± 0.01	0.79 ± 0.13
	Ext/Int	2.53 ± 0.20	-	0.04 ± 0.00	0.18 ± 0.01	3.80 ± 0.33
MA	Flex/Ex	22.19 ± 0.26	-	-	0.29 ± 0.02	2.71 ± 0.27
	Abd/Add	1.00 ± 0.11	-	-	0.11 ± 0.01	0.78 ± 0.13
	Ext/Int	2.54 ± 0.20	-	-	0.19 ± 0.01	3.82 ± 0.33
KAD	Flex/Ex	21.98 ± 0.26	-	-	-	2.77 ± 0.26
	Abd/Add	0.93 ± 0.09	-	-	-	0.81 ± 0.12
	Ext/Int	2.58 ± 0.36	-	-	-	3.85 ± 0.33
MiKneeSoTA	Flex/Ex	22.31 ± 0.42	-	-	-	-
	Abd/Add	1.20 ± 0.06	-	-	-	-
	Ext/Int	1.95 ± 0.11	-	-	-	-

Supplementary Table S13: RMS (RMSE vs. 0) of all datasets and the RMSEs for all combinations for the datasets shown above after REFRAME.

3.5 Right Knee of Subject 3

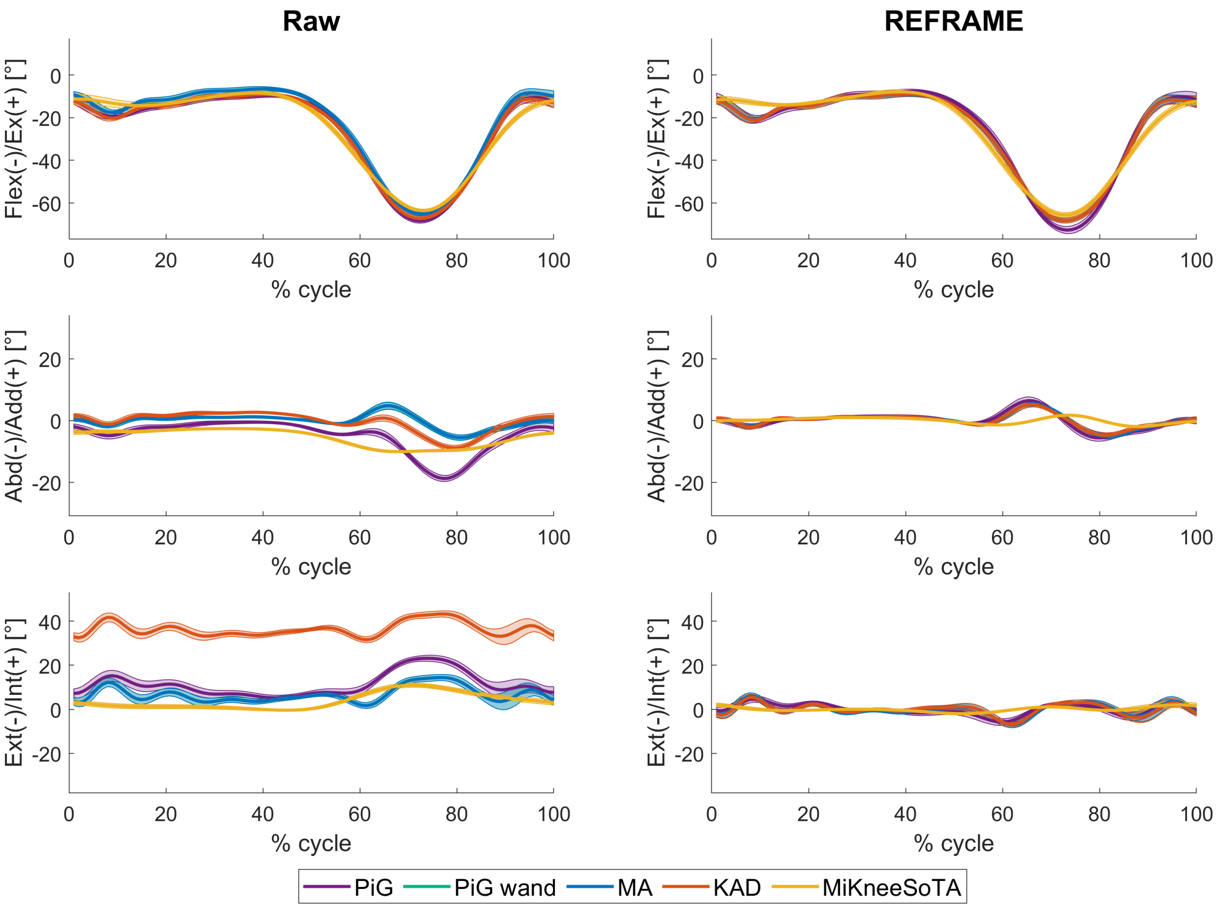

Supplementary Figure S6: Mean kinematics for all 5 markersets with standard deviation over all nine steps for knee 5 (subject 3, right). Left Column: raw kinematics, right column: REFRAMEd kinematics.

	Femur					Tibia				
markerset	PiG	PiG wand	MA	KAD	MiKneeSoTA	PiG	PiG wand	MA	KAD	MiKneeSoTA
Rx	-3.2 ± 0.2	2.2 ± 0.1	2.1 ± 0.1	-4.7 ± 1.3	-0.3 ± 1.0	0.0 ± 0.0	0.0 ± 0.0	0.0 ± 0.0	0.0 ± 0.0	0.0 ± 0.0
Ry	17.7 ± 1.9	5.9 ± 2.1	5.8 ± 2.1	8.7 ± 2.1	12.9 ± 1.1	19.3 ± 1.7	6.5 ± 2.1	6.5 ± 2.1	8.2 ± 2.4	16.2 ± 1.4
Rz	2.9 ± 1.2	-3.1 ± 1.7	-3.2 ± 1.7	3.1 ± 1.4	1.0 ± 0.7	-1.8 ± 0.8	-7.2 ± 0.9	-7.2 ± 0.9	-30.5 ± 0.9	2.4 ± 0.5

Supplementary Table S14: Transformations of segment frames for REFRAMEd datasets in the curves above.

**Raw data**		RMS	RMSE vs.			
			PiG wand	MA	KAD	MiKneeSoTA
PiG	Flex/Ex	32.59 ± 0.27	2.82 ± 0.06	2.79 ± 0.06	0.56 ± 0.04	3.87 ± 0.76
	Abd/Add	7.21 ± 0.22	6.38 ± 0.13	6.43 ± 0.13	5.09 ± 0.11	3.48 ± 0.36
	Ext/Int	12.60 ± 0.64	5.32 ± 0.13	5.35 ± 0.13	25.31 ± 0.17	8.29 ± 0.74
PiG wand	Flex/Ex	30.44 ± 0.24	-	0.04 ± 0.00	2.65 ± 0.04	4.42 ± 0.86
	Abd/Add	2.28 ± 0.22	-	0.07 ± 0.00	2.13 ± 0.04	6.20 ± 0.44
	Ext/Int	7.89 ± 0.47	-	0.04 ± 0.00	29.58 ± 0.02	4.85 ± 0.55
MA	Flex/Ex	30.47 ± 0.24	-	-	2.62 ± 0.04	4.40 ± 0.86
	Abd/Add	2.28 ± 0.22	-	-	2.16 ± 0.04	6.26 ± 0.44
	Ext/Int	7.86 ± 0.47	-	-	29.62 ± 0.02	4.82 ± 0.55
KAD	Flex/Ex	32.40 ± 0.26	-	-	-	3.73 ± 0.77
	Abd/Add	3.27 ± 0.20	-	-	-	5.51 ± 0.50
	Ext/Int	36.60 ± 0.79	-	-	-	32.84 ± 0.80
MiKneeSoTA	Flex/Ex	31.72 ± 0.26	-	-	-	-
	Abd/Add	6.19 ± 0.23	-	-	-	-
	Ext/Int	5.30 ± 0.45	-	-	-	-

Supplementary Table S15: RMS (RMSE vs. 0) of all datasets and the RMSEs for all combinations for the datasets shown above before REFRAME.

**REFRAMEd**		RMS	RMSE vs.			
			PiG wand	MA	KAD	MiKneeSoTA
PiG	Flex/Ex	33.56 ± 0.27	1.81 ± 0.17	1.81 ± 0.17	1.77 ± 0.18	4.76 ± 0.77
	Abd/Add	2.68 ± 0.22	0.68 ± 0.11	0.67 ± 0.11	0.72 ± 0.12	2.69 ± 0.42
	Ext/Int	2.75 ± 0.64	1.60 ± 0.07	1.60 ± 0.07	1.56 ± 0.07	2.53 ± 0.24
PiG wand	Flex/Ex	32.69 ± 0.24	-	0.01 ± 0.00	0.16 ± 0.02	3.80 ± 0.66
	Abd/Add	2.31 ± 0.22	-	0.01 ± 0.00	0.21 ± 0.06	2.20 ± 0.29
	Ext/Int	2.97 ± 0.47	-	0.02 ± 0.00	0.40 ± 0.05	2.97 ± 0.41
MA	Flex/Ex	32.69 ± 0.24	-	-	0.17 ± 0.02	3.80 ± 0.66
	Abd/Add	2.32 ± 0.22	-	-	0.22 ± 0.06	2.21 ± 0.29
	Ext/Int	2.97 ± 0.47	-	-	0.41 ± 0.05	2.97 ± 0.41
KAD	Flex/Ex	32.69 ± 0.26	-	-	-	3.82 ± 0.67
	Abd/Add	2.22 ± 0.20	-	-	-	2.14 ± 0.30
	Ext/Int	2.86 ± 0.79	-	-	-	2.93 ± 0.45
MiKneeSoTA	Flex/Ex	32.54 ± 0.26	-	-	-	-
	Abd/Add	0.99 ± 0.23	-	-	-	-
	Ext/Int	1.18 ± 0.45	-	-	-	-

Supplementary Table S16: RMS (RMSE vs. 0) of all datasets and the RMSEs for all combinations for the datasets shown above after REFRAME.

3.6 Left Knee of Subject 3

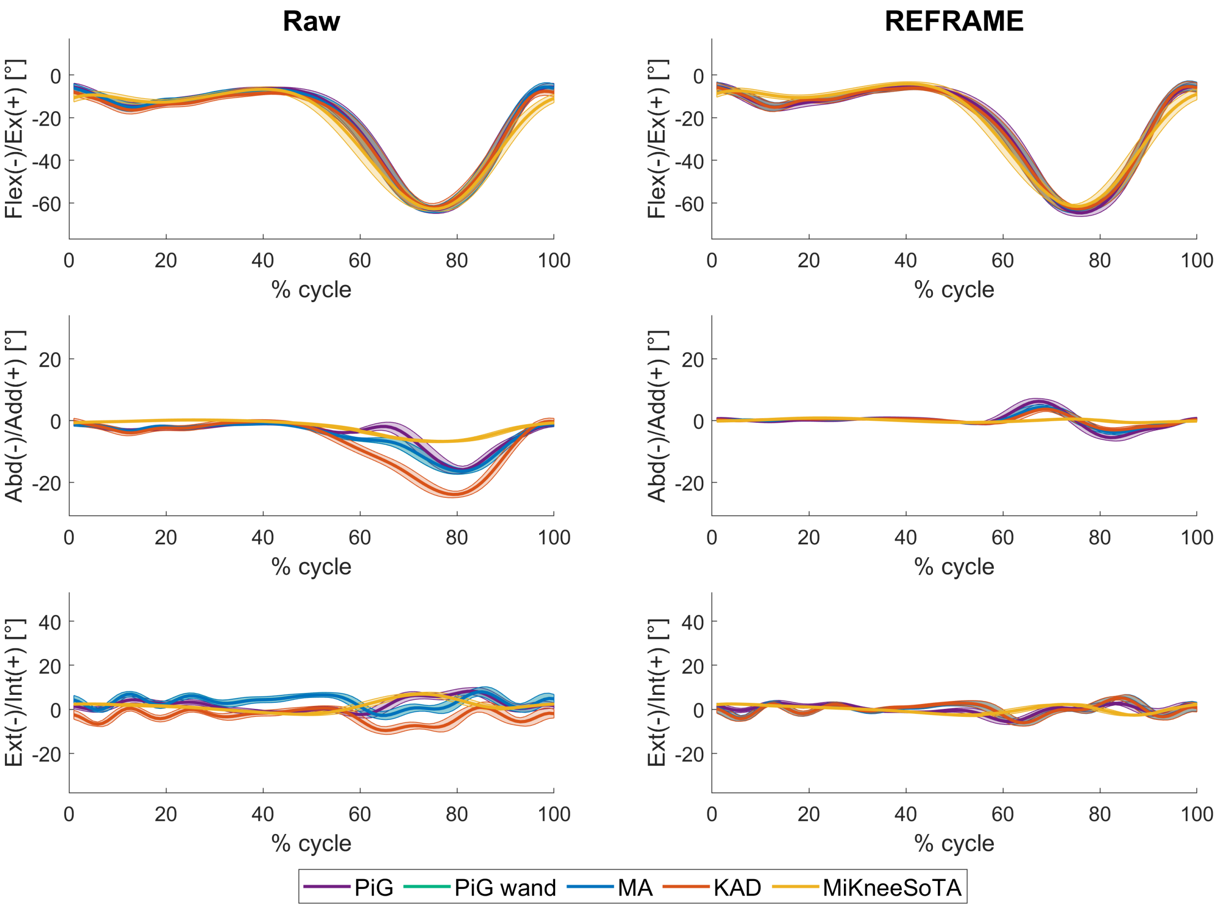

Supplementary Figure S7: Mean kinematics for all 5 markersets with standard deviation over all nine steps for knee 6 (subject 3, left). Left Column: raw kinematics, right column: REFRAMEd kinematics.

	Femur					Tibia				
markerset	PiG	PiG wand	MA	KAD	MiKneeSoTA	PiG	PiG wand	MA	KAD	MiKneeSoTA
Rx	-0.4 ± 0.1	-1.1 ± 0.1	-1.1 ± 0.1	-2.4 ± 0.2	-2.3 ± 0.9	0.0 ± 0.0	0.0 ± 0.0	0.0 ± 0.0	0.0 ± 0.0	0.0 ± 0.0
Ry	10.6 ± 1.5	4.8 ± 1.6	4.8 ± 1.6	8.7 ± 1.6	8.5 ± 1.0	11.8 ± 1.5	4.8 ± 1.6	4.8 ± 1.6	7.0 ± 1.7	8.4 ± 1.1
Rz	5.1 ± 1.5	12.9 ± 1.2	12.9 ± 1.2	22.7 ± 1.3	3.3 ± 0.6	6.2 ± 1.5	9.6 ± 1.2	9.6 ± 1.2	26.2 ± 1.2	4.3 ± 0.4

Supplementary Table S17: Transformations of segment frames for REFRAMEd datasets in the curves above.

**Raw data**		RMS	RMSE vs.			
			PiG wand	MA	KAD	MiKneeSoTA
PiG	Flex/Ex	29.76 ± 0.62	0.61 ± 0.03	0.63 ± 0.03	1.61 ± 0.07	4.72 ± 1.48
	Abd/Add	6.14 ± 0.51	1.69 ± 0.16	1.67 ± 0.16	5.29 ± 0.17	3.65 ± 0.32
	Ext/Int	3.78 ± 0.48	4.06 ± 0.37	4.04 ± 0.37	6.85 ± 0.30	2.84 ± 0.49
PiG wand	Flex/Ex	29.89 ± 0.61	-	0.03 ± 0.00	1.30 ± 0.06	4.55 ± 1.40
	Abd/Add	7.04 ± 0.43	-	0.03 ± 0.00	3.84 ± 0.06	4.04 ± 0.35
	Ext/Int	4.63 ± 0.52	-	0.04 ± 0.00	6.65 ± 0.02	5.34 ± 0.56
MA	Flex/Ex	29.92 ± 0.61	-	-	1.29 ± 0.06	4.54 ± 1.40
	Abd/Add	7.01 ± 0.43	-	-	3.86 ± 0.06	4.03 ± 0.35
	Ext/Int	4.60 ± 0.52	-	-	6.61 ± 0.02	5.32 ± 0.56
KAD	Flex/Ex	29.91 ± 0.60	-	-	-	4.40 ± 1.38
	Abd/Add	10.63 ± 0.46	-	-	-	7.46 ± 0.40
	Ext/Int	4.72 ± 0.52	-	-	-	7.25 ± 0.58
MiKneeSoTA	Flex/Ex	31.38 ± 0.60	-	-	-	-
	Abd/Add	3.30 ± 0.16	-	-	-	-
	Ext/Int	3.01 ± 0.35	-	-	-	-

Supplementary Table S18: RMS (RMSE vs. 0) of all datasets and the RMSEs for all combinations for the datasets shown above before REFRAME.

**REFRAMEd**		RMS	RMSE vs.			
			PiG wand	MA	KAD	MiKneeSoTA
PiG	Flex/Ex	30.17 ± 0.62	1.06 ± 0.07	1.06 ± 0.07	1.07 ± 0.06	4.49 ± 1.54
	Abd/Add	2.58 ± 0.51	0.91 ± 0.08	0.90 ± 0.08	1.24 ± 0.09	2.50 ± 0.37
	Ext/Int	2.28 ± 0.48	2.28 ± 0.16	2.28 ± 0.16	2.35 ± 0.16	2.41 ± 0.30
PiG wand	Flex/Ex	29.94 ± 0.61	-	0.01 ± 0.00	0.17 ± 0.01	4.15 ± 1.43
	Abd/Add	1.77 ± 0.43	-	0.01 ± 0.00	0.38 ± 0.04	1.65 ± 0.36
	Ext/Int	2.95 ± 0.52	-	0.01 ± 0.00	0.19 ± 0.02	3.86 ± 0.44
MA	Flex/Ex	29.94 ± 0.61	-	-	0.17 ± 0.01	4.15 ± 1.43
	Abd/Add	1.78 ± 0.43	-	-	0.38 ± 0.04	1.65 ± 0.36
	Ext/Int	2.95 ± 0.52	-	-	0.19 ± 0.02	3.86 ± 0.44
KAD	Flex/Ex	29.87 ± 0.60	-	-	-	4.13 ± 1.46
	Abd/Add	1.50 ± 0.46	-	-	-	1.37 ± 0.33
	Ext/Int	2.96 ± 0.52	-	-	-	3.90 ± 0.45
MiKneeSoTA	Flex/Ex	30.00 ± 0.60	-	-	-	-
	Abd/Add	0.52 ± 0.16	-	-	-	-
	Ext/Int	1.76 ± 0.35	-	-	-	-

Supplementary Table S19: RMS (RMSE vs. 0) of all datasets and the RMSEs for all combinations for the datasets shown above after REFRAME.

3.7 Right Knee of Subject 4

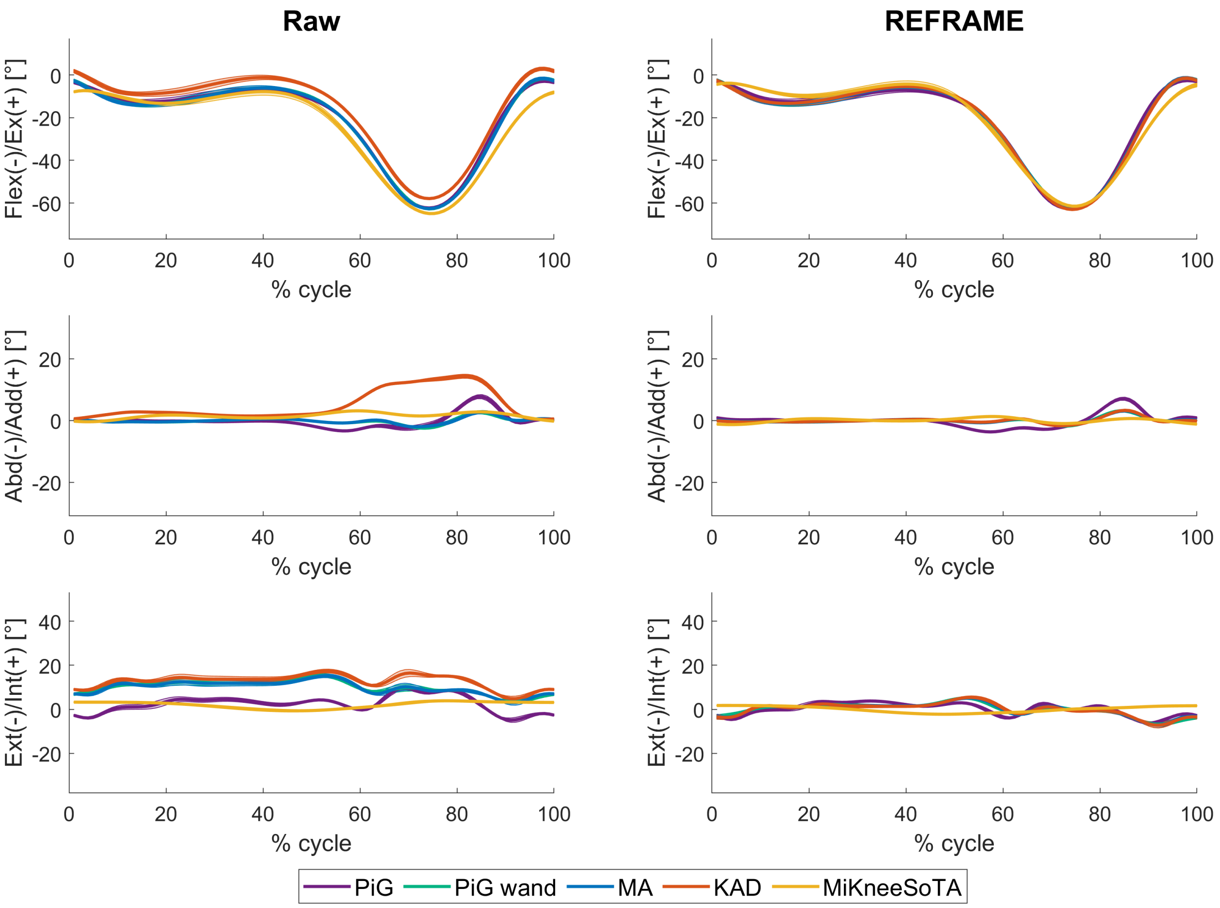

Supplementary Figure S8: Mean kinematics for all 5 markersets with standard deviation over all nine steps for knee 7 (subject 4, right). Left Column: raw kinematics, right column: REFRAMEd kinematics.

	Femur					Tibia				
markerset	PiG	PiG wand	MA	KAD	MiKneeSoTA	PiG	PiG wand	MA	KAD	MiKneeSoTA
Rx	-0.2 ± 0.1	-0.1 ± 0.2	-0.1 ± 0.2	5.2 ± 0.3	-3.5 ± 0.5	0.0 ± 0.0	0.0 ± 0.0	0.0 ± 0.0	0.0 ± 0.0	0.0 ± 0.0
Ry	5.9 ± 0.8	-0.7 ± 1.2	-0.8 ± 1.2	-4.8 ± 1.3	1.0 ± 0.3	7.0 ± 0.7	-1.1 ± 1.2	-1.2 ± 1.2	-4.9 ± 1.3	0.2 ± 0.4
Rz	-4.8 ± 0.6	1.6 ± 0.4	1.5 ± 0.4	-13.7 ± 0.5	-2.5 ± 0.4	-4.6 ± 0.6	-8.8 ± 0.4	-8.8 ± 0.4	-26.4 ± 0.4	-3.9 ± 0.4

Supplementary Table S20: Transformations of segment frames for REFRAMEd datasets in the curves above.

**Raw data**		RMS	RMSE vs.			
			PiG wand	MA	KAD	MiKneeSoTA
PiG	Flex/Ex	28.52 ± 0.24	1.00 ± 0.03	1.04 ± 0.03	4.75 ± 0.07	4.89 ± 0.47
	Abd/Add	2.35 ± 0.16	1.68 ± 0.07	1.70 ± 0.06	6.90 ± 0.14	2.91 ± 0.13
	Ext/Int	4.51 ± 0.28	8.33 ± 0.53	8.23 ± 0.53	10.56 ± 0.50	4.30 ± 0.24
PiG wand	Flex/Ex	28.89 ± 0.23	-	0.18 ± 0.02	4.98 ± 0.03	4.69 ± 0.51
	Abd/Add	1.01 ± 0.12	-	0.15 ± 0.01	7.12 ± 0.05	2.06 ± 0.17
	Ext/Int	10.40 ± 0.53	-	0.43 ± 0.02	3.22 ± 0.02	8.93 ± 0.60
MA	Flex/Ex	28.95 ± 0.23	-	-	5.03 ± 0.03	4.70 ± 0.52
	Abd/Add	0.98 ± 0.12	-	-	7.06 ± 0.05	1.99 ± 0.17
	Ext/Int	10.32 ± 0.53	-	-	3.27 ± 0.02	8.86 ± 0.60
KAD	Flex/Ex	25.33 ± 0.20	-	-	-	8.90 ± 0.44
	Abd/Add	6.93 ± 0.19	-	-	-	5.55 ± 0.23
	Ext/Int	13.11 ± 0.50	-	-	-	11.42 ± 0.57
MiKneeSoTA	Flex/Ex	31.66 ± 0.38	-	-	-	-
	Abd/Add	1.76 ± 0.05	-	-	-	-
	Ext/Int	2.58 ± 0.18	-	-	-	-

Supplementary Table S21: RMS (RMSE vs. 0) of all datasets and the RMSEs for all combinations for the datasets shown above before REFRAME.

**REFRAMEd**		RMS	RMSE vs.			
			PiG wand	MA	KAD	MiKneeSoTA
PiG	Flex/Ex	28.63 ± 0.24	1.12 ± 0.04	1.12 ± 0.03	1.25 ± 0.02	3.63 ± 0.69
	Abd/Add	2.28 ± 0.16	1.60 ± 0.05	1.63 ± 0.05	1.65 ± 0.06	2.45 ± 0.14
	Ext/Int	2.87 ± 0.28	1.76 ± 0.11	1.72 ± 0.12	1.88 ± 0.12	3.72 ± 0.22
PiG wand	Flex/Ex	28.69 ± 0.23	-	0.18 ± 0.02	0.40 ± 0.03	3.36 ± 0.74
	Abd/Add	0.95 ± 0.12	-	0.13 ± 0.01	0.17 ± 0.01	1.02 ± 0.09
	Ext/Int	2.91 ± 0.53	-	0.42 ± 0.02	0.45 ± 0.02	3.91 ± 0.33
MA	Flex/Ex	28.71 ± 0.23	-	-	0.36 ± 0.03	3.42 ± 0.74
	Abd/Add	0.92 ± 0.12	-	-	0.12 ± 0.02	1.00 ± 0.08
	Ext/Int	2.95 ± 0.53	-	-	0.22 ± 0.01	3.93 ± 0.32
KAD	Flex/Ex	28.90 ± 0.20	-	-	-	3.30 ± 0.71
	Abd/Add	0.96 ± 0.19	-	-	-	0.96 ± 0.08
	Ext/Int	3.01 ± 0.50	-	-	-	4.00 ± 0.34
MiKneeSoTA	Flex/Ex	29.05 ± 0.38	-	-	-	-
	Abd/Add	0.70 ± 0.05	-	-	-	-
	Ext/Int	1.40 ± 0.18	-	-	-	-

Supplementary Table S22: RMS (RMSE vs. 0) of all datasets and the RMSEs for all combinations for the datasets shown above after REFRAME.

3.8 Left Knee of Subject 4

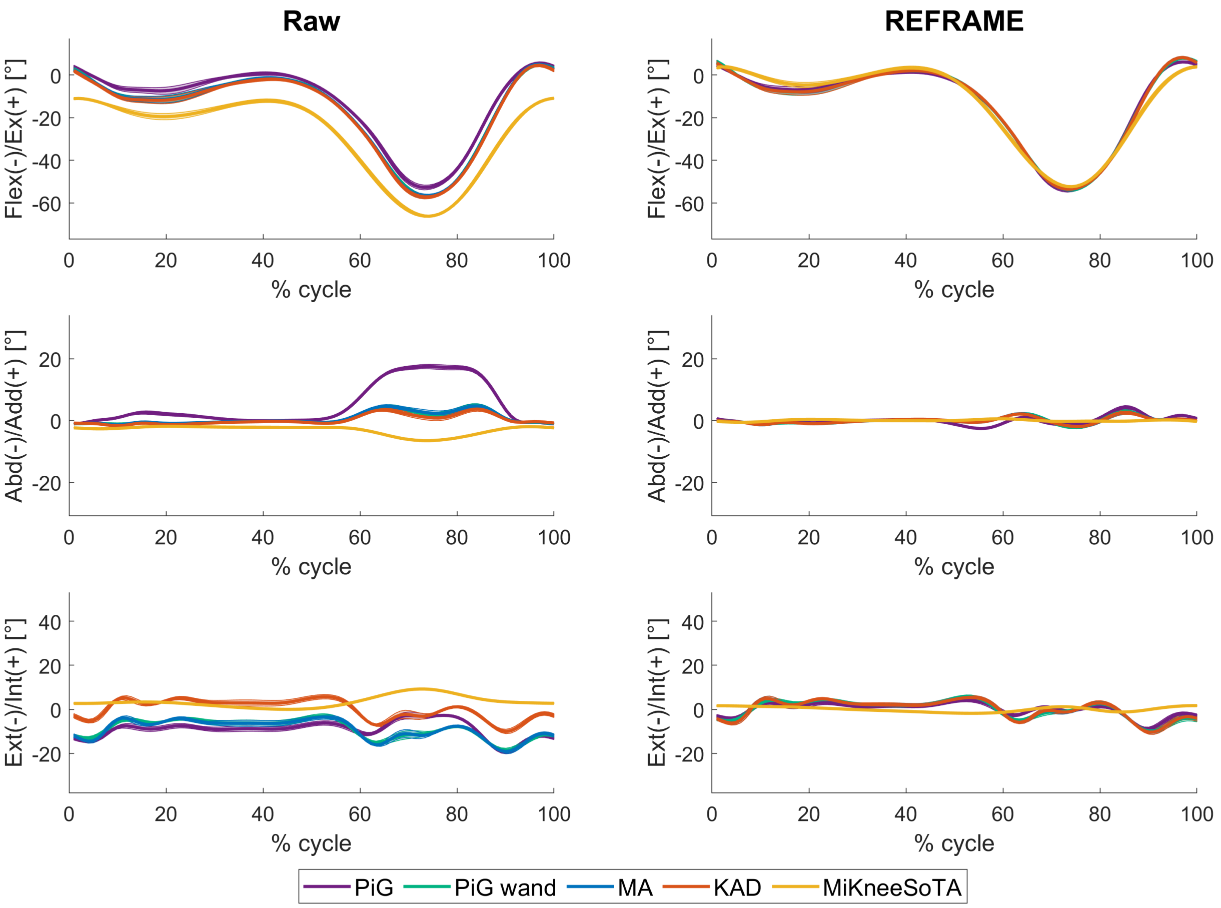

Supplementary Figure S9: Mean kinematics for all 5 markersets with standard deviation over all nine steps for knee 8 (subject 4, left). Left Column: raw kinematics, right column: REFRAMEd kinematics.

	Femur					Tibia				
markerset	PiG	PiG wand	MA	KAD	MiKneeSoTA	PiG	PiG wand	MA	KAD	MiKneeSoTA
Rx	-1.0 ± 0.2	-3.9 ± 0.2	-4.0 ± 0.2	-3.7 ± 0.1	-15.4 ± 0.7	0.0 ± 0.0	0.0 ± 0.0	0.0 ± 0.0	0.0 ± 0.0	0.0 ± 0.0
Ry	-1.2 ± 1.2	-4.4 ± 1.5	-4.4 ± 1.5	-4.4 ± 1.6	8.6 ± 0.5	-1.3 ± 1.1	-3.9 ± 1.5	-4.0 ± 1.5	-3.8 ± 1.5	10.7 ± 0.4
Rz	-22.5 ± 0.5	-3.6 ± 0.2	-3.8 ± 0.2	-2.1 ± 0.2	1.1 ± 0.3	-12.1 ± 0.8	4.7 ± 0.6	4.7 ± 0.6	-2.9 ± 0.6	-0.5 ± 0.3

Supplementary Table S23: Transformations of segment frames for REFRAMEd datasets in the curves above.

Raw data		RMS	RMSE vs.			
			PiG wand	MA	KAD	MiKneeSoTA
PiG	Flex/Ex	22.33 ± 0.42	3.55 ± 0.06	3.62 ± 0.06	4.00 ± 0.06	15.18 ± 0.64
	Abd/Add	8.39 ± 0.19	6.69 ± 0.07	6.62 ± 0.07	7.31 ± 0.08	11.42 ± 0.22
	Ext/Int	9.89 ± 0.25	3.75 ± 0.24	3.76 ± 0.23	9.89 ± 0.32	13.05 ± 0.25
PiG wand	Flex/Ex	25.08 ± 0.38	-	0.26 ± 0.02	0.53 ± 0.01	12.03 ± 0.56
	Abd/Add	2.03 ± 0.17	-	0.14 ± 0.01	0.64 ± 0.01	4.85 ± 0.23
	Ext/Int	9.90 ± 0.21	-	0.55 ± 0.05	8.98 ± 0.00	13.69 ± 0.27
MA	Flex/Ex	25.14 ± 0.38	-	-	0.41 ± 0.00	12.00 ± 0.56
	Abd/Add	2.06 ± 0.18	-	-	0.70 ± 0.01	4.91 ± 0.23
	Ext/Int	10.06 ± 0.21	-	-	9.08 ± 0.01	13.83 ± 0.27
KAD	Flex/Ex	25.42 ± 0.38	-	-	-	11.61 ± 0.55
	Abd/Add	1.57 ± 0.16	-	-	-	4.24 ± 0.24
	Ext/Int	4.40 ± 0.47	-	-	-	6.94 ± 0.46
MiKneeSoTA	Flex/Ex	33.79 ± 0.31	-	-	-	-
	Abd/Add	3.37 ± 0.10	-	-	-	-
	Ext/Int	4.29 ± 0.16	-	-	-	-

Supplementary Table S24: RMS (RMSE vs. 0) of all datasets and the RMSEs for all combinations for the datasets shown above before REFRAME.

REFRAMEd		RMS	RMSE vs.			
			PiG wand	MA	KAD	MiKneeSoTA
PiG	Flex/Ex	23.08 ± 0.42	0.80 ± 0.02	0.81 ± 0.02	0.81 ± 0.02	2.95 ± 0.43
	Abd/Add	1.38 ± 0.19	0.92 ± 0.05	0.91 ± 0.04	0.91 ± 0.05	1.46 ± 0.18
	Ext/Int	3.10 ± 0.25	1.40 ± 0.24	1.49 ± 0.22	1.50 ± 0.23	3.57 ± 0.45
PiG wand	Flex/Ex	23.12 ± 0.38	-	0.25 ± 0.02	0.24 ± 0.02	3.15 ± 0.51
	Abd/Add	1.13 ± 0.17	-	0.14 ± 0.01	0.13 ± 0.01	1.13 ± 0.18
	Ext/Int	4.08 ± 0.21	-	0.54 ± 0.05	0.54 ± 0.05	4.55 ± 0.57
MA	Flex/Ex	23.15 ± 0.38	-	-	0.04 ± 0.00	3.22 ± 0.50
	Abd/Add	1.07 ± 0.18	-	-	0.04 ± 0.01	1.07 ± 0.18
	Ext/Int	4.22 ± 0.21	-	-	0.06 ± 0.01	4.68 ± 0.55
KAD	Flex/Ex	23.13 ± 0.38	-	-	-	3.23 ± 0.50
	Abd/Add	1.06 ± 0.16	-	-	-	1.05 ± 0.18
	Ext/Int	4.23 ± 0.47	-	-	-	4.69 ± 0.55
MiKneeSoTA	Flex/Ex	23.17 ± 0.31	-	-	-	-
	Abd/Add	0.30 ± 0.10	-	-	-	-
	Ext/Int	1.14 ± 0.16	-	-	-	-

Supplementary Table S25: RMS (RMSE vs. 0) of all datasets and the RMSEs for all combinations for the datasets shown above after REFRAME.

3.9 Right Knee of Subject 5

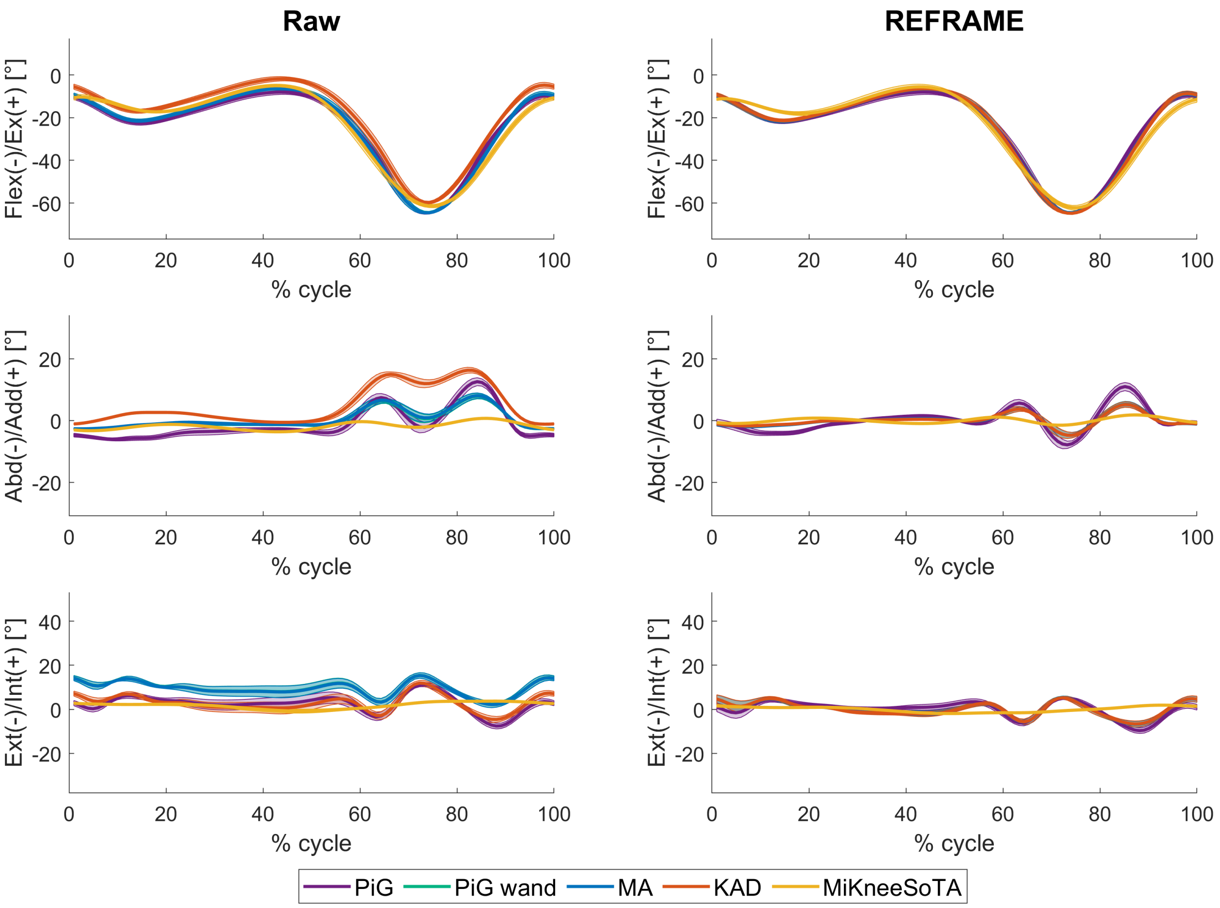

Supplementary Figure S10: Mean kinematics for all 5 markersets with standard deviation over all nine steps for knee 9 (subject 5, right). Left Column: raw kinematics, right column: REFRAMEd kinematics.

	Femur					Tibia				
markerset	PiG	PiG wand	MA	KAD	MiKneeSoTA	PiG	PiG wand	MA	KAD	MiKneeSoTA
Rx	-0.6 ± 0.1	0.6 ± 0.5	0.5 ± 0.5	4.4 ± 0.3	0.6 ± 1.0	0.0 ± 0.0	0.0 ± 0.0	0.0 ± 0.0	0.0 ± 0.0	0.0 ± 0.0
Ry	-2.5 ± 2.3	-4.1 ± 2.0	-4.2 ± 2.0	-7.3 ± 2.1	1.2 ± 0.7	3.5 ± 1.8	-1.6 ± 2.0	-1.7 ± 2.0	-4.4 ± 2.0	4.2 ± 0.9
Rz	-11.3 ± 1.3	-6.2 ± 1.5	-6.4 ± 1.5	-17.2 ± 1.6	-3.4 ± 0.7	-13.4 ± 1.4	-16.5 ± 0.8	-16.6 ± 0.8	-20.7 ± 0.7	-4.3 ± 0.4

Supplementary Table S26: Transformations of segment frames for REFRAMEd datasets in the curves above.

**Raw data**		RMS	RMSE vs.			
			PiG wand	MA	KAD	MiKneeSoTA
PiG	Flex/Ex	30.25 ± 0.68	1.27 ± 0.08	1.25 ± 0.08	5.32 ± 0.08	4.07 ± 0.69
	Abd/Add	5.23 ± 0.47	2.69 ± 0.24	2.73 ± 0.25	6.70 ± 0.34	4.40 ± 0.39
	Ext/Int	4.97 ± 0.48	7.29 ± 0.36	7.18 ± 0.36	1.96 ± 0.16	4.72 ± 0.42
PiG wand	Flex/Ex	30.34 ± 0.66	-	0.06 ± 0.01	4.89 ± 0.06	3.42 ± 0.63
	Abd/Add	3.14 ± 0.29	-	0.13 ± 0.02	5.46 ± 0.12	3.25 ± 0.36
	Ext/Int	10.14 ± 0.89	-	0.11 ± 0.01	6.70 ± 0.03	8.57 ± 0.87
MA	Flex/Ex	30.39 ± 0.66	-	-	4.95 ± 0.06	3.42 ± 0.64
	Abd/Add	3.20 ± 0.29	-	-	5.33 ± 0.11	3.36 ± 0.36
	Ext/Int	10.04 ± 0.89	-	-	6.59 ± 0.04	8.46 ± 0.87
KAD	Flex/Ex	26.35 ± 0.64	-	-	-	5.83 ± 0.61
	Abd/Add	7.59 ± 0.36	-	-	-	8.37 ± 0.38
	Ext/Int	4.94 ± 0.50	-	-	-	4.20 ± 0.41
MiKneeSoTA	Flex/Ex	30.15 ± 0.63	-	-	-	-
	Abd/Add	2.19 ± 0.12	-	-	-	-
	Ext/Int	2.40 ± 0.23	-	-	-	-

Supplementary Table S27: RMS (RMSE vs. 0) of all datasets and the RMSEs for all combinations for the datasets shown above before REFRAME.

**REFRAMEd**		RMS	RMSE vs.			
			PiG wand	MA	KAD	MiKneeSoTA
PiG	Flex/Ex	30.44 ± 0.68	1.01 ± 0.08	1.03 ± 0.08	1.21 ± 0.09	3.61 ± 0.45
	Abd/Add	3.97 ± 0.47	2.00 ± 0.18	2.01 ± 0.18	2.07 ± 0.19	3.59 ± 0.43
	Ext/Int	3.71 ± 0.48	1.88 ± 0.24	1.88 ± 0.25	1.98 ± 0.25	4.15 ± 0.41
PiG wand	Flex/Ex	30.77 ± 0.66	-	0.02 ± 0.00	0.26 ± 0.02	3.15 ± 0.39
	Abd/Add	2.13 ± 0.29	-	0.02 ± 0.00	0.12 ± 0.01	1.76 ± 0.25
	Ext/Int	3.37 ± 0.89	-	0.03 ± 0.00	0.14 ± 0.01	3.49 ± 0.25
MA	Flex/Ex	30.78 ± 0.66	-	-	0.25 ± 0.02	3.15 ± 0.39
	Abd/Add	2.13 ± 0.29	-	-	0.11 ± 0.01	1.76 ± 0.25
	Ext/Int	3.36 ± 0.89	-	-	0.14 ± 0.02	3.48 ± 0.25
KAD	Flex/Ex	30.90 ± 0.64	-	-	-	3.12 ± 0.38
	Abd/Add	2.09 ± 0.36	-	-	-	1.71 ± 0.25
	Ext/Int	3.38 ± 0.50	-	-	-	3.47 ± 0.25
MiKneeSoTA	Flex/Ex	30.78 ± 0.63	-	-	-	-
	Abd/Add	0.88 ± 0.12	-	-	-	-
	Ext/Int	1.28 ± 0.23	-	-	-	-

Supplementary Table S28: RMS (RMSE vs. 0) of all datasets and the RMSEs for all combinations for the datasets shown above after REFRAME.

3.10 Left Knee of Subject 5

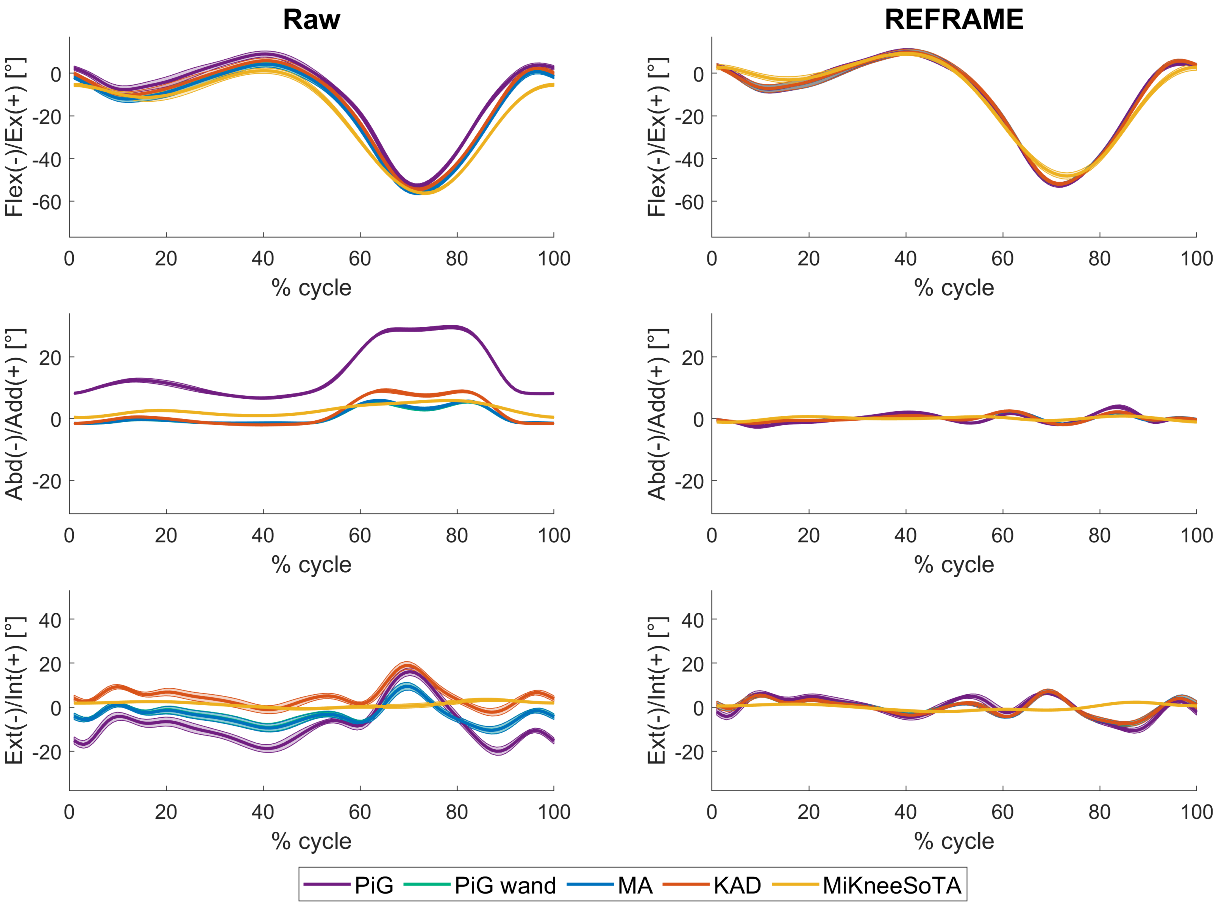

Supplementary Figure S11: Mean kinematics for all 5 markersets with standard deviation over all nine steps for knee 10 (subject 5, left). Left Column: raw kinematics, right column: REFRAMEd kinematics.

	Femur					Tibia				
markerset	PiG	PiG wand	MA	KAD	MiKneeSoTA	PiG	PiG wand	MA	KAD	MiKneeSoTA
Rx	0.4 ± 0.5	-4.7 ± 0.3	-4.7 ± 0.3	-3.8 ± 0.1	-8.1 ± 1.0	0.0 ± 0.0	0.0 ± 0.0	0.0 ± 0.0	0.0 ± 0.0	0.0 ± 0.0
Ry	13.4 ± 2.0	5.2 ± 2.0	5.3 ± 2.0	4.8 ± 2.1	-1.3 ± 1.1	3.1 ± 2.0	5.9 ± 1.9	6.0 ± 1.9	5.2 ± 2.0	-3.0 ± 1.1
Rz	-32.4 ± 0.5	-9.5 ± 0.4	-9.7 ± 0.4	-14.7 ± 0.4	-5.0 ± 0.9	-19.8 ± 0.9	-4.5 ± 0.9	-4.5 ± 0.9	-17.8 ± 1.0	-6.2 ± 0.8

Supplementary Table S29: Transformations of segment frames for REFRAMEd datasets in the curves above.

**Raw data**		RMS	RMSE vs.			
			PiG wand	MA	KAD	MiKneeSoTA
PiG	Flex/Ex	21.38 ± 0.48	5.28 ± 0.10	5.34 ± 0.11	3.60 ± 0.10	9.06 ± 0.83
	Abd/Add	17.01 ± 0.19	15.42 ± 0.16	15.35 ± 0.16	13.75 ± 0.13	13.85 ± 0.19
	Ext/Int	12.17 ± 0.65	7.13 ± 0.53	7.00 ± 0.53	13.92 ± 0.48	13.31 ± 0.69
PiG wand	Flex/Ex	24.62 ± 0.37	-	0.08 ± 0.01	1.75 ± 0.01	4.58 ± 0.72
	Abd/Add	2.63 ± 0.17	-	0.08 ± 0.01	2.02 ± 0.03	2.43 ± 0.13
	Ext/Int	5.76 ± 0.70	-	0.20 ± 0.02	8.31 ± 0.01	6.81 ± 0.74
MA	Flex/Ex	24.65 ± 0.37	-	-	1.81 ± 0.01	4.54 ± 0.71
	Abd/Add	2.69 ± 0.17	-	-	1.94 ± 0.03	2.40 ± 0.13
	Ext/Int	5.88 ± 0.72	-	-	8.48 ± 0.02	6.93 ± 0.76
KAD	Flex/Ex	23.42 ± 0.37	-	-	-	5.89 ± 0.75
	Abd/Add	4.47 ± 0.18	-	-	-	2.70 ± 0.21
	Ext/Int	6.92 ± 0.66	-	-	-	6.25 ± 0.72
MiKneeSoTA	Flex/Ex	26.41 ± 0.30	-	-	-	-
	Abd/Add	3.23 ± 0.14	-	-	-	-
	Ext/Int	1.89 ± 0.25	-	-	-	-

Supplementary Table S30: RMS (RMSE vs. 0) of all datasets and the RMSEs for all combinations for the datasets shown above before REFRAME.

**REFRAMEd**		RMS	RMSE vs.			
			PiG wand	MA	KAD	MiKneeSoTA
PiG	Flex/Ex	22.01 ± 0.48	0.63 ± 0.09	0.62 ± 0.09	0.66 ± 0.09	3.56 ± 0.86
	Abd/Add	1.57 ± 0.19	0.87 ± 0.08	0.89 ± 0.08	0.84 ± 0.07	1.46 ± 0.14
	Ext/Int	4.69 ± 0.65	2.00 ± 0.21	2.04 ± 0.22	1.98 ± 0.21	5.17 ± 0.40
PiG wand	Flex/Ex	21.93 ± 0.37	-	0.02 ± 0.00	0.08 ± 0.01	3.47 ± 0.87
	Abd/Add	1.05 ± 0.17	-	0.02 ± 0.00	0.08 ± 0.03	0.99 ± 0.07
	Ext/Int	3.75 ± 0.70	-	0.08 ± 0.01	0.11 ± 0.01	4.08 ± 0.46
MA	Flex/Ex	21.93 ± 0.37	-	-	0.10 ± 0.01	3.47 ± 0.87
	Abd/Add	1.05 ± 0.17	-	-	0.10 ± 0.03	0.99 ± 0.07
	Ext/Int	3.74 ± 0.72	-	-	0.11 ± 0.01	4.05 ± 0.45
KAD	Flex/Ex	21.97 ± 0.37	-	-	-	3.43 ± 0.87
	Abd/Add	1.11 ± 0.18	-	-	-	1.03 ± 0.07
	Ext/Int	3.73 ± 0.66	-	-	-	4.05 ± 0.45
MiKneeSoTA	Flex/Ex	21.45 ± 0.30	-	-	-	-
	Abd/Add	0.56 ± 0.14	-	-	-	-
	Ext/Int	1.31 ± 0.25	-	-	-	-

Supplementary Table S31: RMS (RMSE vs. 0) of all datasets and the RMSEs for all combinations for the datasets shown above after REFRAME.

3.11 Right Knee of Subject 6

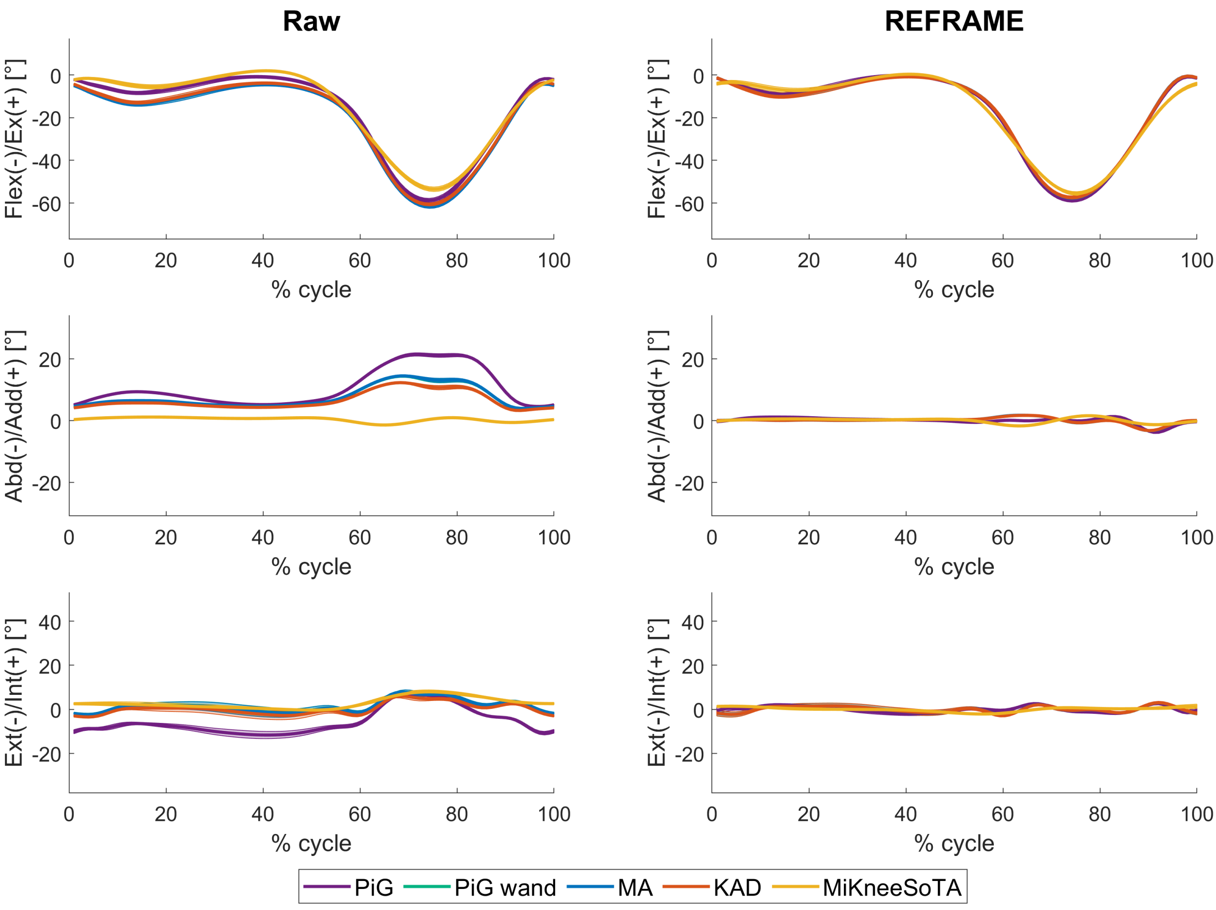

Supplementary Figure S12: Mean kinematics for all 5 markersets with standard deviation over all nine steps for knee 11 (subject 6, right). Left Column: raw kinematics, right column: REFRAMEd kinematics.

	Femur					Tibia				
markerset	PiG	PiG wand	MA	KAD	MiKneeSoTA	PiG	PiG wand	MA	KAD	MiKneeSoTA
Rx	-0.2 ± 0.2	-3.8 ± 0.1	-3.8 ± 0.1	-3.1 ± 0.1	1.6 ± 0.7	0.0 ± 0.0	0.0 ± 0.0	0.0 ± 0.0	0.0 ± 0.0	0.0 ± 0.0
Ry	7.2 ± 1.1	2.0 ± 1.5	2.0 ± 1.5	2.2 ± 1.5	6.7 ± 0.5	2.4 ± 1.1	-2.3 ± 1.4	-2.3 ± 1.4	-1.7 ± 1.4	6.4 ± 0.4
Rz	-23.3 ± 0.6	-12.3 ± 0.6	-12.3 ± 0.6	-10.1 ± 0.6	-2.1 ± 0.2	-13.5 ± 0.7	-12.1 ± 0.7	-12.1 ± 0.7	-8.8 ± 0.6	-2.9 ± 0.6

Supplementary Table S32: Transformations of segment frames for REFRAMEd datasets in the curves above.

**Raw data**		RMS	RMSE vs.			
			PiG wand	MA	KAD	MiKneeSoTA
PiG	Flex/Ex	26.05 ± 0.46	3.80 ± 0.07	3.82 ± 0.07	3.19 ± 0.07	3.07 ± 0.35
	Abd/Add	11.99 ± 0.19	3.99 ± 0.04	3.95 ± 0.04	5.16 ± 0.05	11.86 ± 0.19
	Ext/Int	8.08 ± 0.61	7.97 ± 0.24	7.91 ± 0.24	6.91 ± 0.24	9.23 ± 0.84
PiG wand	Flex/Ex	28.48 ± 0.43	-	0.02 ± 0.00	0.64 ± 0.01	6.11 ± 0.51
	Abd/Add	8.20 ± 0.16	-	0.04 ± 0.00	1.19 ± 0.01	8.06 ± 0.17
	Ext/Int	3.42 ± 0.26	-	0.06 ± 0.00	1.36 ± 0.01	2.48 ± 0.49
MA	Flex/Ex	28.49 ± 0.43	-	-	0.66 ± 0.01	6.13 ± 0.51
	Abd/Add	8.24 ± 0.17	-	-	1.23 ± 0.01	8.10 ± 0.17
	Ext/Int	3.40 ± 0.27	-	-	1.30 ± 0.01	2.50 ± 0.51
KAD	Flex/Ex	27.99 ± 0.43	-	-	-	5.55 ± 0.50
	Abd/Add	7.05 ± 0.15	-	-	-	6.90 ± 0.16
	Ext/Int	2.84 ± 0.38	-	-	-	3.32 ± 0.67
MiKneeSoTA	Flex/Ex	24.27 ± 0.49	-	-	-	-
	Abd/Add	0.81 ± 0.05	-	-	-	-
	Ext/Int	3.80 ± 0.51	-	-	-	-

Supplementary Table S33: RMS (RMSE vs. 0) of all datasets and the RMSEs for all combinations for the datasets shown above before REFRAME.

**REFRAMEd**		RMS	RMSE vs.			
			PiG wand	MA	KAD	MiKneeSoTA
PiG	Flex/Ex	26.25 ± 0.46	0.77 ± 0.06	0.77 ± 0.06	0.81 ± 0.06	2.55 ± 0.27
	Abd/Add	1.07 ± 0.19	0.86 ± 0.06	0.86 ± 0.06	0.85 ± 0.06	0.90 ± 0.10
	Ext/Int	1.48 ± 0.61	1.17 ± 0.18	1.17 ± 0.18	1.19 ± 0.18	1.71 ± 0.32
PiG wand	Flex/Ex	25.88 ± 0.43	-	0.01 ± 0.00	0.06 ± 0.01	2.67 ± 0.30
	Abd/Add	1.02 ± 0.16	-	0.01 ± 0.00	0.04 ± 0.00	1.29 ± 0.08
	Ext/Int	1.68 ± 0.26	-	0.02 ± 0.00	0.04 ± 0.00	1.93 ± 0.22
MA	Flex/Ex	25.88 ± 0.43	-	-	0.07 ± 0.01	2.67 ± 0.30
	Abd/Add	1.02 ± 0.17	-	-	0.04 ± 0.00	1.30 ± 0.08
	Ext/Int	1.67 ± 0.27	-	-	0.04 ± 0.01	1.92 ± 0.22
KAD	Flex/Ex	25.83 ± 0.43	-	-	-	2.66 ± 0.30
	Abd/Add	1.00 ± 0.15	-	-	-	1.28 ± 0.08
	Ext/Int	1.67 ± 0.38	-	-	-	1.91 ± 0.22
MiKneeSoTA	Flex/Ex	25.54 ± 0.49	-	-	-	-
	Abd/Add	0.80 ± 0.05	-	-	-	-
	Ext/Int	1.05 ± 0.51	-	-	-	-

Supplementary Table S34: RMS (RMSE vs. 0) of all datasets and the RMSEs for all combinations for the datasets shown above after REFRAME.

3.12 Left Knee of Subject 6

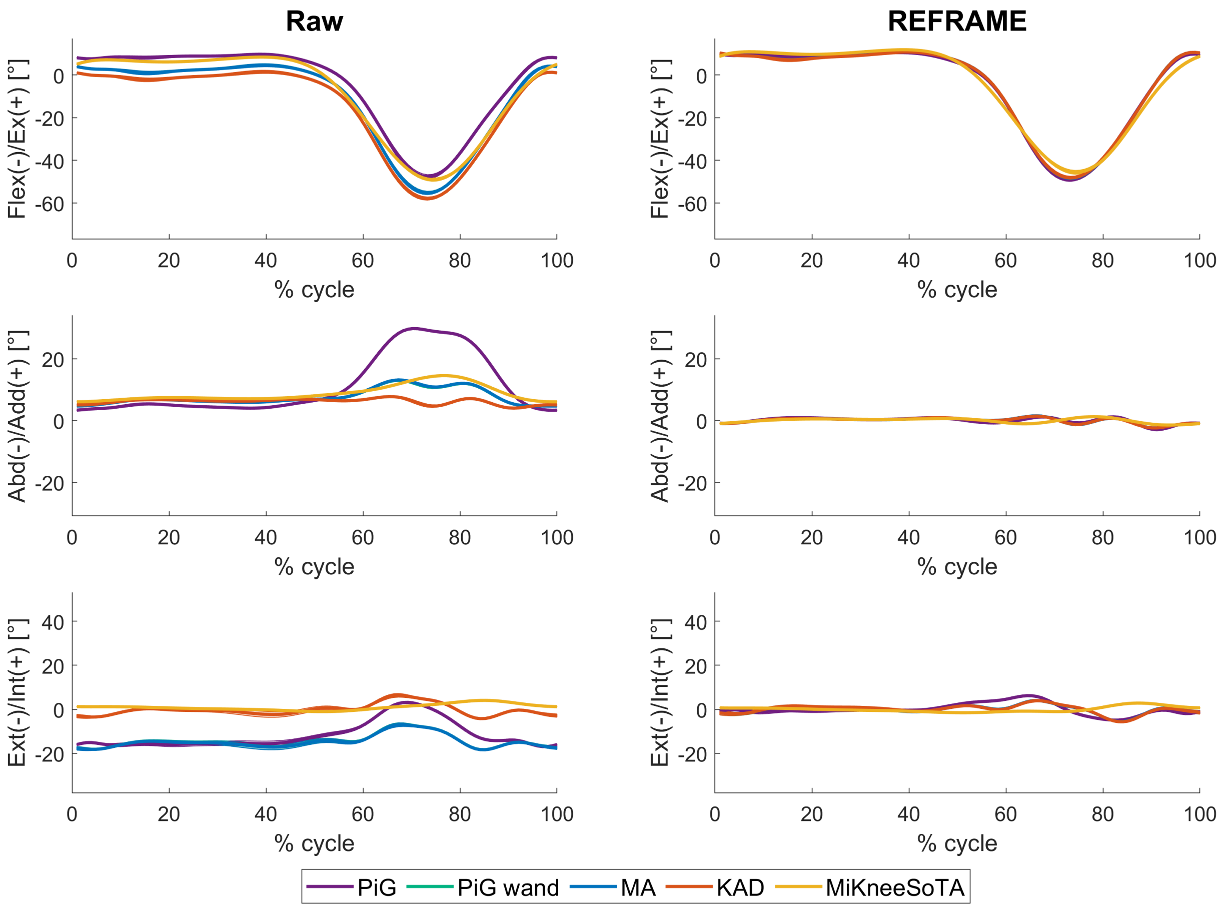

Supplementary Figure S13: Mean kinematics for all 5 markersets with standard deviation over all nine steps for knee 12 (subject 6, left). Left Column: raw kinematics, right column: REFRAMEd kinematics.

	Femur					Tibia				
markerset	PiG	PiG wand	MA	KAD	MiKneeSoTA	PiG	PiG wand	MA	KAD	MiKneeSoTA
Rx	-0.1 ± 0.3	-7.1 ± 0.2	-7.1 ± 0.2	-9.4 ± 0.1	-3.4 ± 0.6	0.0 ± 0.0	0.0 ± 0.0	0.0 ± 0.0	0.0 ± 0.0	0.0 ± 0.0
Ry	8.1 ± 0.9	4.3 ± 0.8	4.3 ± 0.8	4.4 ± 0.8	0.2 ± 0.5	-1.1 ± 0.8	-3.0 ± 0.8	-3.1 ± 0.8	-1.9 ± 0.7	-8.0 ± 0.5
Rz	-31.1 ± 0.5	-8.3 ± 0.3	-8.4 ± 0.3	-1.4 ± 0.3	-7.5 ± 0.3	-16.9 ± 0.6	7.1 ± 0.4	7.0 ± 0.4	-0.7 ± 0.4	-8.0 ± 0.3

Supplementary Table S35: Transformations of segment frames for REFRAMEd datasets in the curves above.

**Raw data**		RMS	RMSE vs.			
			PiG wand	MA	KAD	MiKneeSoTA
PiG	Flex/Ex	19.86 ± 0.35	6.54 ± 0.05	6.55 ± 0.05	9.55 ± 0.05	4.48 ± 0.51
	Abd/Add	14.49 ± 0.12	7.84 ± 0.07	7.80 ± 0.07	10.53 ± 0.10	7.31 ± 0.08
	Ext/Int	13.19 ± 0.49	4.48 ± 0.09	4.52 ± 0.10	12.17 ± 0.24	14.06 ± 0.43
PiG wand	Flex/Ex	23.33 ± 0.32	-	0.02 ± 0.00	3.07 ± 0.01	3.93 ± 0.39
	Abd/Add	7.98 ± 0.08	-	0.05 ± 0.00	2.70 ± 0.03	1.52 ± 0.10
	Ext/Int	14.90 ± 0.43	-	0.07 ± 0.01	14.34 ± 0.01	15.95 ± 0.38
MA	Flex/Ex	23.35 ± 0.32	-	-	3.05 ± 0.01	3.94 ± 0.39
	Abd/Add	8.01 ± 0.08	-	-	2.75 ± 0.03	1.51 ± 0.10
	Ext/Int	14.97 ± 0.43	-	-	14.40 ± 0.01	16.01 ± 0.38
KAD	Flex/Ex	24.93 ± 0.31	-	-	-	6.48 ± 0.49
	Abd/Add	6.21 ± 0.06	-	-	-	3.68 ± 0.13
	Ext/Int	2.66 ± 0.23	-	-	-	3.34 ± 0.28
MiKneeSoTA	Flex/Ex	21.84 ± 0.29	-	-	-	-
	Abd/Add	9.09 ± 0.07	-	-	-	-
	Ext/Int	1.74 ± 0.12	-	-	-	-

Supplementary Table S36: RMS (RMSE vs. 0) of all datasets and the RMSEs for all combinations for the datasets shown above before REFRAME.

**REFRAMEd**		RMS	RMSE vs.			
			PiG wand	MA	KAD	MiKneeSoTA
PiG	Flex/Ex	21.11 ± 0.35	0.77 ± 0.04	0.76 ± 0.04	0.81 ± 0.04	2.42 ± 0.27
	Abd/Add	0.97 ± 0.12	0.40 ± 0.03	0.40 ± 0.03	0.38 ± 0.03	0.77 ± 0.09
	Ext/Int	2.66 ± 0.49	1.78 ± 0.07	1.79 ± 0.07	1.84 ± 0.07	3.54 ± 0.25
PiG wand	Flex/Ex	20.86 ± 0.32	-	0.01 ± 0.00	0.09 ± 0.00	2.59 ± 0.23
	Abd/Add	0.89 ± 0.08	-	0.01 ± 0.00	0.03 ± 0.00	0.87 ± 0.10
	Ext/Int	2.03 ± 0.43	-	0.02 ± 0.00	0.09 ± 0.00	2.90 ± 0.27
MA	Flex/Ex	20.87 ± 0.32	-	-	0.10 ± 0.01	2.59 ± 0.23
	Abd/Add	0.90 ± 0.08	-	-	0.03 ± 0.00	0.88 ± 0.10
	Ext/Int	2.02 ± 0.43	-	-	0.10 ± 0.00	2.89 ± 0.27
KAD	Flex/Ex	20.78 ± 0.31	-	-	-	2.59 ± 0.23
	Abd/Add	0.89 ± 0.06	-	-	-	0.85 ± 0.10
	Ext/Int	2.04 ± 0.23	-	-	-	2.93 ± 0.27
MiKneeSoTA	Flex/Ex	20.71 ± 0.29	-	-	-	-
	Abd/Add	0.73 ± 0.07	-	-	-	-
	Ext/Int	1.19 ± 0.12	-	-	-	-

Supplementary Table S37: RMS (RMSE vs. 0) of all datasets and the RMSEs for all combinations for the datasets shown above after REFRAME.

3.13 Right Knee of Subject 7

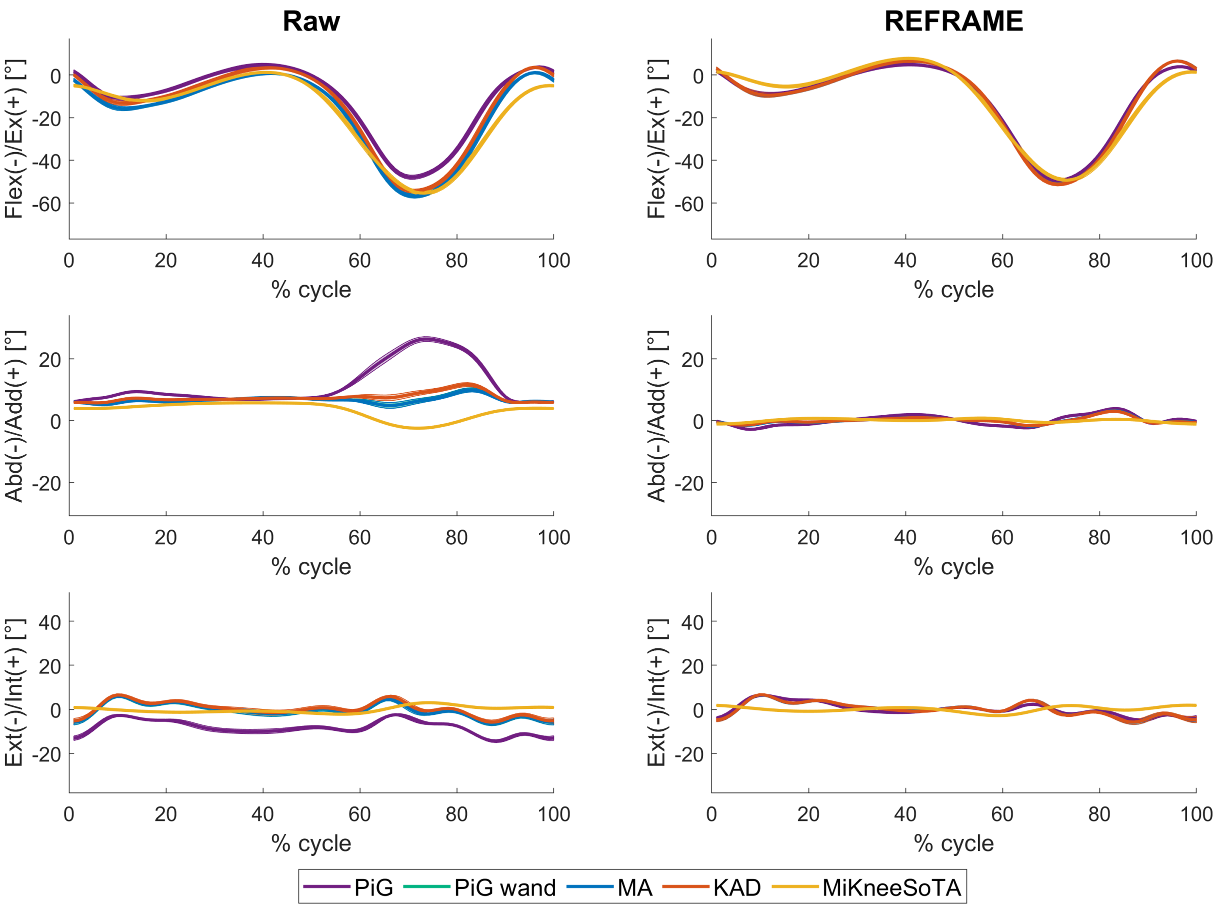

Supplementary Figure S14: Mean kinematics for all 5 markersets with standard deviation over all nine steps for knee 13 (subject 7, right). Left Column: raw kinematics, right column: REFRAMEd kinematics.

	Femur					Tibia				
markerset	PiG	PiG wand	MA	KAD	MiKneeSoTA	PiG	PiG wand	MA	KAD	MiKneeSoTA
Rx	-2.2 ± 0.1	-5.8 ± 0.1	-5.8 ± 0.1	-3.3 ± 0.1	-6.6 ± 0.3	0.0 ± 0.0	0.0 ± 0.0	0.0 ± 0.0	0.0 ± 0.0	0.0 ± 0.0
Ry	-2.7 ± 0.6	1.6 ± 0.7	1.6 ± 0.7	1.4 ± 0.7	5.7 ± 0.4	-10.0 ± 0.6	-5.1 ± 0.7	-5.1 ± 0.7	-5.4 ± 0.7	0.8 ± 0.4
Rz	-22.8 ± 0.8	-0.8 ± 0.9	-0.7 ± 0.9	-3.8 ± 0.9	6.5 ± 0.3	-13.8 ± 0.7	0.2 ± 0.6	0.2 ± 0.6	-3.4 ± 0.6	7.3 ± 0.5

Supplementary Table S38: Transformations of segment frames for REFRAMEd datasets in the curves above.

**Raw data**		RMS	RMSE vs.			
			PiG wand	MA	KAD	MiKneeSoTA
PiG	Flex/Ex	20.35 ± 0.36	6.19 ± 0.10	6.17 ± 0.10	3.95 ± 0.10	7.72 ± 0.38
	Abd/Add	13.41 ± 0.34	8.17 ± 0.14	8.20 ± 0.14	7.00 ± 0.13	12.43 ± 0.37
	Ext/Int	8.87 ± 0.37	7.72 ± 0.15	7.79 ± 0.15	8.47 ± 0.15	8.85 ± 0.42
PiG wand	Flex/Ex	25.55 ± 0.34	-	0.01 ± 0.00	2.51 ± 0.01	3.79 ± 0.34
	Abd/Add	6.76 ± 0.24	-	0.04 ± 0.00	1.19 ± 0.01	4.46 ± 0.34
	Ext/Int	3.25 ± 0.23	-	0.07 ± 0.00	0.84 ± 0.01	3.90 ± 0.31
MA	Flex/Ex	25.54 ± 0.34	-	-	2.50 ± 0.01	3.79 ± 0.34
	Abd/Add	6.74 ± 0.24	-	-	1.22 ± 0.01	4.42 ± 0.34
	Ext/Int	3.24 ± 0.23	-	-	0.79 ± 0.01	3.90 ± 0.32
KAD	Flex/Ex	23.93 ± 0.35	-	-	-	4.93 ± 0.36
	Abd/Add	7.58 ± 0.25	-	-	-	5.56 ± 0.34
	Ext/Int	3.24 ± 0.23	-	-	-	3.84 ± 0.36
MiKneeSoTA	Flex/Ex	25.75 ± 0.39	-	-	-	-
	Abd/Add	4.18 ± 0.03	-	-	-	-
	Ext/Int	1.43 ± 0.08	-	-	-	-

Supplementary Table S39: RMS (RMSE vs. 0) of all datasets and the RMSEs for all combinations for the datasets shown above before REFRAME.

**REFRAMEd**		RMS	RMSE vs.			
			PiG wand	MA	KAD	MiKneeSoTA
PiG	Flex/Ex	21.05 ± 0.36	1.23 ± 0.03	1.22 ± 0.03	1.25 ± 0.03	3.36 ± 0.41
	Abd/Add	1.68 ± 0.34	0.82 ± 0.06	0.82 ± 0.06	0.88 ± 0.06	1.70 ± 0.15
	Ext/Int	2.89 ± 0.37	0.98 ± 0.07	0.98 ± 0.07	0.96 ± 0.06	3.54 ± 0.20
PiG wand	Flex/Ex	21.91 ± 0.34	-	0.00 ± 0.00	0.04 ± 0.00	3.50 ± 0.32
	Abd/Add	1.08 ± 0.24	-	0.01 ± 0.00	0.09 ± 0.01	1.02 ± 0.15
	Ext/Int	3.06 ± 0.23	-	0.01 ± 0.00	0.05 ± 0.00	3.72 ± 0.28
MA	Flex/Ex	21.91 ± 0.34	-	-	0.04 ± 0.00	3.50 ± 0.32
	Abd/Add	1.09 ± 0.24	-	-	0.09 ± 0.01	1.02 ± 0.15
	Ext/Int	3.06 ± 0.23	-	-	0.05 ± 0.00	3.72 ± 0.28
KAD	Flex/Ex	21.94 ± 0.35	-	-	-	3.49 ± 0.32
	Abd/Add	1.04 ± 0.25	-	-	-	0.96 ± 0.15
	Ext/Int	3.07 ± 0.23	-	-	-	3.74 ± 0.28
MiKneeSoTA	Flex/Ex	21.77 ± 0.39	-	-	-	-
	Abd/Add	0.56 ± 0.03	-	-	-	-
	Ext/Int	1.25 ± 0.08	-	-	-	-

Supplementary Table S40: RMS (RMSE vs. 0) of all datasets and the RMSEs for all combinations for the datasets shown above after REFRAME.

3.14 Left Knee of Subject 7

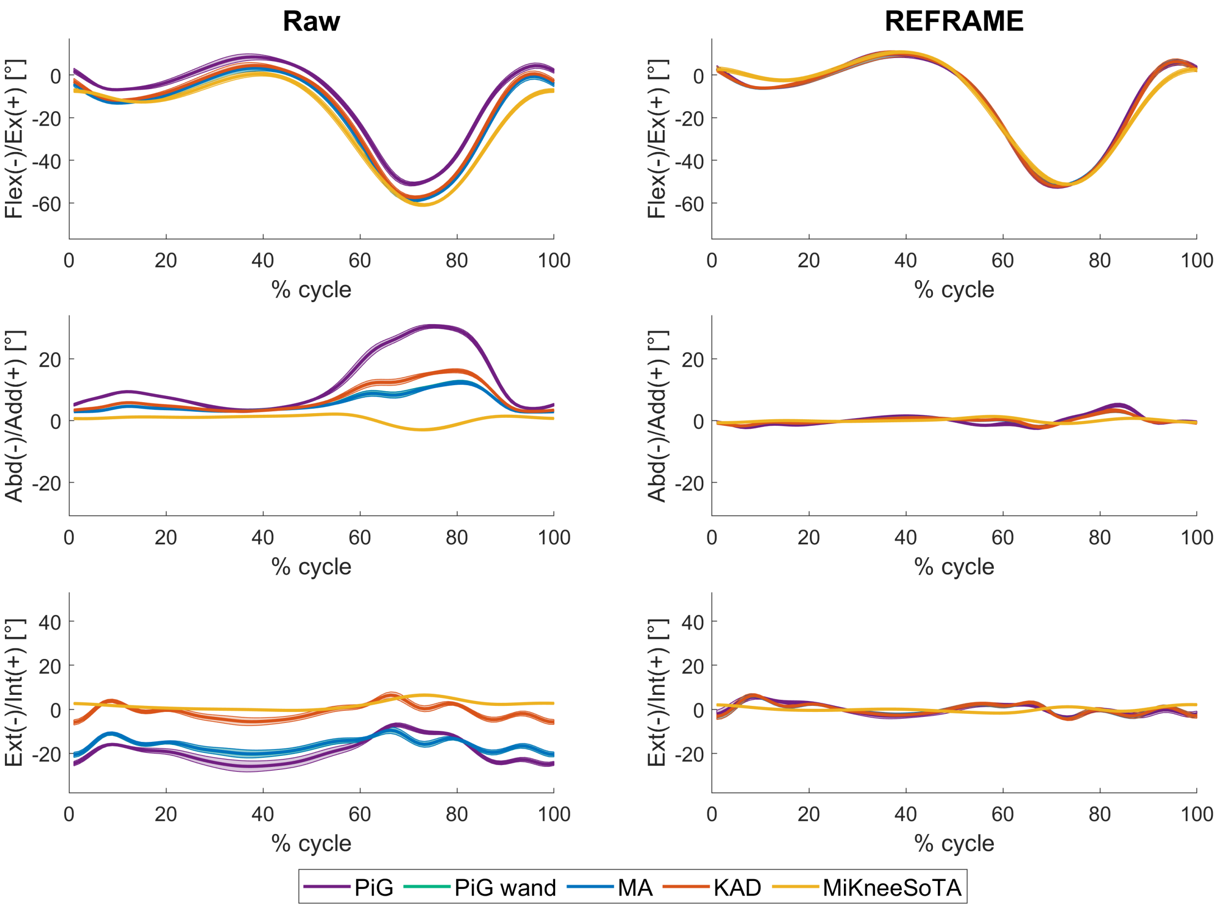

Supplementary Figure S15: Mean kinematics for all 5 markersets with standard deviation over all nine steps for knee 14 (subject 7, left). Left Column: raw kinematics, right column: REFRAMEd kinematics.

	Femur					Tibia				
markerset	PiG	PiG wand	MA	KAD	MiKneeSoTA	PiG	PiG wand	MA	KAD	MiKneeSoTA
Rx	-1.1 ± 0.7	-7.1 ± 0.5	-7.1 ± 0.5	-5.5 ± 0.1	-10.2 ± 0.7	0.0 ± 0.0	0.0 ± 0.0	0.0 ± 0.0	0.0 ± 0.0	0.0 ± 0.0
Ry	5.8 ± 1.6	2.9 ± 1.7	2.8 ± 1.7	2.6 ± 1.8	6.2 ± 0.8	-1.5 ± 1.6	-1.3 ± 1.7	-1.3 ± 1.7	-2.3 ± 1.7	5.0 ± 0.9
Rz	-31.5 ± 0.7	-9.6 ± 0.7	-9.5 ± 0.7	-14.2 ± 0.7	1.3 ± 0.3	-9.3 ± 0.9	7.8 ± 0.4	7.8 ± 0.4	-11.5 ± 0.4	0.4 ± 0.4

Supplementary Table S41: Transformations of segment frames for REFRAMEd datasets in the curves above.

**Raw data**		RMS	RMSE vs.			
			PiG wand	MA	KAD	MiKneeSoTA
PiG	Flex/Ex	21.68 ± 0.35	6.96 ± 0.09	6.94 ± 0.09	5.62 ± 0.08	10.88 ± 0.63
	Abd/Add	15.33 ± 0.29	9.20 ± 0.09	9.24 ± 0.09	7.12 ± 0.08	15.64 ± 0.39
	Ext/Int	20.03 ± 0.94	4.44 ± 0.27	4.49 ± 0.28	18.23 ± 0.37	21.57 ± 0.92
PiG wand	Flex/Ex	26.64 ± 0.33	-	0.02 ± 0.00	1.36 ± 0.01	4.73 ± 0.47
	Abd/Add	6.38 ± 0.23	-	0.04 ± 0.00	2.09 ± 0.02	6.51 ± 0.36
	Ext/Int	16.56 ± 0.58	-	0.07 ± 0.00	15.05 ± 0.01	18.39 ± 0.58
MA	Flex/Ex	26.63 ± 0.33	-	-	1.34 ± 0.01	4.75 ± 0.47
	Abd/Add	6.34 ± 0.23	-	-	2.13 ± 0.02	6.47 ± 0.36
	Ext/Int	16.50 ± 0.58	-	-	14.99 ± 0.01	18.33 ± 0.58
KAD	Flex/Ex	25.72 ± 0.33	-	-	-	5.79 ± 0.52
	Abd/Add	8.36 ± 0.24	-	-	-	8.55 ± 0.36
	Ext/Int	3.64 ± 0.52	-	-	-	4.32 ± 0.47
MiKneeSoTA	Flex/Ex	28.94 ± 0.38	-	-	-	-
	Abd/Add	1.48 ± 0.07	-	-	-	-
	Ext/Int	2.76 ± 0.22	-	-	-	-

Supplementary Table S42: RMS (RMSE vs. 0) of all datasets and the RMSEs for all combinations for the datasets shown above before REFRAME.

**REFRAMEd**		RMS	RMSE vs.			
			PiG wand	MA	KAD	MiKneeSoTA
PiG	Flex/Ex	22.81 ± 0.35	0.46 ± 0.04	0.47 ± 0.04	0.47 ± 0.05	3.37 ± 0.38
	Abd/Add	1.78 ± 0.29	0.81 ± 0.02	0.81 ± 0.03	0.83 ± 0.03	1.83 ± 0.17
	Ext/Int	2.60 ± 0.94	0.90 ± 0.11	0.91 ± 0.12	0.86 ± 0.10	3.04 ± 0.09
PiG wand	Flex/Ex	22.78 ± 0.33	-	0.01 ± 0.00	0.10 ± 0.01	3.22 ± 0.35
	Abd/Add	1.19 ± 0.23	-	0.01 ± 0.00	0.04 ± 0.02	1.12 ± 0.19
	Ext/Int	2.49 ± 0.58	-	0.02 ± 0.00	0.10 ± 0.01	2.94 ± 0.11
MA	Flex/Ex	22.78 ± 0.33	-	-	0.10 ± 0.01	3.22 ± 0.35
	Abd/Add	1.20 ± 0.23	-	-	0.04 ± 0.02	1.13 ± 0.19
	Ext/Int	2.49 ± 0.58	-	-	0.11 ± 0.01	2.94 ± 0.11
KAD	Flex/Ex	22.86 ± 0.33	-	-	-	3.21 ± 0.34
	Abd/Add	1.20 ± 0.24	-	-	-	1.11 ± 0.18
	Ext/Int	2.55 ± 0.52	-	-	-	3.00 ± 0.10
MiKneeSoTA	Flex/Ex	22.80 ± 0.38	-	-	-	-
	Abd/Add	0.58 ± 0.07	-	-	-	-
	Ext/Int	1.04 ± 0.22	-	-	-	-

Supplementary Table S43: RMS (RMSE vs. 0) of all datasets and the RMSEs for all combinations for the datasets shown above after REFRAME.

3.15 Right Knee of Subject 8

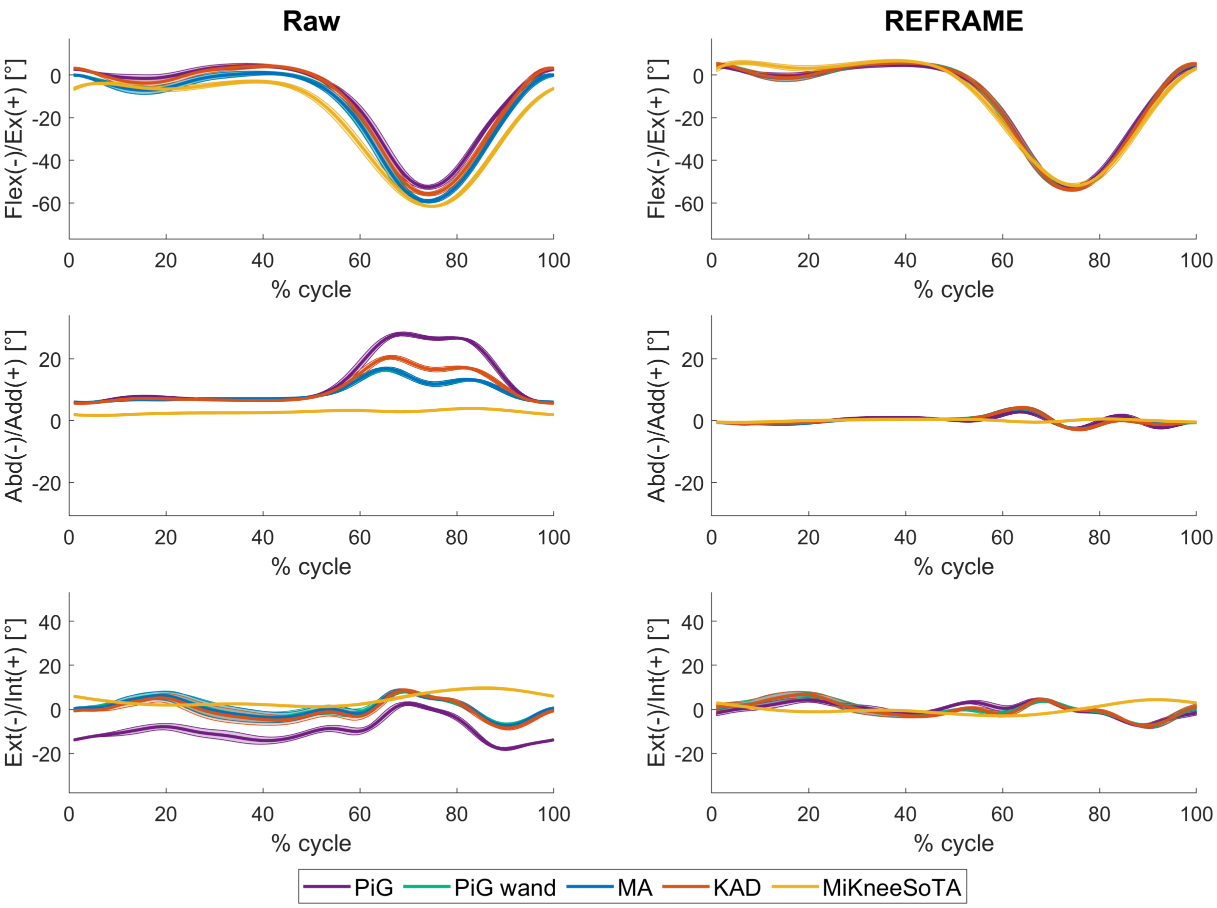

Supplementary Figure S16: Mean kinematics for all 5 markersets with standard deviation over all nine steps for knee 15 (subject 8, right). Left Column: raw kinematics, right column: REFRAMEd kinematics.

	Femur					Tibia				
markerset	PiG	PiG wand	MA	KAD	MiKneeSoTA	PiG	PiG wand	MA	KAD	MiKneeSoTA
Rx	-1.6 ± 0.3	-5.0 ± 0.2	-5.0 ± 0.2	-1.8 ± 0.2	-9.8 ± 0.9	0.0 ± 0.0	0.0 ± 0.0	0.0 ± 0.0	0.0 ± 0.0	0.0 ± 0.0
Ry	2.9 ± 1.3	2.0 ± 2.1	1.9 ± 2.1	1.1 ± 2.2	4.6 ± 0.7	-5.2 ± 1.2	-5.5 ± 2.0	-5.5 ± 2.0	-6.4 ± 2.1	2.0 ± 0.8
Rz	-27.9 ± 0.4	-9.8 ± 0.3	-9.9 ± 0.3	-15.3 ± 0.4	-3.0 ± 0.5	-15.9 ± 1.2	-9.4 ± 1.2	-9.4 ± 1.2	-13.6 ± 1.2	-6.3 ± 0.4

Supplementary Table S44: Transformations of segment frames for REFRAMEd datasets in the curves above.

**Raw data**		RMS	RMSE vs.			
			PiG wand	MA	KAD	MiKneeSoTA
PiG	Flex/Ex	21.88 ± 0.35	5.33 ± 0.13	5.40 ± 0.13	2.33 ± 0.10	10.53 ± 0.83
	Abd/Add	15.18 ± 0.21	6.50 ± 0.10	6.44 ± 0.10	4.37 ± 0.09	12.73 ± 0.30
	Ext/Int	11.10 ± 0.66	10.82 ± 0.44	10.72 ± 0.45	9.76 ± 0.44	15.30 ± 0.66
PiG wand	Flex/Ex	26.02 ± 0.32	-	0.20 ± 0.01	3.42 ± 0.01	5.94 ± 0.88
	Abd/Add	9.60 ± 0.12	-	0.11 ± 0.01	2.16 ± 0.02	6.94 ± 0.21
	Ext/Int	4.34 ± 0.49	-	0.45 ± 0.05	1.22 ± 0.02	6.57 ± 0.48
MA	Flex/Ex	26.08 ± 0.33	-	-	3.47 ± 0.02	5.93 ± 0.88
	Abd/Add	9.66 ± 0.13	-	-	2.10 ± 0.02	7.00 ± 0.21
	Ext/Int	4.42 ± 0.48	-	-	1.03 ± 0.00	6.68 ± 0.49
KAD	Flex/Ex	23.92 ± 0.31	-	-	-	8.92 ± 0.96
	Abd/Add	11.29 ± 0.16	-	-	-	8.71 ± 0.25
	Ext/Int	4.63 ± 0.58	-	-	-	7.27 ± 0.61
MiKneeSoTA	Flex/Ex	29.46 ± 0.33	-	-	-	-
	Abd/Add	2.79 ± 0.12	-	-	-	-
	Ext/Int	5.22 ± 0.22	-	-	-	-

Supplementary Table S45: RMS (RMSE vs. 0) of all datasets and the RMSEs for all combinations for the datasets shown above before REFRAME.

**REFRAMEd**		RMS	RMSE vs.			
			PiG wand	MA	KAD	MiKneeSoTA
PiG	Flex/Ex	22.66 ± 0.35	0.87 ± 0.03	0.88 ± 0.04	0.92 ± 0.04	3.27 ± 0.61
	Abd/Add	1.32 ± 0.21	0.65 ± 0.05	0.67 ± 0.05	0.68 ± 0.06	1.31 ± 0.11
	Ext/Int	3.12 ± 0.66	1.74 ± 0.13	1.79 ± 0.12	1.80 ± 0.12	5.01 ± 0.41
PiG wand	Flex/Ex	23.16 ± 0.32	-	0.19 ± 0.01	0.21 ± 0.01	3.25 ± 0.68
	Abd/Add	1.44 ± 0.12	-	0.10 ± 0.01	0.15 ± 0.02	1.49 ± 0.16
	Ext/Int	3.61 ± 0.49	-	0.43 ± 0.05	0.43 ± 0.05	5.02 ± 0.47
MA	Flex/Ex	23.19 ± 0.33	-	-	0.08 ± 0.01	3.31 ± 0.68
	Abd/Add	1.50 ± 0.13	-	-	0.12 ± 0.02	1.55 ± 0.17
	Ext/Int	3.71 ± 0.48	-	-	0.05 ± 0.00	5.08 ± 0.46
KAD	Flex/Ex	23.24 ± 0.31	-	-	-	3.27 ± 0.69
	Abd/Add	1.50 ± 0.16	-	-	-	1.56 ± 0.16
	Ext/Int	3.70 ± 0.58	-	-	-	5.06 ± 0.46
MiKneeSoTA	Flex/Ex	23.52 ± 0.33	-	-	-	-
	Abd/Add	0.38 ± 0.12	-	-	-	-
	Ext/Int	2.20 ± 0.22	-	-	-	-

Supplementary Table S46: RMS (RMSE vs. 0) of all datasets and the RMSEs for all combinations for the datasets shown above after REFRAME.

3.16 Left Knee of Subject 8

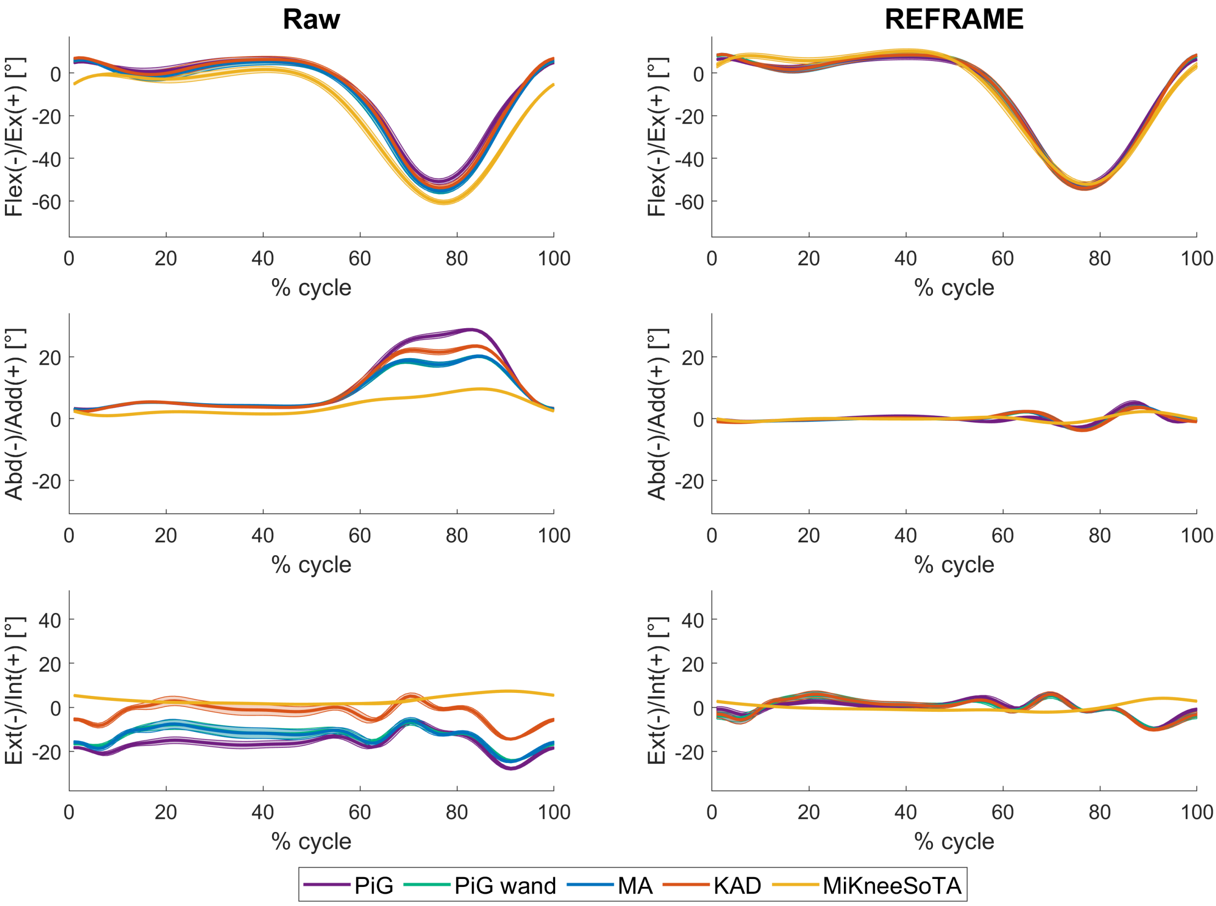

Supplementary Figure S17: Mean kinematics for all 5 markersets with standard deviation over all nine steps for knee 16 (subject 8, left). Left Column: raw kinematics, right column: REFRAMEd kinematics.

	Femur					Tibia				
markerset	PiG	PiG wand	MA	KAD	MiKneeSoTA	PiG	PiG wand	MA	KAD	MiKneeSoTA
Rx	-3.4 ± 0.2	-4.7 ± 0.2	-4.8 ± 0.2	-1.9 ± 0.1	-8.4 ± 1.4	0.0 ± 0.0	0.0 ± 0.0	0.0 ± 0.0	0.0 ± 0.0	0.0 ± 0.0
Ry	-2.0 ± 1.0	-3.8 ± 1.2	-3.7 ± 1.2	-5.0 ± 1.3	0.5 ± 0.3	-9.1 ± 0.7	-10.2 ± 1.1	-10.2 ± 1.1	-11.5 ± 1.2	-2.5 ± 0.2
Rz	-28.3 ± 0.7	-16.5 ± 0.6	-16.6 ± 0.6	-20.8 ± 0.6	-7.8 ± 0.3	-10.4 ± 1.1	-3.0 ± 1.1	-3.0 ± 1.1	-17.5 ± 1.1	-10.5 ± 0.3

Supplementary Table S47: Transformations of segment frames for REFRAMEd datasets in the curves above.

**Raw data**		RMS	RMSE vs.			
			PiG wand	MA	KAD	MiKneeSoTA
PiG	Flex/Ex	21.07 ± 0.61	2.85 ± 0.05	2.91 ± 0.06	1.88 ± 0.05	9.79 ± 1.07
	Abd/Add	14.23 ± 0.25	3.95 ± 0.08	3.91 ± 0.08	2.22 ± 0.05	9.50 ± 0.24
	Ext/Int	17.12 ± 0.72	4.29 ± 0.44	4.16 ± 0.43	14.19 ± 0.43	20.70 ± 0.63
PiG wand	Flex/Ex	23.46 ± 0.59	-	0.19 ± 0.01	1.33 ± 0.01	7.73 ± 1.00
	Abd/Add	10.65 ± 0.18	-	0.12 ± 0.02	1.81 ± 0.04	5.85 ± 0.16
	Ext/Int	13.70 ± 0.81	-	0.50 ± 0.03	10.47 ± 0.02	17.40 ± 0.73
MA	Flex/Ex	23.50 ± 0.60	-	-	1.37 ± 0.01	7.72 ± 0.99
	Abd/Add	10.71 ± 0.18	-	-	1.76 ± 0.04	5.92 ± 0.16
	Ext/Int	13.86 ± 0.81	-	-	10.59 ± 0.02	17.55 ± 0.73
KAD	Flex/Ex	22.64 ± 0.58	-	-	-	8.93 ± 1.05
	Abd/Add	12.28 ± 0.22	-	-	-	7.51 ± 0.19
	Ext/Int	5.39 ± 0.23	-	-	-	8.52 ± 0.38
MiKneeSoTA	Flex/Ex	28.00 ± 0.66	-	-	-	-
	Abd/Add	4.96 ± 0.11	-	-	-	-
	Ext/Int	3.94 ± 0.23	-	-	-	-

Supplementary Table S48: RMS (RMSE vs. 0) of all datasets and the RMSEs for all combinations for the datasets shown above before REFRAME.

**REFRAMEd**		RMS	RMSE vs.			
			PiG wand	MA	KAD	MiKneeSoTA
PiG	Flex/Ex	22.52 ± 0.61	0.95 ± 0.04	0.99 ± 0.04	1.08 ± 0.04	3.29 ± 0.70
	Abd/Add	1.57 ± 0.25	0.90 ± 0.06	0.93 ± 0.05	0.95 ± 0.06	1.15 ± 0.16
	Ext/Int	3.43 ± 0.72	1.64 ± 0.21	1.63 ± 0.23	1.65 ± 0.24	5.00 ± 0.32
PiG wand	Flex/Ex	23.22 ± 0.59	-	0.20 ± 0.02	0.25 ± 0.02	3.25 ± 0.71
	Abd/Add	1.52 ± 0.18	-	0.10 ± 0.01	0.17 ± 0.04	1.13 ± 0.13
	Ext/Int	4.06 ± 0.81	-	0.46 ± 0.04	0.47 ± 0.04	5.60 ± 0.51
MA	Flex/Ex	23.24 ± 0.60	-	-	0.15 ± 0.01	3.33 ± 0.70
	Abd/Add	1.56 ± 0.18	-	-	0.16 ± 0.04	1.18 ± 0.15
	Ext/Int	4.15 ± 0.81	-	-	0.07 ± 0.01	5.65 ± 0.50
KAD	Flex/Ex	23.34 ± 0.58	-	-	-	3.29 ± 0.70
	Abd/Add	1.53 ± 0.22	-	-	-	1.20 ± 0.14
	Ext/Int	4.19 ± 0.23	-	-	-	5.70 ± 0.51
MiKneeSoTA	Flex/Ex	23.61 ± 0.66	-	-	-	-
	Abd/Add	0.89 ± 0.11	-	-	-	-
	Ext/Int	1.82 ± 0.23	-	-	-	-

Supplementary Table S49: RMS (RMSE vs. 0) of all datasets and the RMSEs for all combinations for the datasets shown above after REFRAME.

3.17 Right Knee of Subject 9

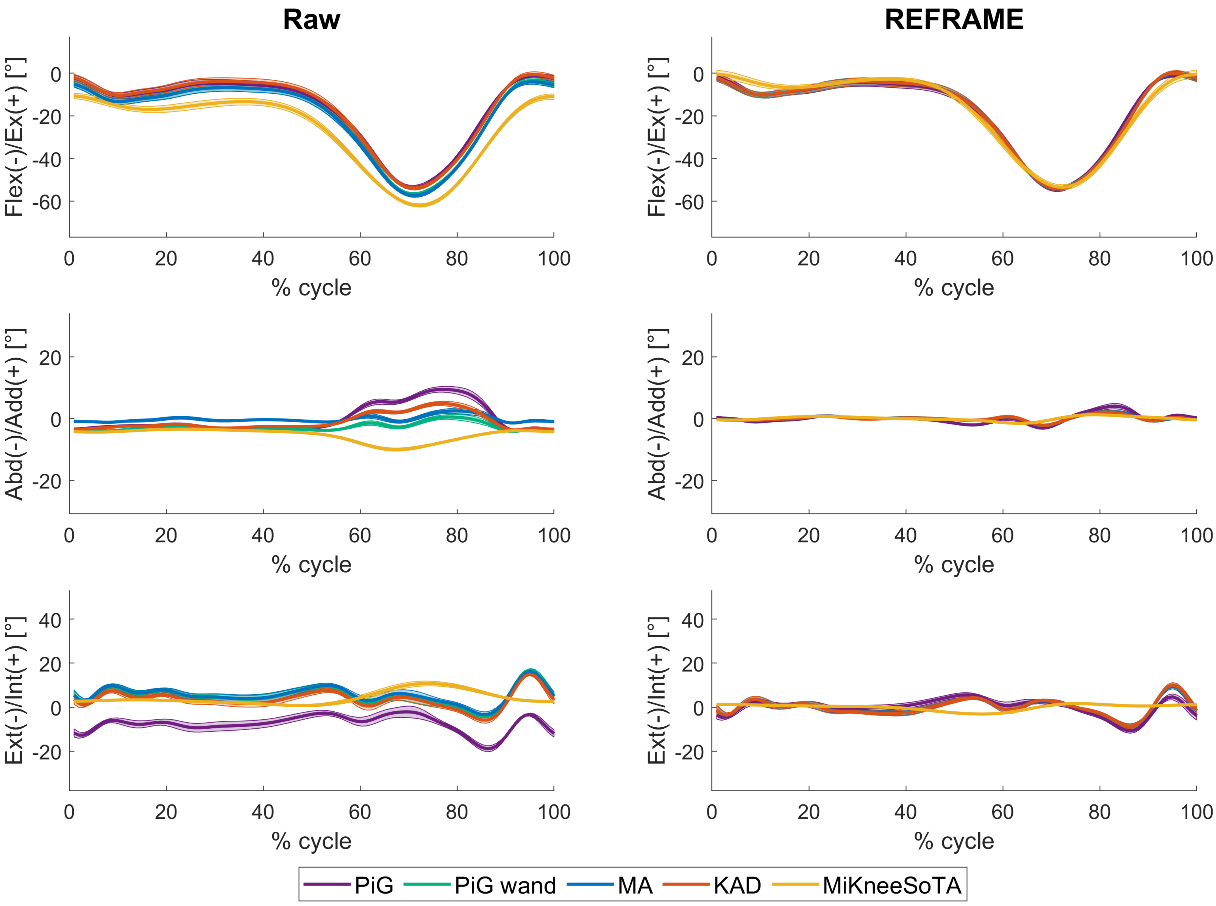

Supplementary Figure S18: Mean kinematics for all 5 markersets with standard deviation over all nine steps for knee 17 (subject 9, right). Left Column: raw kinematics, right column: REFRAMEd kinematics.

	Femur					Tibia				
markerset	PiG	PiG wand	MA	KAD	MiKneeSoTA	PiG	PiG wand	MA	KAD	MiKneeSoTA
Rx	-0.3 ± 0.3	-2.4 ± 0.3	-2.5 ± 0.3	0.4 ± 0.2	-10.9 ± 1.1	0.0 ± 0.0	0.0 ± 0.0	0.0 ± 0.0	0.0 ± 0.0	0.0 ± 0.0
Ry	-2.9 ± 2.3	-6.7 ± 2.1	-4.5 ± 2.1	-7.4 ± 2.1	10.6 ± 1.3	1.3 ± 2.3	-3.2 ± 2.1	-3.9 ± 2.1	-4.1 ± 2.1	14.5 ± 1.3
Rz	-14.6 ± 1.4	-0.4 ± 1.3	-0.1 ± 1.2	-6.0 ± 1.3	2.0 ± 0.5	-6.6 ± 1.5	-7.4 ± 1.1	-7.0 ± 1.1	-11.1 ± 1.1	0.6 ± 0.5

Supplementary Table S50: Transformations of segment frames for REFRAMEd datasets in the curves above.

**Raw data**		RMS	RMSE vs.			
			PiG wand	MA	KAD	MiKneeSoTA
PiG	Flex/Ex	23.81 ± 0.49	2.91 ± 0.09	3.14 ± 0.09	0.88 ± 0.05	10.60 ± 0.87
	Abd/Add	4.37 ± 0.28	4.31 ± 0.08	3.60 ± 0.09	2.09 ± 0.09	8.16 ± 0.42
	Ext/Int	8.63 ± 0.67	13.21 ± 0.24	13.54 ± 0.20	11.51 ± 0.22	12.86 ± 0.69
PiG wand	Flex/Ex	26.26 ± 0.45	-	0.27 ± 0.01	2.97 ± 0.02	7.92 ± 0.85
	Abd/Add	3.06 ± 0.22	-	2.58 ± 0.01	2.32 ± 0.05	3.90 ± 0.38
	Ext/Int	6.67 ± 0.29	-	0.72 ± 0.02	1.75 ± 0.01	6.34 ± 0.58
MA	Flex/Ex	26.48 ± 0.45	-	-	3.20 ± 0.02	7.71 ± 0.86
	Abd/Add	1.13 ± 0.17	-	-	2.09 ± 0.09	5.73 ± 0.37
	Ext/Int	6.85 ± 0.34	-	-	2.13 ± 0.04	6.02 ± 0.57
KAD	Flex/Ex	24.01 ± 0.43	-	-	-	10.72 ± 0.92
	Abd/Add	2.86 ± 0.16	-	-	-	6.17 ± 0.38
	Ext/Int	5.37 ± 0.20	-	-	-	5.93 ± 0.57
MiKneeSoTA	Flex/Ex	32.20 ± 0.53	-	-	-	-
	Abd/Add	5.68 ± 0.23	-	-	-	-
	Ext/Int	5.14 ± 0.50	-	-	-	-

Supplementary Table S51: RMS (RMSE vs. 0) of all datasets and the RMSEs for all combinations for the datasets shown above before REFRAME.

**REFRAMEd**		RMS	RMSE vs.			
			PiG wand	MA	KAD	MiKneeSoTA
PiG	Flex/Ex	24.16 ± 0.49	0.71 ± 0.07	0.76 ± 0.06	0.76 ± 0.06	3.20 ± 0.49
	Abd/Add	1.51 ± 0.28	0.75 ± 0.07	0.78 ± 0.07	0.82 ± 0.07	1.20 ± 0.18
	Ext/Int	3.80 ± 0.67	2.09 ± 0.24	1.98 ± 0.24	2.12 ± 0.24	4.62 ± 0.43
PiG wand	Flex/Ex	24.28 ± 0.45	-	0.16 ± 0.02	0.09 ± 0.00	3.00 ± 0.47
	Abd/Add	0.91 ± 0.22	-	0.05 ± 0.00	0.10 ± 0.01	0.79 ± 0.14
	Ext/Int	3.68 ± 0.29	-	0.17 ± 0.01	0.06 ± 0.00	4.15 ± 0.29
MA	Flex/Ex	24.34 ± 0.45	-	-	0.14 ± 0.01	2.94 ± 0.46
	Abd/Add	0.89 ± 0.17	-	-	0.11 ± 0.01	0.78 ± 0.14
	Ext/Int	3.58 ± 0.34	-	-	0.19 ± 0.01	4.07 ± 0.29
KAD	Flex/Ex	24.33 ± 0.43	-	-	-	2.98 ± 0.46
	Abd/Add	0.86 ± 0.16	-	-	-	0.77 ± 0.13
	Ext/Int	3.67 ± 0.20	-	-	-	4.14 ± 0.29
MiKneeSoTA	Flex/Ex	24.75 ± 0.53	-	-	-	-
	Abd/Add	0.71 ± 0.23	-	-	-	-
	Ext/Int	1.40 ± 0.50	-	-	-	-

Supplementary Table S52: RMS (RMSE vs. 0) of all datasets and the RMSEs for all combinations for the datasets shown above after REFRAME.

3.18 Left Knee of Subject 9

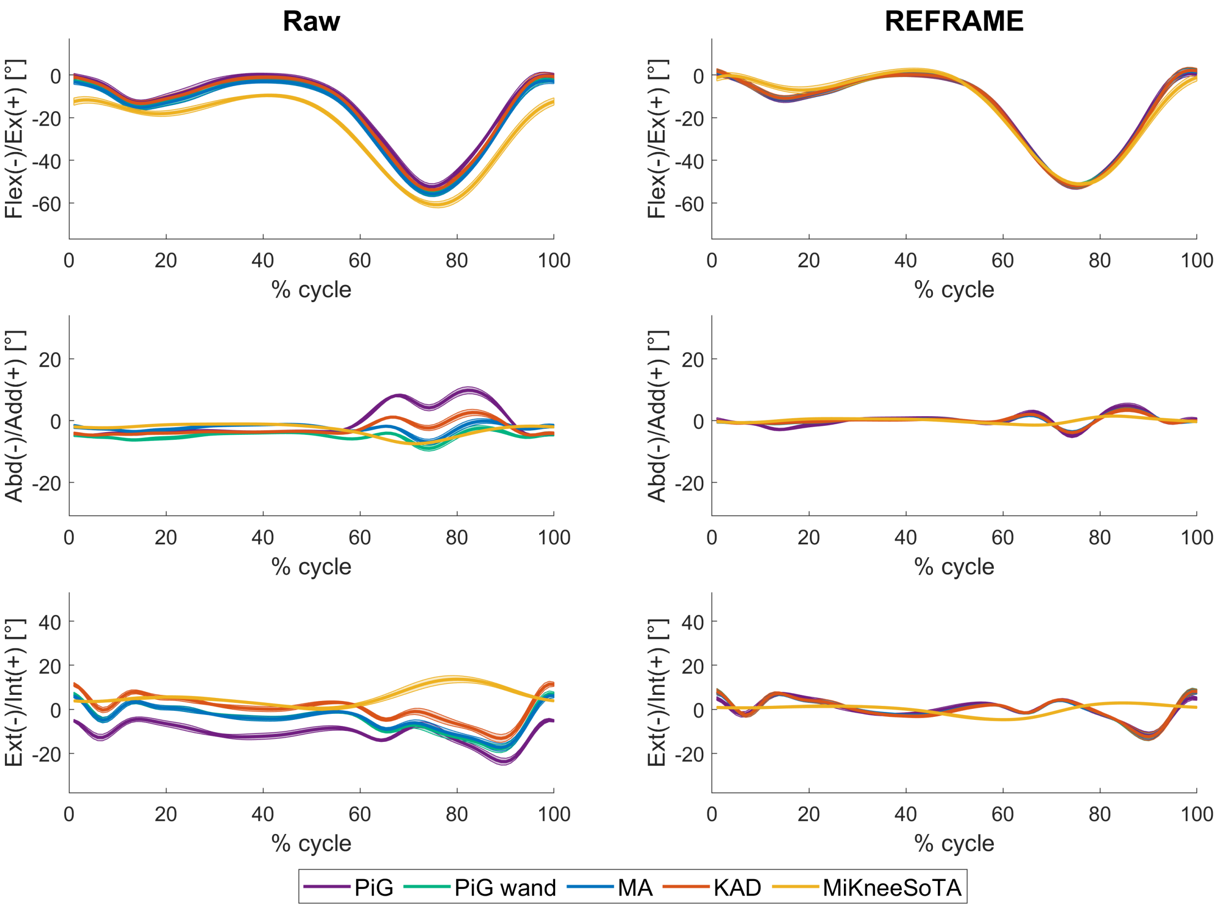

Supplementary Figure S19: Mean kinematics for all 5 markersets with standard deviation over all nine steps for knee 18 (subject 9, left). Left Column: raw kinematics, right column: REFRAMEd kinematics.

	Femur					Tibia				
markerset	PiG	PiG wand	MA	KAD	MiKneeSoTA	PiG	PiG wand	MA	KAD	MiKneeSoTA
Rx	-1.1 ± 0.3	-3.3 ± 0.1	-3.7 ± 0.1	-1.2 ± 0.2	-12.2 ± 0.8	0.0 ± 0.0	0.0 ± 0.0	0.0 ± 0.0	0.0 ± 0.0	0.0 ± 0.0
Ry	-7.9 ± 1.8	-10.3 ± 1.8	-8.4 ± 1.8	-11.4 ± 1.8	12.0 ± 1.9	-3.5 ± 1.8	-6.0 ± 1.9	-6.7 ± 1.9	-7.5 ± 1.8	13.6 ± 2.0
Rz	-13.3 ± 1.1	6.1 ± 1.0	6.0 ± 1.0	-1.2 ± 1.0	1.5 ± 0.7	-3.1 ± 1.0	7.6 ± 1.0	7.8 ± 1.0	-4.3 ± 1.0	-1.3 ± 0.6

Supplementary Table S53: Transformations of segment frames for REFRAMEd datasets in the curves above.

**Raw data**		RMS	RMSE vs.			
			PiG wand	MA	KAD	MiKneeSoTA
PiG	Flex/Ex	22.38 ± 0.62	3.30 ± 0.08	3.56 ± 0.09	1.68 ± 0.06	11.66 ± 0.82
	Abd/Add	4.85 ± 0.26	6.59 ± 0.10	5.48 ± 0.10	3.52 ± 0.07	6.85 ± 0.37
	Ext/Int	11.99 ± 0.49	7.38 ± 0.35	7.34 ± 0.33	11.70 ± 0.35	18.06 ± 0.84
PiG wand	Flex/Ex	25.06 ± 0.59	-	0.31 ± 0.02	1.73 ± 0.02	8.59 ± 0.77
	Abd/Add	5.21 ± 0.26	-	2.52 ± 0.01	3.13 ± 0.06	2.82 ± 0.23
	Ext/Int	7.53 ± 0.55	-	0.59 ± 0.01	4.82 ± 0.03	13.54 ± 0.71
MA	Flex/Ex	25.28 ± 0.60	-	-	2.01 ± 0.03	8.33 ± 0.76
	Abd/Add	2.89 ± 0.29	-	-	2.37 ± 0.04	1.73 ± 0.21
	Ext/Int	7.08 ± 0.54	-	-	4.59 ± 0.03	13.10 ± 0.71
KAD	Flex/Ex	23.81 ± 0.59	-	-	-	10.22 ± 0.79
	Abd/Add	3.47 ± 0.15	-	-	-	3.69 ± 0.27
	Ext/Int	5.74 ± 0.48	-	-	-	10.12 ± 0.62
MiKneeSoTA	Flex/Ex	31.27 ± 0.73	-	-	-	-
	Abd/Add	3.49 ± 0.14	-	-	-	-
	Ext/Int	6.79 ± 0.63	-	-	-	-

Supplementary Table S54: RMS (RMSE vs. 0) of all datasets and the RMSEs for all combinations for the datasets shown above before REFRAME.

**REFRAMEd**		RMS	RMSE vs.			
			PiG wand	MA	KAD	MiKneeSoTA
PiG	Flex/Ex	22.82 ± 0.62	0.53 ± 0.06	0.54 ± 0.05	0.68 ± 0.05	3.22 ± 0.37
	Abd/Add	2.04 ± 0.26	0.76 ± 0.05	0.80 ± 0.06	0.80 ± 0.06	1.99 ± 0.20
	Ext/Int	4.33 ± 0.49	1.11 ± 0.15	1.00 ± 0.13	1.10 ± 0.15	5.42 ± 0.35
PiG wand	Flex/Ex	22.94 ± 0.59	-	0.17 ± 0.01	0.20 ± 0.01	3.04 ± 0.36
	Abd/Add	1.46 ± 0.26	-	0.06 ± 0.00	0.10 ± 0.02	1.43 ± 0.16
	Ext/Int	4.62 ± 0.55	-	0.16 ± 0.01	0.15 ± 0.02	5.56 ± 0.32
MA	Flex/Ex	23.02 ± 0.60	-	-	0.20 ± 0.02	2.99 ± 0.34
	Abd/Add	1.43 ± 0.29	-	-	0.11 ± 0.02	1.39 ± 0.16
	Ext/Int	4.51 ± 0.54	-	-	0.22 ± 0.02	5.48 ± 0.31
KAD	Flex/Ex	23.06 ± 0.59	-	-	-	2.98 ± 0.34
	Abd/Add	1.44 ± 0.15	-	-	-	1.44 ± 0.15
	Ext/Int	4.62 ± 0.48	-	-	-	5.55 ± 0.32
MiKneeSoTA	Flex/Ex	23.29 ± 0.73	-	-	-	-
	Abd/Add	0.78 ± 0.14	-	-	-	-
	Ext/Int	2.29 ± 0.63	-	-	-	-

Supplementary Table S55: RMS (RMSE vs. 0) of all datasets and the RMSEs for all combinations for the datasets shown above after REFRAME.

3.19 Right Knee of Subject 10

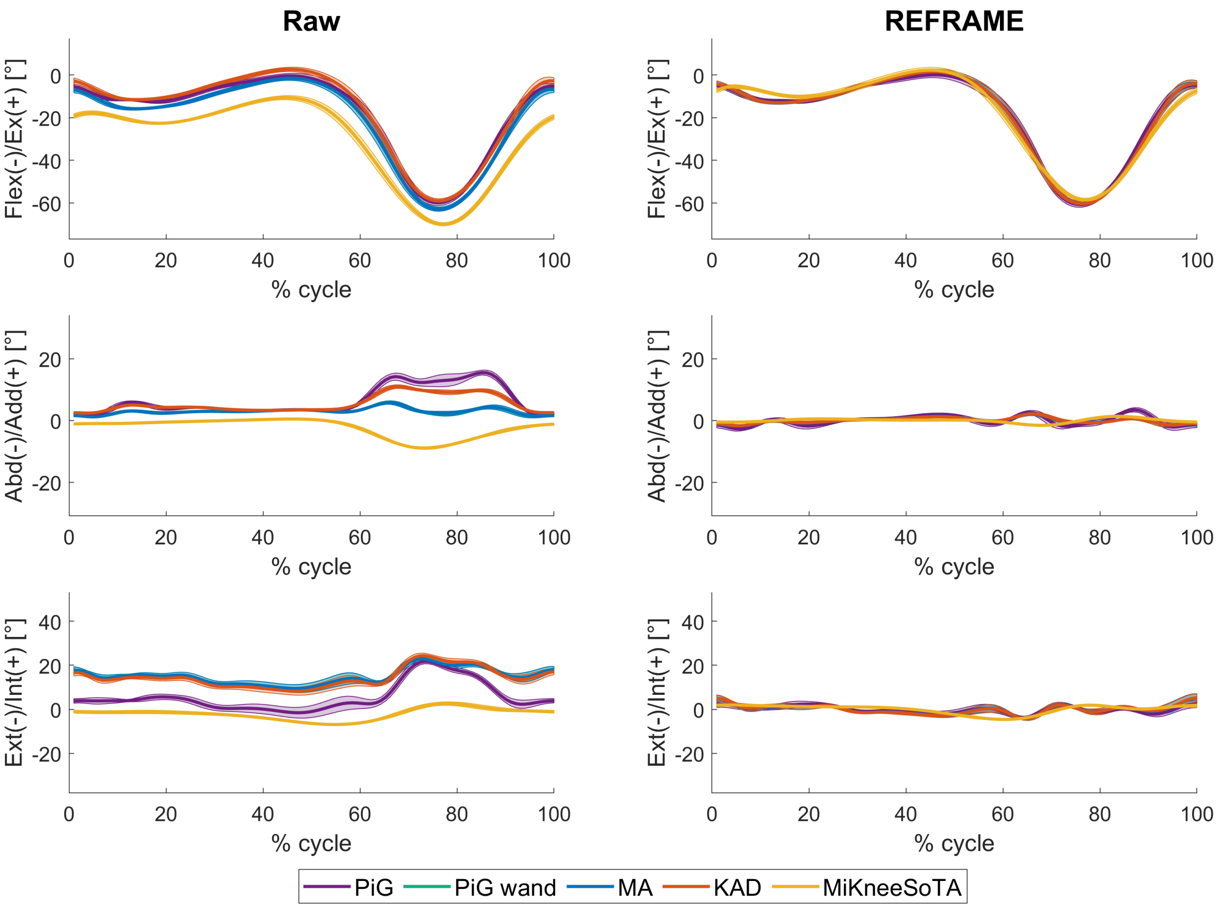

Supplementary Figure S20: Mean kinematics for all 5 markersets with standard deviation over all nine steps for knee 19 (subject 10, right). Left Column: raw kinematics, right column: REFRAMEd kinematics.

	Femur					Tibia				
markerset	PiG	PiG wand	MA	KAD	MiKneeSoTA	PiG	PiG wand	MA	KAD	MiKneeSoTA
Rx	-0.9 ± 0.2	-4.1 ± 0.3	-4.1 ± 0.3	0.3 ± 0.3	-12.1 ± 0.8	0.0 ± 0.0	0.0 ± 0.0	0.0 ± 0.0	0.0 ± 0.0	0.0 ± 0.0
Ry	11.7 ± 2.1	7.7 ± 1.4	7.6 ± 1.4	6.8 ± 1.4	8.6 ± 0.6	10.1 ± 1.7	5.5 ± 1.3	5.4 ± 1.3	4.3 ± 1.3	8.5 ± 0.6
Rz	-20.6 ± 1.3	-4.8 ± 0.6	-4.8 ± 0.6	-12.1 ± 0.6	5.5 ± 0.3	-21.0 ± 2.1	-17.2 ± 1.0	-17.3 ± 1.0	-23.5 ± 1.0	9.5 ± 0.5

Supplementary Table S56: Transformations of segment frames for REFRAMEd datasets in the curves above.

**Raw data**		RMS	RMSE vs.			
			PiG wand	MA	KAD	MiKneeSoTA
PiG	Flex/Ex	26.17 ± 0.56	3.19 ± 0.15	3.21 ± 0.15	1.86 ± 0.13	12.91 ± 0.84
	Abd/Add	7.90 ± 0.45	5.35 ± 0.30	5.32 ± 0.30	2.17 ± 0.26	11.45 ± 0.46
	Ext/Int	8.69 ± 0.27	10.03 ± 0.31	10.03 ± 0.31	9.19 ± 0.31	9.21 ± 0.77
PiG wand	Flex/Ex	28.77 ± 0.48	-	0.05 ± 0.00	4.16 ± 0.02	10.09 ± 0.84
	Abd/Add	3.14 ± 0.17	-	0.05 ± 0.00	3.37 ± 0.05	6.33 ± 0.21
	Ext/Int	15.51 ± 0.43	-	0.07 ± 0.00	1.08 ± 0.02	17.26 ± 0.81
MA	Flex/Ex	28.79 ± 0.48	-	-	4.16 ± 0.02	10.08 ± 0.84
	Abd/Add	3.15 ± 0.17	-	-	3.34 ± 0.05	6.36 ± 0.21
	Ext/Int	15.49 ± 0.43	-	-	1.09 ± 0.02	17.25 ± 0.81
KAD	Flex/Ex	25.89 ± 0.47	-	-	-	14.08 ± 0.84
	Abd/Add	6.09 ± 0.19	-	-	-	9.60 ± 0.21
	Ext/Int	15.11 ± 0.40	-	-	-	16.73 ± 0.79
MiKneeSoTA	Flex/Ex	36.17 ± 0.52	-	-	-	-
	Abd/Add	3.81 ± 0.08	-	-	-	-
	Ext/Int	3.37 ± 0.16	-	-	-	-

Supplementary Table S57: RMS (RMSE vs. 0) of all datasets and the RMSEs for all combinations for the datasets shown above before REFRAME.

**REFRAMEd**		RMS	RMSE vs.			
			PiG wand	MA	KAD	MiKneeSoTA
PiG	Flex/Ex	26.55 ± 0.56	1.02 ± 0.07	1.03 ± 0.08	1.02 ± 0.07	3.59 ± 0.64
	Abd/Add	1.68 ± 0.45	0.99 ± 0.07	0.98 ± 0.07	1.02 ± 0.07	1.68 ± 0.29
	Ext/Int	1.85 ± 0.27	1.05 ± 0.16	1.01 ± 0.15	1.09 ± 0.17	2.09 ± 0.24
PiG wand	Flex/Ex	26.79 ± 0.48	-	0.04 ± 0.00	0.07 ± 0.01	3.27 ± 0.64
	Abd/Add	1.02 ± 0.17	-	0.03 ± 0.00	0.07 ± 0.01	1.32 ± 0.23
	Ext/Int	2.25 ± 0.43	-	0.07 ± 0.00	0.08 ± 0.01	2.21 ± 0.26
MA	Flex/Ex	26.80 ± 0.48	-	-	0.08 ± 0.01	3.27 ± 0.64
	Abd/Add	1.01 ± 0.17	-	-	0.07 ± 0.01	1.30 ± 0.23
	Ext/Int	2.21 ± 0.43	-	-	0.13 ± 0.01	2.22 ± 0.25
KAD	Flex/Ex	26.83 ± 0.47	-	-	-	3.26 ± 0.64
	Abd/Add	0.98 ± 0.19	-	-	-	1.30 ± 0.22
	Ext/Int	2.28 ± 0.40	-	-	-	2.23 ± 0.27
MiKneeSoTA	Flex/Ex	26.75 ± 0.52	-	-	-	-
	Abd/Add	0.69 ± 0.08	-	-	-	-
	Ext/Int	2.06 ± 0.16	-	-	-	-

Supplementary Table S58: RMS (RMSE vs. 0) of all datasets and the RMSEs for all combinations for the datasets shown above after REFRAME.

3.20 Left Knee of Subject 10

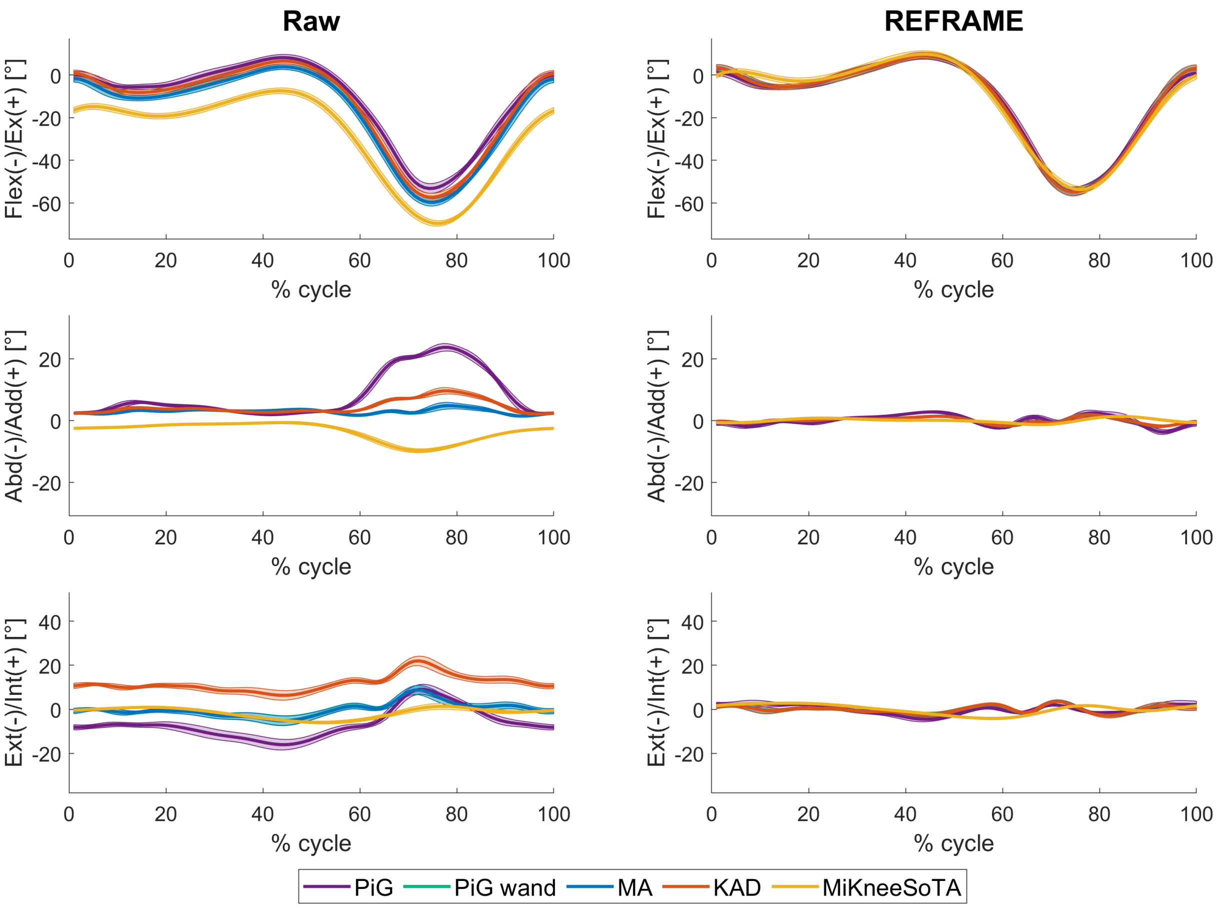

Supplementary Figure S21: Mean kinematics for all 5 markersets with standard deviation over all nine steps for knee 20, (subject 10, left). Left Column: raw kinematics, right column: REFRAMEd kinematics.

	Femur					Tibia				
markerset	PiG	PiG wand	MA	KAD	MiKneeSoTA	PiG	PiG wand	MA	KAD	MiKneeSoTA
Rx	0.5 ± 0.7	-5.1 ± 0.1	-5.1 ± 0.1	-3.2 ± 0.5	-16.5 ± 1.1	0.0 ± 0.0	0.0 ± 0.0	0.0 ± 0.0	0.0 ± 0.0	0.0 ± 0.0
Ry	9.5 ± 2.9	6.7 ± 2.4	6.8 ± 2.4	6.5 ± 2.5	5.5 ± 1.5	5.9 ± 2.7	3.9 ± 2.3	4.0 ± 2.3	3.4 ± 2.4	7.4 ± 1.5
Rz	-27.2 ± 1.0	-3.5 ± 1.0	-3.5 ± 1.0	-8.9 ± 1.0	6.5 ± 0.3	-16.8 ± 1.5	-1.9 ± 1.0	-1.9 ± 1.0	-18.8 ± 1.0	8.7 ± 0.3

Supplementary Table S59: Transformations of segment frames for REFRAMEd datasets in the curves above.

**Raw data**		RMS	RMSE vs.			
			PiG wand	MA	KAD	MiKneeSoTA
PiG	Flex/Ex	22.55 ± 0.80	5.46 ± 0.12	5.48 ± 0.12	3.13 ± 0.12	16.97 ± 1.18
	Abd/Add	10.98 ± 0.34	8.76 ± 0.19	8.74 ± 0.19	6.47 ± 0.16	15.46 ± 0.41
	Ext/Int	9.31 ± 1.15	7.55 ± 0.35	7.49 ± 0.35	18.59 ± 0.31	7.48 ± 1.12
PiG wand	Flex/Ex	26.70 ± 0.70	-	0.05 ± 0.00	2.57 ± 0.01	11.86 ± 1.14
	Abd/Add	3.05 ± 0.13	-	0.04 ± 0.00	2.30 ± 0.06	7.19 ± 0.29
	Ext/Int	3.34 ± 0.83	-	0.10 ± 0.00	11.79 ± 0.02	3.68 ± 0.50
MA	Flex/Ex	26.70 ± 0.70	-	-	2.59 ± 0.01	11.84 ± 1.14
	Abd/Add	3.08 ± 0.13	-	-	2.28 ± 0.05	7.22 ± 0.29
	Ext/Int	3.38 ± 0.85	-	-	11.82 ± 0.02	3.65 ± 0.49
KAD	Flex/Ex	25.12 ± 0.70	-	-	-	14.35 ± 1.14
	Abd/Add	4.87 ± 0.20	-	-	-	9.28 ± 0.33
	Ext/Int	12.40 ± 0.43	-	-	-	13.84 ± 0.64
MiKneeSoTA	Flex/Ex	35.26 ± 0.80	-	-	-	-
	Abd/Add	4.57 ± 0.25	-	-	-	-
	Ext/Int	2.92 ± 0.27	-	-	-	-

Supplementary Table S60: RMS (RMSE vs. 0) of all datasets and the RMSEs for all combinations for the datasets shown above before REFRAME.

**REFRAMEd**		RMS	RMSE vs.			
			PiG wand	MA	KAD	MiKneeSoTA
PiG	Flex/Ex	23.35 ± 0.80	0.91 ± 0.09	0.92 ± 0.09	0.93 ± 0.09	3.04 ± 0.71
	Abd/Add	1.78 ± 0.34	0.95 ± 0.11	0.95 ± 0.12	0.95 ± 0.11	1.77 ± 0.16
	Ext/Int	2.24 ± 1.15	1.51 ± 0.11	1.44 ± 0.11	1.48 ± 0.11	2.26 ± 0.21
PiG wand	Flex/Ex	23.67 ± 0.70	-	0.04 ± 0.00	0.06 ± 0.01	2.92 ± 0.75
	Abd/Add	1.03 ± 0.13	-	0.03 ± 0.00	0.03 ± 0.01	1.02 ± 0.17
	Ext/Int	1.79 ± 0.83	-	0.09 ± 0.00	0.07 ± 0.01	2.79 ± 0.30
MA	Flex/Ex	23.67 ± 0.70	-	-	0.07 ± 0.01	2.91 ± 0.75
	Abd/Add	1.02 ± 0.13	-	-	0.04 ± 0.00	1.02 ± 0.17
	Ext/Int	1.82 ± 0.85	-	-	0.09 ± 0.00	2.75 ± 0.29
KAD	Flex/Ex	23.71 ± 0.70	-	-	-	2.91 ± 0.75
	Abd/Add	1.01 ± 0.20	-	-	-	1.00 ± 0.17
	Ext/Int	1.80 ± 0.43	-	-	-	2.79 ± 0.30
MiKneeSoTA	Flex/Ex	23.90 ± 0.80	-	-	-	-
	Abd/Add	0.72 ± 0.25	-	-	-	-
	Ext/Int	2.21 ± 0.27	-	-	-	-

Supplementary Table S61: RMS (RMSE vs. 0) of all datasets and the RMSEs for all combinations for the datasets shown above after REFRAME.

3.21 Right Knee of Subject 11

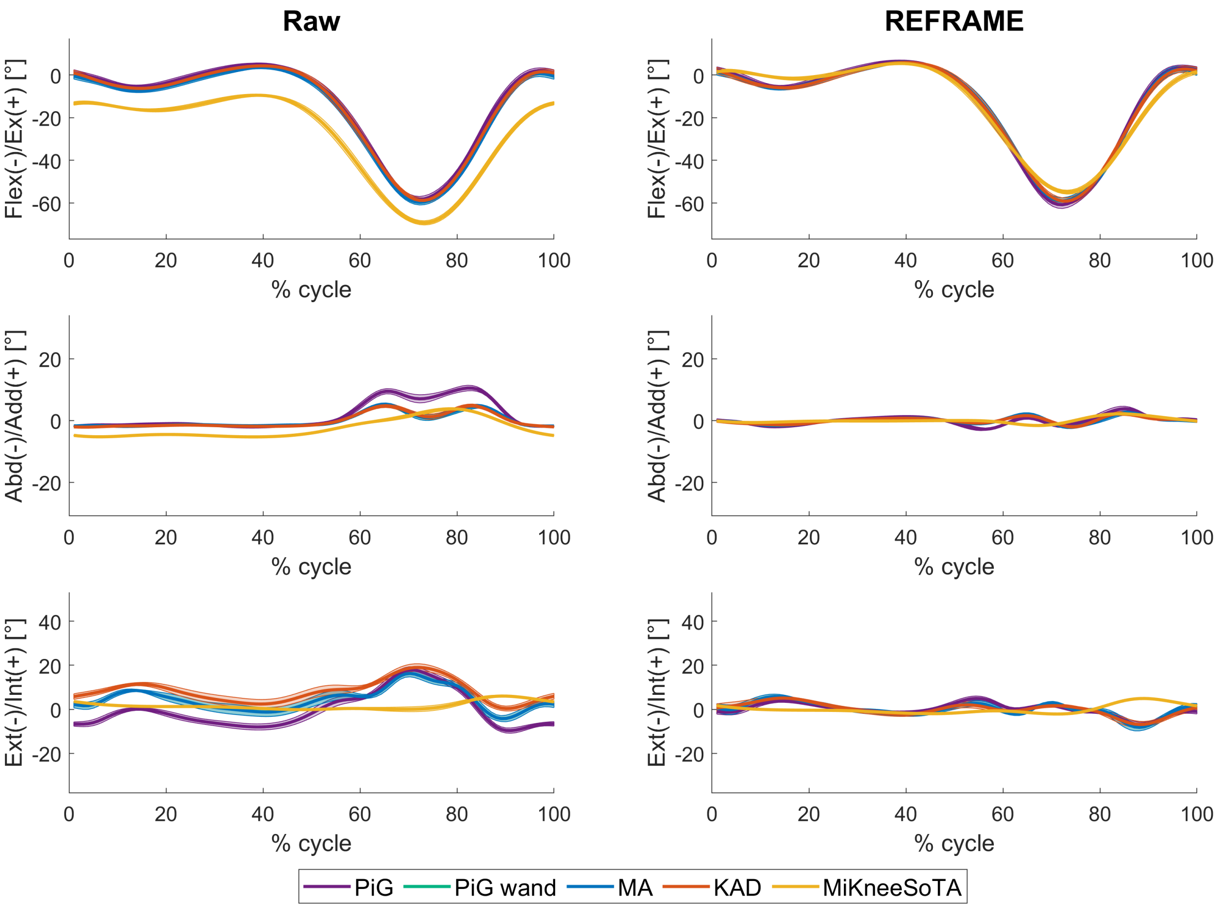

Supplementary Figure S22: Mean kinematics for all 5 markersets with standard deviation over all nine steps for knee 21 (subject 11, right). Left Column: raw kinematics, right column: REFRAMEd kinematics.

	Femur					Tibia				
markerset	PiG	PiG wand	MA	KAD	MiKneeSoTA	PiG	PiG wand	MA	KAD	MiKneeSoTA
Rx	0.7 ± 0.2	-2.4 ± 0.1	-2.4 ± 0.1	-2.1 ± 0.1	-14.8 ± 0.4	0.0 ± 0.0	0.0 ± 0.0	0.0 ± 0.0	0.0 ± 0.0	0.0 ± 0.0
Ry	14.5 ± 1.3	8.7 ± 1.7	8.6 ± 1.7	8.7 ± 1.8	-3.3 ± 1.0	15.7 ± 1.3	10.0 ± 1.7	10.0 ± 1.7	10.2 ± 1.7	1.2 ± 1.0
Rz	-19.2 ± 0.4	-9.7 ± 0.5	-9.7 ± 0.5	-9.9 ± 0.5	-7.6 ± 0.3	-13.9 ± 0.9	-11.5 ± 0.9	-11.5 ± 0.9	-15.4 ± 0.9	-9.3 ± 0.3

Supplementary Table S62: Transformations of segment frames for REFRAMEd datasets in the curves above.

**Raw data**		RMS	RMSE vs.			
			PiG wand	MA	KAD	MiKneeSoTA
PiG	Flex/Ex	24.83 ± 0.52	1.86 ± 0.05	1.87 ± 0.05	1.04 ± 0.05	15.07 ± 0.40
	Abd/Add	4.77 ± 0.27	2.89 ± 0.08	2.88 ± 0.08	2.85 ± 0.08	4.59 ± 0.27
	Ext/Int	8.04 ± 0.51	6.14 ± 0.36	6.12 ± 0.35	9.28 ± 0.37	9.17 ± 0.42
PiG wand	Flex/Ex	25.93 ± 0.46	-	0.01 ± 0.00	0.99 ± 0.02	13.33 ± 0.36
	Abd/Add	2.30 ± 0.16	-	0.02 ± 0.00	0.30 ± 0.02	3.02 ± 0.07
	Ext/Int	6.98 ± 0.49	-	0.03 ± 0.01	3.72 ± 0.03	6.91 ± 0.66
MA	Flex/Ex	25.94 ± 0.46	-	-	1.00 ± 0.02	13.32 ± 0.36
	Abd/Add	2.31 ± 0.16	-	-	0.29 ± 0.02	3.02 ± 0.07
	Ext/Int	6.97 ± 0.49	-	-	3.74 ± 0.03	6.90 ± 0.65
KAD	Flex/Ex	25.33 ± 0.47	-	-	-	14.19 ± 0.38
	Abd/Add	2.35 ± 0.14	-	-	-	2.87 ± 0.07
	Ext/Int	9.64 ± 0.57	-	-	-	8.92 ± 0.87
MiKneeSoTA	Flex/Ex	35.02 ± 0.46	-	-	-	-
	Abd/Add	3.96 ± 0.10	-	-	-	-
	Ext/Int	2.52 ± 0.21	-	-	-	-

Supplementary Table S63: RMS (RMSE vs. 0) of all datasets and the RMSEs for all combinations for the datasets shown above before REFRAME.

**REFRAMEd**		RMS	RMSE vs.			
			PiG wand	MA	KAD	MiKneeSoTA
PiG	Flex/Ex	25.84 ± 0.52	0.97 ± 0.09	0.97 ± 0.09	1.06 ± 0.09	4.03 ± 0.42
	Abd/Add	1.53 ± 0.27	0.83 ± 0.06	0.83 ± 0.06	0.85 ± 0.07	1.25 ± 0.19
	Ext/Int	2.78 ± 0.51	1.41 ± 0.13	1.42 ± 0.13	1.42 ± 0.15	4.35 ± 0.36
PiG wand	Flex/Ex	25.26 ± 0.46	-	0.01 ± 0.00	0.42 ± 0.07	3.75 ± 0.34
	Abd/Add	1.14 ± 0.16	-	0.00 ± 0.00	0.25 ± 0.02	1.20 ± 0.14
	Ext/Int	3.26 ± 0.49	-	0.02 ± 0.00	0.85 ± 0.06	4.57 ± 0.41
MA	Flex/Ex	25.26 ± 0.46	-	-	0.42 ± 0.07	3.75 ± 0.34
	Abd/Add	1.14 ± 0.16	-	-	0.25 ± 0.02	1.20 ± 0.14
	Ext/Int	3.27 ± 0.49	-	-	0.85 ± 0.06	4.56 ± 0.41
KAD	Flex/Ex	25.21 ± 0.47	-	-	-	3.61 ± 0.35
	Abd/Add	1.03 ± 0.14	-	-	-	1.09 ± 0.12
	Ext/Int	3.00 ± 0.57	-	-	-	4.38 ± 0.39
MiKneeSoTA	Flex/Ex	24.71 ± 0.46	-	-	-	-
	Abd/Add	0.89 ± 0.10	-	-	-	-
	Ext/Int	1.99 ± 0.21	-	-	-	-

Supplementary Table S64: RMS (RMSE vs. 0) of all datasets and the RMSEs for all combinations for the datasets shown above after REFRAME.

3.22 Left Knee of Subject 11

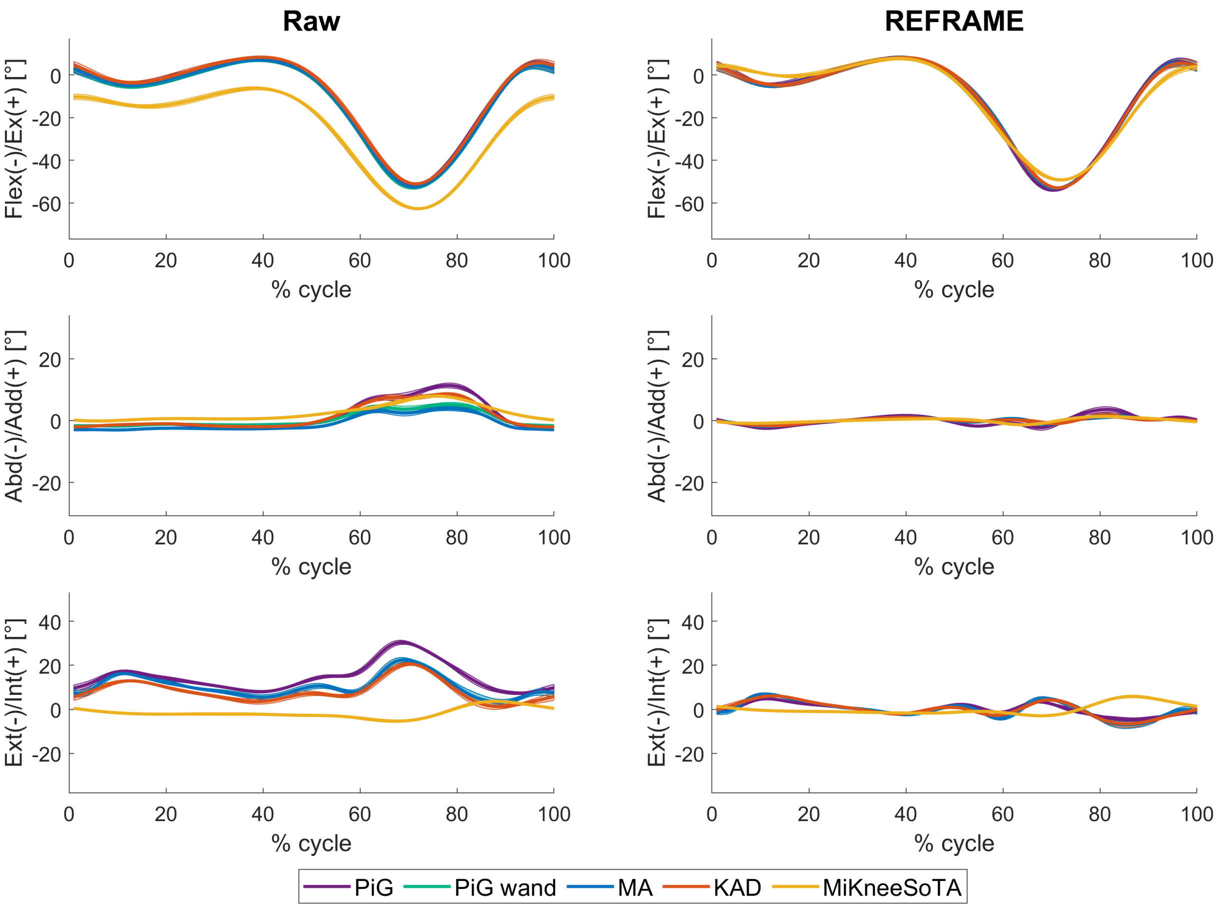

Supplementary Figure S23: Mean kinematics for all 5 markersets with standard deviation over all nine steps for knee 22 (subject 11, left). Left Column: raw kinematics, right column: REFRAMEd kinematics.

	Femur					Tibia				
markerset	PiG	PiG wand	MA	KAD	MiKneeSoTA	PiG	PiG wand	MA	KAD	MiKneeSoTA
Rx	-3.2 ± 0.2	-2.2 ± 0.2	-2.2 ± 0.2	-0.3 ± 0.1	-14.3 ± 0.5	0.0 ± 0.0	0.0 ± 0.0	0.0 ± 0.0	0.0 ± 0.0	0.0 ± 0.0
Ry	12.5 ± 0.9	6.7 ± 1.2	6.7 ± 1.2	6.1 ± 1.2	-4.3 ± 0.8	13.5 ± 0.9	7.7 ± 1.2	9.0 ± 1.2	7.1 ± 1.3	-5.3 ± 0.8
Rz	-17.5 ± 0.5	-9.9 ± 0.6	-9.9 ± 0.6	-14.3 ± 0.6	-6.4 ± 0.4	-29.2 ± 0.5	-18.7 ± 0.4	-18.6 ± 0.4	-20.6 ± 0.4	-5.2 ± 0.4

Supplementary Table S65: Transformations of segment frames for REFRAMEd datasets in the curves above.

Raw data		RMS	RMSE vs.			
			PiG wand	MA	KAD	MiKneeSoTA
PiG	Flex/Ex	21.94 ± 0.28	1.10 ± 0.04	0.92 ± 0.04	0.92 ± 0.04	14.44 ± 0.47
	Abd/Add	4.86 ± 0.22	2.44 ± 0.05	3.25 ± 0.05	1.07 ± 0.04	2.39 ± 0.15
	Ext/Int	15.90 ± 0.31	4.95 ± 0.18	4.97 ± 0.18	6.79 ± 0.22	18.02 ± 0.40
PiG wand	Flex/Ex	22.62 ± 0.28	-	0.26 ± 0.01	1.66 ± 0.01	13.55 ± 0.46
	Abd/Add	2.56 ± 0.17	-	1.28 ± 0.00	1.59 ± 0.02	2.18 ± 0.18
	Ext/Int	11.62 ± 0.38	-	0.03 ± 0.00	2.37 ± 0.04	13.81 ± 0.53
MA	Flex/Ex	22.43 ± 0.28	-	-	1.43 ± 0.01	13.75 ± 0.46
	Abd/Add	2.68 ± 0.10	-	-	2.47 ± 0.02	3.40 ± 0.19
	Ext/Int	11.60 ± 0.38	-	-	2.36 ± 0.04	13.79 ± 0.53
KAD	Flex/Ex	21.63 ± 0.28	-	-	-	15.10 ± 0.46
	Abd/Add	4.02 ± 0.20	-	-	-	2.03 ± 0.14
	Ext/Int	9.69 ± 0.36	-	-	-	11.97 ± 0.51
MiKneeSoTA	Flex/Ex	31.32 ± 0.26	-	-	-	-
	Abd/Add	3.52 ± 0.19	-	-	-	-
	Ext/Int	2.85 ± 0.30	-	-	-	-

Supplementary Table S66: RMS (RMSE vs. 0) of all datasets and the RMSEs for all combinations for the datasets shown above before REFRAME.

REFRAMEd		RMS	RMSE vs.			
			PiG wand	MA	KAD	MiKneeSoTA
PiG	Flex/Ex	22.71 ± 0.28	0.64 ± 0.04	0.64 ± 0.04	0.75 ± 0.05	3.47 ± 0.42
	Abd/Add	1.63 ± 0.22	0.93 ± 0.04	0.93 ± 0.04	0.95 ± 0.05	1.15 ± 0.23
	Ext/Int	2.57 ± 0.31	1.50 ± 0.17	1.49 ± 0.17	1.34 ± 0.15	4.59 ± 0.34
PiG wand	Flex/Ex	22.43 ± 0.28	-	0.01 ± 0.00	0.36 ± 0.05	3.36 ± 0.33
	Abd/Add	0.95 ± 0.17	-	0.01 ± 0.00	0.19 ± 0.02	0.78 ± 0.09
	Ext/Int	3.62 ± 0.38	-	0.01 ± 0.00	0.79 ± 0.09	5.44 ± 0.53
MA	Flex/Ex	22.43 ± 0.28	-	-	0.36 ± 0.05	3.36 ± 0.33
	Abd/Add	0.94 ± 0.10	-	-	0.19 ± 0.02	0.78 ± 0.09
	Ext/Int	3.62 ± 0.38	-	-	0.79 ± 0.09	5.45 ± 0.53
KAD	Flex/Ex	22.39 ± 0.28	-	-	-	3.18 ± 0.34
	Abd/Add	0.89 ± 0.20	-	-	-	0.71 ± 0.08
	Ext/Int	3.40 ± 0.36	-	-	-	5.28 ± 0.52
MiKneeSoTA	Flex/Ex	21.97 ± 0.26	-	-	-	-
	Abd/Add	0.72 ± 0.19	-	-	-	-
	Ext/Int	2.41 ± 0.30	-	-	-	-

Supplementary Table S67: RMS (RMSE vs. 0) of all datasets and the RMSEs for all combinations for the datasets shown above after REFRAME.

3.23 Right Knee of Subject 12

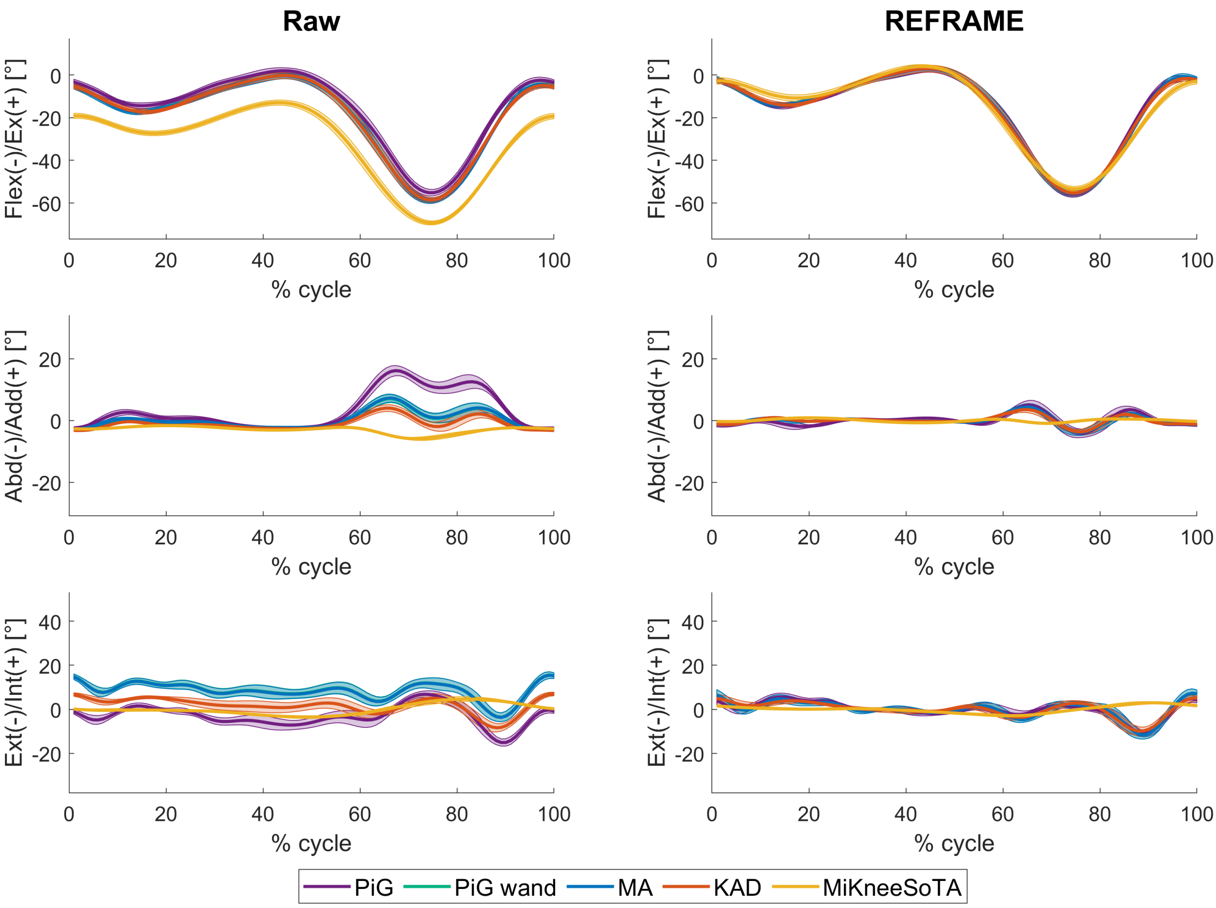

Supplementary Figure S24: Mean kinematics for all 5 markersets with standard deviation over all nine steps for knee 23 (subject 12, right). Left Column: raw kinematics, right column: REFRAMEd kinematics.

	Femur					Tibia				
markerset	PiG	PiG wand	MA	KAD	MiKneeSoTA	PiG	PiG wand	MA	KAD	MiKneeSoTA
Rx	-0.5 ± 0.3	-3.3 ± 0.5	-3.4 ± 0.4	-3.2 ± 0.1	-16.4 ± 1.1	0.0 ± 0.0	0.0 ± 0.0	0.0 ± 0.0	0.0 ± 0.0	0.0 ± 0.0
Ry	1.6 ± 2.3	-1.9 ± 2.8	-1.9 ± 2.8	-1.6 ± 2.7	7.2 ± 1.6	3.6 ± 2.1	-0.2 ± 2.8	-0.2 ± 2.8	0.5 ± 2.7	9.5 ± 1.6
Rz	-21.3 ± 1.4	-6.6 ± 1.7	-6.9 ± 1.7	-3.9 ± 1.8	0.1 ± 0.7	-16.2 ± 1.4	-15.0 ± 1.2	-15.1 ± 1.2	-5.7 ± 1.2	1.9 ± 0.5

Supplementary Table S68: Transformations of segment frames for REFRAMEd datasets in the curves above.

**Raw data**		RMS	RMSE vs.			
			PiG wand	MA	KAD	MiKneeSoTA
PiG	Flex/Ex	23.51 ± 0.63	3.45 ± 0.13	3.54 ± 0.13	3.43 ± 0.14	16.93 ± 1.22
	Abd/Add	6.85 ± 0.46	4.79 ± 0.07	4.69 ± 0.07	6.19 ± 0.09	9.33 ± 0.57
	Ext/Int	5.89 ± 0.93	11.89 ± 0.26	11.73 ± 0.27	5.85 ± 0.26	6.06 ± 0.75
PiG wand	Flex/Ex	26.38 ± 0.59	-	0.10 ± 0.00	0.46 ± 0.06	13.69 ± 1.23
	Abd/Add	2.72 ± 0.33	-	0.10 ± 0.00	1.46 ± 0.03	4.68 ± 0.52
	Ext/Int	9.52 ± 0.82	-	0.16 ± 0.01	6.81 ± 0.05	10.00 ± 0.77
MA	Flex/Ex	26.45 ± 0.59	-	-	0.48 ± 0.06	13.59 ± 1.23
	Abd/Add	2.79 ± 0.33	-	-	1.56 ± 0.03	4.78 ± 0.53
	Ext/Int	9.38 ± 0.82	-	-	6.66 ± 0.05	9.86 ± 0.76
KAD	Flex/Ex	26.29 ± 0.59	-	-	-	13.67 ± 1.20
	Abd/Add	2.26 ± 0.18	-	-	-	3.34 ± 0.46
	Ext/Int	4.25 ± 0.65	-	-	-	5.38 ± 0.55
MiKneeSoTA	Flex/Ex	37.07 ± 0.60	-	-	-	-
	Abd/Add	3.21 ± 0.19	-	-	-	-
	Ext/Int	2.55 ± 0.40	-	-	-	-

Supplementary Table S69: RMS (RMSE vs. 0) of all datasets and the RMSEs for all combinations for the datasets shown above before REFRAME.

**REFRAMEd**		RMS	RMSE vs.			
			PiG wand	MA	KAD	MiKneeSoTA
PiG	Flex/Ex	24.12 ± 0.63	0.57 ± 0.08	0.57 ± 0.08	0.67 ± 0.07	3.68 ± 0.95
	Abd/Add	2.10 ± 0.46	0.83 ± 0.06	0.84 ± 0.06	1.04 ± 0.14	2.25 ± 0.38
	Ext/Int	3.85 ± 0.93	1.22 ± 0.14	1.23 ± 0.14	1.34 ± 0.18	4.55 ± 0.41
PiG wand	Flex/Ex	24.21 ± 0.59	-	0.03 ± 0.00	0.48 ± 0.06	3.38 ± 0.98
	Abd/Add	1.68 ± 0.33	-	0.03 ± 0.00	0.37 ± 0.08	1.75 ± 0.32
	Ext/Int	4.17 ± 0.82	-	0.04 ± 0.00	1.17 ± 0.17	4.68 ± 0.50
MA	Flex/Ex	24.21 ± 0.59	-	-	0.48 ± 0.06	3.39 ± 0.98
	Abd/Add	1.69 ± 0.33	-	-	0.38 ± 0.08	1.76 ± 0.32
	Ext/Int	4.17 ± 0.82	-	-	1.18 ± 0.17	4.67 ± 0.50
KAD	Flex/Ex	24.07 ± 0.59	-	-	-	3.23 ± 0.97
	Abd/Add	1.51 ± 0.18	-	-	-	1.55 ± 0.28
	Ext/Int	3.62 ± 0.65	-	-	-	4.19 ± 0.52
MiKneeSoTA	Flex/Ex	24.13 ± 0.60	-	-	-	-
	Abd/Add	0.54 ± 0.19	-	-	-	-
	Ext/Int	1.67 ± 0.40	-	-	-	-

Supplementary Table S70: RMS (RMSE vs. 0) of all datasets and the RMSEs for all combinations for the datasets shown above after REFRAME.

3.24 Left Knee of Subject 12

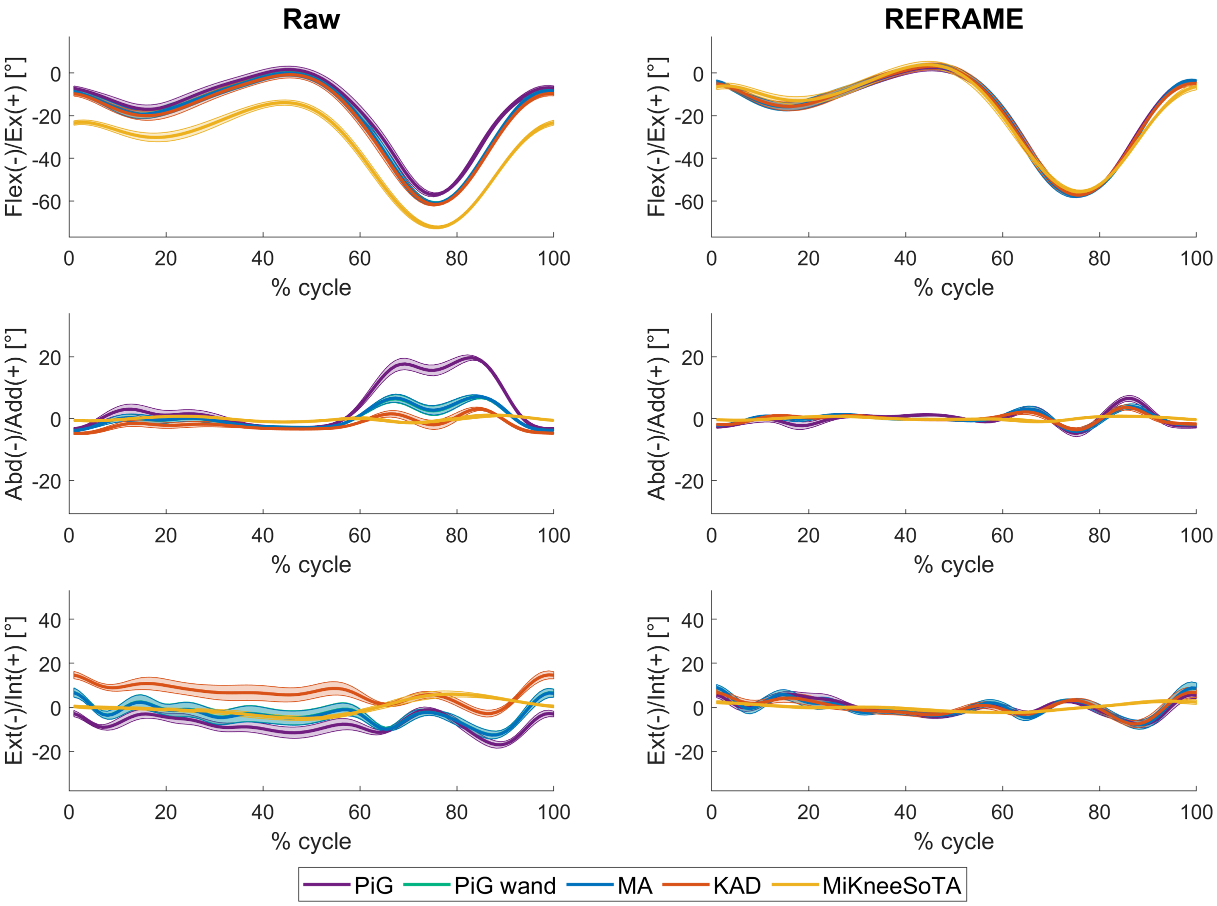

Supplementary Figure S25: Mean kinematics for all 5 markersets with standard deviation over all nine steps for knee 24 (subject 12, left). Left Column: raw kinematics, right column: REFRAMEd kinematics.

	Femur					Tibia				
markerset	PiG	PiG wand	MA	KAD	MiKneeSoTA	PiG	PiG wand	MA	KAD	MiKneeSoTA
Rx	-2.1 ± 0.4	-3.3 ± 0.2	-3.4 ± 0.2	-3.3 ± 0.7	-17.0 ± 1.2	0.0 ± 0.0	0.0 ± 0.0	0.0 ± 0.0	0.0 ± 0.0	0.0 ± 0.0
Ry	-7.6 ± 2.7	-7.9 ± 3.5	-7.9 ± 3.5	-7.8 ± 3.5	8.6 ± 1.2	-4.8 ± 2.3	-5.7 ± 3.3	-5.7 ± 3.3	-5.0 ± 3.4	9.2 ± 1.2
Rz	-23.6 ± 1.3	-6.1 ± 1.7	-6.4 ± 1.7	-1.0 ± 1.8	-4.2 ± 0.7	-15.7 ± 1.9	-4.6 ± 1.5	-4.7 ± 1.5	-9.8 ± 1.4	-1.3 ± 0.8

Supplementary Table S71: Transformations of segment frames for REFRAMEd datasets in the curves above.

**Raw data**		RMS	RMSE vs.			
			PiG wand	MA	KAD	MiKneeSoTA
PiG	Flex/Ex	24.72 ± 0.69	3.40 ± 0.13	3.48 ± 0.13	4.39 ± 0.16	17.59 ± 1.10
	Abd/Add	8.94 ± 0.55	6.26 ± 0.20	6.16 ± 0.20	8.76 ± 0.25	9.04 ± 0.61
	Ext/Int	8.94 ± 1.14	5.64 ± 0.43	5.49 ± 0.42	14.90 ± 0.45	9.40 ± 1.01
PiG wand	Flex/Ex	27.77 ± 0.73	-	0.10 ± 0.00	1.15 ± 0.04	14.55 ± 1.14
	Abd/Add	3.31 ± 0.32	-	0.11 ± 0.00	2.60 ± 0.07	3.28 ± 0.41
	Ext/Int	5.80 ± 0.90	-	0.18 ± 0.01	9.78 ± 0.02	7.35 ± 0.56
MA	Flex/Ex	27.84 ± 0.73	-	-	1.06 ± 0.04	14.45 ± 1.14
	Abd/Add	3.39 ± 0.33	-	-	2.70 ± 0.07	3.36 ± 0.42
	Ext/Int	5.89 ± 0.94	-	-	9.95 ± 0.03	7.41 ± 0.58
KAD	Flex/Ex	28.55 ± 0.74	-	-	-	13.46 ± 1.14
	Abd/Add	2.81 ± 0.18	-	-	-	2.61 ± 0.21
	Ext/Int	7.92 ± 1.38	-	-	-	9.28 ± 1.63
MiKneeSoTA	Flex/Ex	39.50 ± 0.74	-	-	-	-
	Abd/Add	0.80 ± 0.10	-	-	-	-
	Ext/Int	3.45 ± 0.37	-	-	-	-

Supplementary Table S72: RMS (RMSE vs. 0) of all datasets and the RMSEs for all combinations for the datasets shown above before REFRAME.

**REFRAMEd**		RMS	RMSE vs.			
			PiG wand	MA	KAD	MiKneeSoTA
PiG	Flex/Ex	25.70 ± 0.69	0.43 ± 0.07	0.42 ± 0.07	0.53 ± 0.06	2.95 ± 0.66
	Abd/Add	2.43 ± 0.55	1.13 ± 0.07	1.14 ± 0.07	1.31 ± 0.09	2.38 ± 0.16
	Ext/Int	3.71 ± 1.14	1.50 ± 0.29	1.52 ± 0.30	1.46 ± 0.32	3.98 ± 0.38
PiG wand	Flex/Ex	25.86 ± 0.73	-	0.03 ± 0.00	0.48 ± 0.05	2.85 ± 0.64
	Abd/Add	1.79 ± 0.32	-	0.02 ± 0.00	0.44 ± 0.06	1.76 ± 0.18
	Ext/Int	4.20 ± 0.90	-	0.05 ± 0.00	1.18 ± 0.18	4.24 ± 0.36
MA	Flex/Ex	25.87 ± 0.73	-	-	0.49 ± 0.05	2.85 ± 0.64
	Abd/Add	1.80 ± 0.33	-	-	0.45 ± 0.06	1.77 ± 0.19
	Ext/Int	4.22 ± 0.94	-	-	1.19 ± 0.17	4.24 ± 0.36
KAD	Flex/Ex	25.69 ± 0.74	-	-	-	2.64 ± 0.66
	Abd/Add	1.58 ± 0.18	-	-	-	1.53 ± 0.12
	Ext/Int	3.68 ± 1.38	-	-	-	3.74 ± 0.47
MiKneeSoTA	Flex/Ex	25.68 ± 0.74	-	-	-	-
	Abd/Add	0.55 ± 0.10	-	-	-	-
	Ext/Int	1.66 ± 0.37	-	-	-	-

Supplementary Table S73: RMS (RMSE vs. 0) of all datasets and the RMSEs for all combinations for the datasets shown above after REFRAME.

3.25 Right Knee of Subject 13

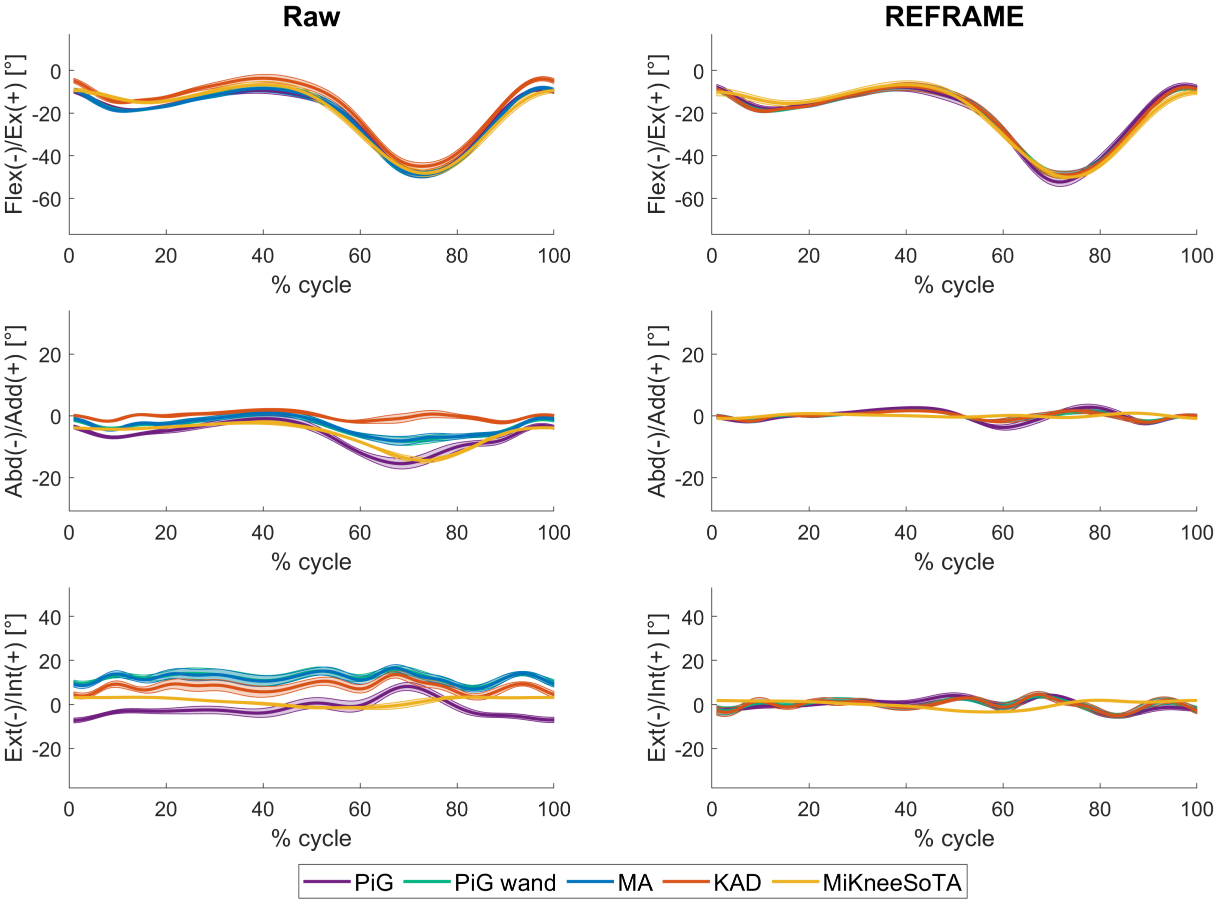

Supplementary Figure S26: Mean kinematics for all 5 markersets with standard deviation over all nine steps for knee 25 (subject 13, right). Left Column: raw kinematics, right column: REFRAMEd kinematics.

	Femur					Tibia				
markerset	PiG	PiG wand	MA	KAD	MiKneeSoTA	PiG	PiG wand	MA	KAD	MiKneeSoTA
Rx	0.9 ± 0.5	-1.3 ± 0.4	-1.4 ± 0.4	3.6 ± 0.3	-0.2 ± 0.9	0.0 ± 0.0	0.0 ± 0.0	0.0 ± 0.0	0.0 ± 0.0	0.0 ± 0.0
Ry	18.8 ± 1.4	4.7 ± 2.0	4.7 ± 2.0	3.3 ± 2.0	7.1 ± 1.6	20.9 ± 1.6	4.3 ± 2.1	4.2 ± 2.1	3.1 ± 2.1	7.5 ± 1.5
Rz	8.6 ± 2.3	10.4 ± 1.7	10.2 ± 1.7	0.3 ± 1.7	14.5 ± 1.3	16.3 ± 2.3	-1.2 ± 1.8	-1.2 ± 1.8	-6.4 ± 1.8	14.1 ± 1.5

Supplementary Table S74: Transformations of segment frames for REFRAMEd datasets in the curves above.

**Raw data**		RMS	RMSE vs.			
			PiG wand	MA	KAD	MiKneeSoTA
PiG	Flex/Ex	24.75 ± 0.66	0.59 ± 0.08	0.66 ± 0.08	4.13 ± 0.13	2.84 ± 0.56
	Abd/Add	8.03 ± 0.40	3.63 ± 0.13	3.77 ± 0.13	7.56 ± 0.23	1.96 ± 0.33
	Ext/Int	4.53 ± 0.32	14.53 ± 0.59	14.37 ± 0.59	10.10 ± 0.58	6.26 ± 0.46
PiG wand	Flex/Ex	24.97 ± 0.66	-	0.22 ± 0.01	4.08 ± 0.04	2.75 ± 0.55
	Abd/Add	4.54 ± 0.29	-	0.24 ± 0.01	4.02 ± 0.12	3.19 ± 0.28
	Ext/Int	12.57 ± 1.03	-	0.53 ± 0.10	4.54 ± 0.04	11.42 ± 1.28
MA	Flex/Ex	25.11 ± 0.66	-	-	4.21 ± 0.04	2.83 ± 0.55
	Abd/Add	4.44 ± 0.29	-	-	3.89 ± 0.12	3.34 ± 0.28
	Ext/Int	12.42 ± 1.02	-	-	4.32 ± 0.02	11.28 ± 1.27
KAD	Flex/Ex	21.83 ± 0.66	-	-	-	4.20 ± 1.09
	Abd/Add	1.29 ± 0.21	-	-	-	6.99 ± 0.29
	Ext/Int	8.35 ± 1.00	-	-	-	7.46 ± 1.19
MiKneeSoTA	Flex/Ex	24.64 ± 0.68	-	-	-	-
	Abd/Add	7.23 ± 0.26	-	-	-	-
	Ext/Int	2.42 ± 0.23	-	-	-	-

Supplementary Table S75: RMS (RMSE vs. 0) of all datasets and the RMSEs for all combinations for the datasets shown above before REFRAME.

**REFRAMEd**		RMS	RMSE vs.			
			PiG wand	MA	KAD	MiKneeSoTA
PiG	Flex/Ex	25.48 ± 0.66	1.43 ± 0.24	1.43 ± 0.24	1.45 ± 0.25	2.93 ± 0.72
	Abd/Add	1.90 ± 0.40	0.91 ± 0.11	0.91 ± 0.11	0.92 ± 0.11	1.96 ± 0.23
	Ext/Int	2.54 ± 0.32	1.45 ± 0.27	1.69 ± 0.26	1.71 ± 0.26	3.91 ± 0.60
PiG wand	Flex/Ex	25.07 ± 0.66	-	0.22 ± 0.02	0.23 ± 0.02	2.50 ± 0.73
	Abd/Add	1.15 ± 0.29	-	0.18 ± 0.01	0.17 ± 0.02	1.28 ± 0.14
	Ext/Int	2.38 ± 1.03	-	0.49 ± 0.11	0.49 ± 0.10	3.50 ± 0.44
MA	Flex/Ex	25.09 ± 0.66	-	-	0.10 ± 0.02	2.57 ± 0.72
	Abd/Add	1.23 ± 0.29	-	-	0.06 ± 0.01	1.36 ± 0.14
	Ext/Int	2.57 ± 1.02	-	-	0.09 ± 0.01	3.63 ± 0.41
KAD	Flex/Ex	25.12 ± 0.66	-	-	-	2.53 ± 0.72
	Abd/Add	1.20 ± 0.21	-	-	-	1.34 ± 0.14
	Ext/Int	2.55 ± 1.00	-	-	-	3.63 ± 0.40
MiKneeSoTA	Flex/Ex	25.47 ± 0.68	-	-	-	-
	Abd/Add	0.52 ± 0.26	-	-	-	-
	Ext/Int	1.81 ± 0.23	-	-	-	-

Supplementary Table S76: RMS (RMSE vs. 0) of all datasets and the RMSEs for all combinations for the datasets shown above after REFRAME.

3.26 Left Knee of Subject 13

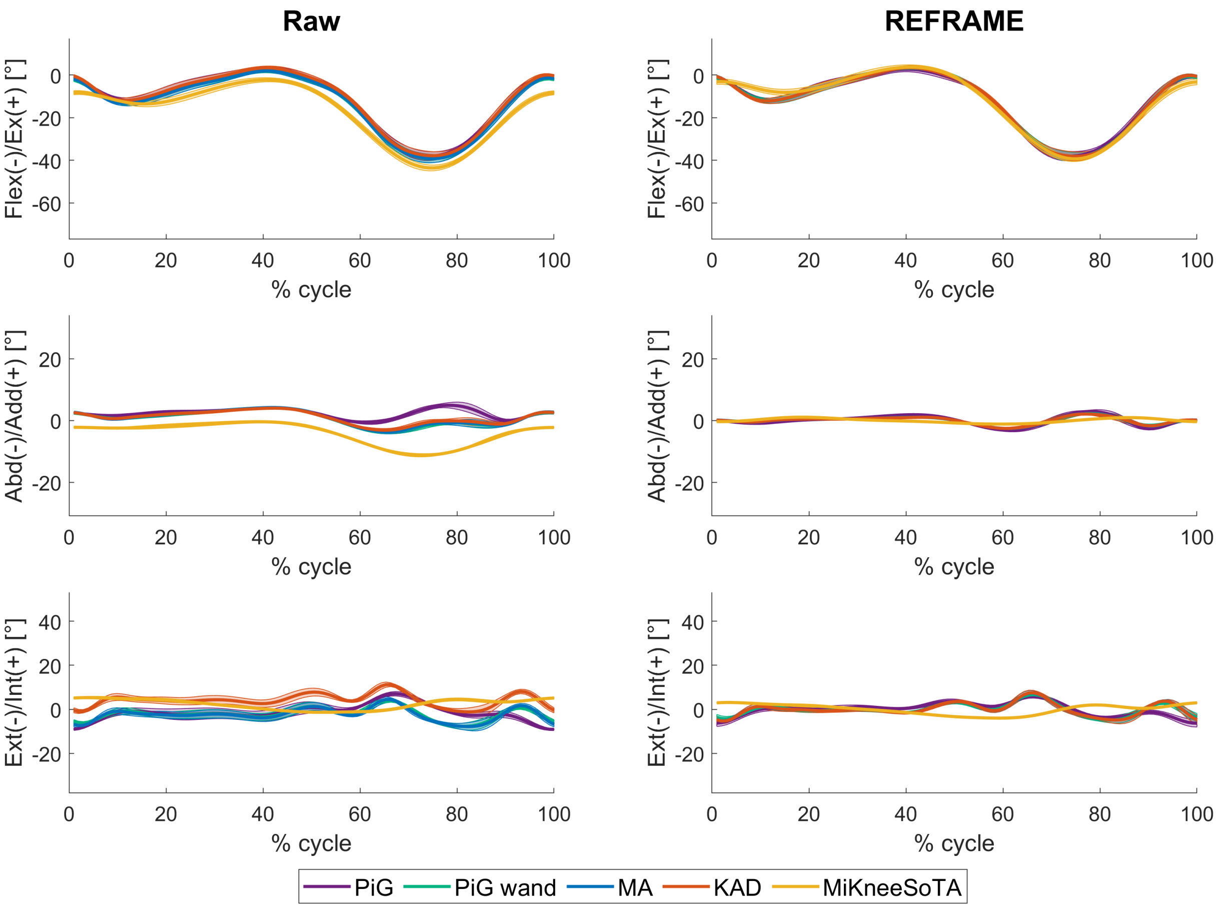

Supplementary Figure S27: Mean kinematics for all 5 markersets with standard deviation over all nine steps for knee 26 (subject 13, left). Left Column: raw kinematics, right column: REFRAMEd kinematics.

	Femur					Tibia				
markerset	PiG	PiG wand	MA	KAD	MiKneeSoTA	PiG	PiG wand	MA	KAD	MiKneeSoTA
Rx	0.3 ± 0.4	-1.2 ± 0.1	-1.3 ± 0.1	0.3 ± 0.4	-5.9 ± 0.9	0.0 ± 0.0	0.0 ± 0.0	0.0 ± 0.0	0.0 ± 0.0	0.0 ± 0.0
Ry	7.5 ± 3.9	1.0 ± 3.5	1.0 ± 3.4	0.9 ± 3.4	5.5 ± 1.2	5.2 ± 3.7	-1.6 ± 3.4	-1.6 ± 3.4	-1.6 ± 3.4	6.6 ± 1.4
Rz	-2.0 ± 0.7	9.1 ± 0.6	8.8 ± 0.6	7.6 ± 0.6	14.1 ± 0.9	1.0 ± 1.6	11.0 ± 1.2	11.0 ± 1.2	3.2 ± 1.1	12.1 ± 0.8

Supplementary Table S77: Transformations of segment frames for REFRAMEd datasets in the curves above.

**Raw data**		RMS	RMSE vs.			
			PiG wand	MA	KAD	MiKneeSoTA
PiG	Flex/Ex	17.82 ± 0.58	1.06 ± 0.15	1.21 ± 0.14	0.54 ± 0.08	5.71 ± 0.83
	Abd/Add	2.78 ± 0.14	2.50 ± 0.11	2.41 ± 0.11	2.10 ± 0.10	7.49 ± 0.22
	Ext/Int	4.10 ± 0.68	3.17 ± 0.30	3.34 ± 0.31	6.32 ± 0.45	6.63 ± 0.66
PiG wand	Flex/Ex	18.62 ± 0.57	-	0.28 ± 0.03	1.26 ± 0.01	4.82 ± 0.71
	Abd/Add	2.48 ± 0.20	-	0.17 ± 0.01	0.43 ± 0.01	5.32 ± 0.24
	Ext/Int	3.75 ± 0.37	-	0.54 ± 0.08	6.34 ± 0.01	6.52 ± 0.40
MA	Flex/Ex	18.73 ± 0.57	-	-	1.34 ± 0.00	4.78 ± 0.69
	Abd/Add	2.48 ± 0.20	-	-	0.32 ± 0.01	5.42 ± 0.24
	Ext/Int	4.04 ± 0.36	-	-	6.54 ± 0.00	6.77 ± 0.40
KAD	Flex/Ex	17.83 ± 0.57	-	-	-	5.97 ± 0.73
	Abd/Add	2.31 ± 0.19	-	-	-	5.64 ± 0.23
	Ext/Int	5.22 ± 0.57	-	-	-	5.12 ± 0.57
MiKneeSoTA	Flex/Ex	21.86 ± 0.50	-	-	-	-
	Abd/Add	5.51 ± 0.15	-	-	-	-
	Ext/Int	3.34 ± 0.28	-	-	-	-

Supplementary Table S78: RMS (RMSE vs. 0) of all datasets and the RMSEs for all combinations for the datasets shown above before REFRAME.

**REFRAMEd**		RMS	RMSE vs.			
			PiG wand	MA	KAD	MiKneeSoTA
PiG	Flex/Ex	17.88 ± 0.58	0.51 ± 0.14	0.56 ± 0.12	0.57 ± 0.12	2.36 ± 0.44
	Abd/Add	1.65 ± 0.14	0.54 ± 0.04	0.51 ± 0.04	0.54 ± 0.04	1.61 ± 0.15
	Ext/Int	3.06 ± 0.68	1.85 ± 0.23	2.07 ± 0.23	2.05 ± 0.23	5.00 ± 0.47
PiG wand	Flex/Ex	18.06 ± 0.57	-	0.25 ± 0.02	0.25 ± 0.02	2.25 ± 0.41
	Abd/Add	1.21 ± 0.20	-	0.12 ± 0.01	0.11 ± 0.01	1.12 ± 0.16
	Ext/Int	2.83 ± 0.37	-	0.50 ± 0.09	0.50 ± 0.09	4.53 ± 0.34
MA	Flex/Ex	18.09 ± 0.57	-	-	0.02 ± 0.00	2.36 ± 0.41
	Abd/Add	1.25 ± 0.20	-	-	0.05 ± 0.02	1.16 ± 0.17
	Ext/Int	3.05 ± 0.36	-	-	0.05 ± 0.01	4.67 ± 0.37
KAD	Flex/Ex	18.08 ± 0.57	-	-	-	2.36 ± 0.41
	Abd/Add	1.21 ± 0.19	-	-	-	1.13 ± 0.17
	Ext/Int	3.07 ± 0.57	-	-	-	4.70 ± 0.37
MiKneeSoTA	Flex/Ex	18.32 ± 0.50	-	-	-	-
	Abd/Add	0.71 ± 0.15	-	-	-	-
	Ext/Int	2.30 ± 0.28	-	-	-	-

Supplementary Table S79: RMS (RMSE vs. 0) of all datasets and the RMSEs for all combinations for the datasets shown above after REFRAME.

3.27 Right Knee of Subject 14

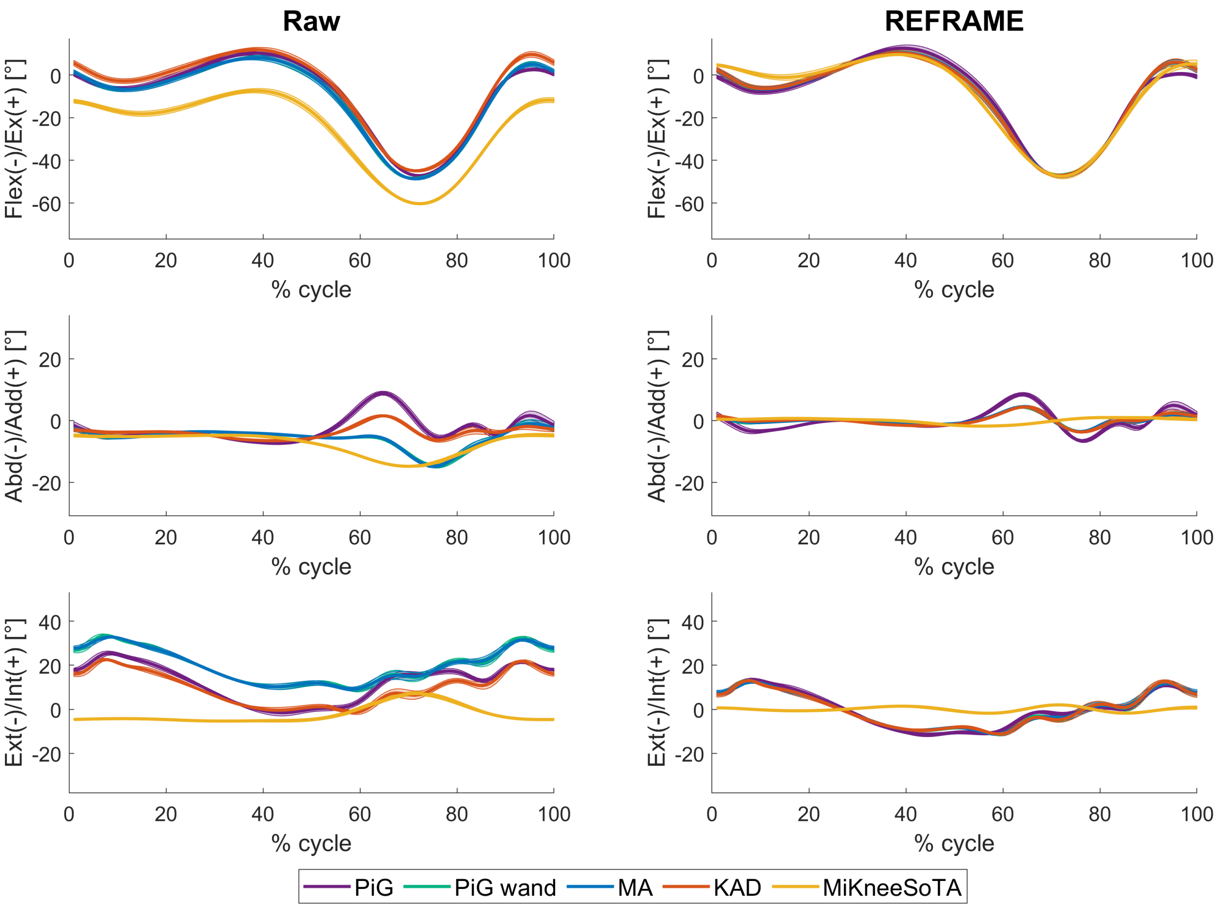

Supplementary Figure S28: Mean kinematics for all 5 markersets with standard deviation over all nine steps for knee 27 (subject 14, right). Left Column: raw kinematics, right column: REFRAMEd kinematics.

	Femur					Tibia				
markerset	PiG	PiG wand	MA	KAD	MiKneeSoTA	PiG	PiG wand	MA	KAD	MiKneeSoTA
Rx	-1.9 ± 0.4	-3.7 ± 0.8	-3.7 ± 0.8	1.6 ± 0.4	-15.4 ± 0.9	0.0 ± 0.0	0.0 ± 0.0	0.0 ± 0.0	0.0 ± 0.0	0.0 ± 0.0
Ry	5.7 ± 1.9	1.7 ± 2.2	1.7 ± 2.2	1.2 ± 2.3	15.0 ± 0.9	9.5 ± 1.9	6.4 ± 2.4	6.4 ± 2.4	5.6 ± 2.4	20.8 ± 0.9
Rz	-8.8 ± 0.6	8.9 ± 0.6	8.9 ± 0.6	-2.9 ± 0.6	5.9 ± 0.5	-19.8 ± 0.8	-11.3 ± 0.6	-11.3 ± 0.6	-12.2 ± 0.6	10.1 ± 0.5

Supplementary Table S80: Transformations of segment frames for REFRAMEd datasets in the curves above.

Raw data		RMS	RMSE vs.			
			PiG wand	MA	KAD	MiKneeSoTA
PiG	Flex/Ex	20.04 ± 0.25	2.22 ± 0.09	2.20 ± 0.09	3.06 ± 0.13	16.54 ± 1.00
	Abd/Add	4.92 ± 0.18	6.16 ± 0.18	6.14 ± 0.18	2.67 ± 0.17	8.38 ± 0.26
	Ext/Int	14.89 ± 0.38	8.55 ± 0.21	8.54 ± 0.22	3.80 ± 0.27	16.90 ± 0.40
PiG wand	Flex/Ex	21.16 ± 0.27	-	0.18 ± 0.01	3.94 ± 0.06	15.04 ± 0.97
	Abd/Add	6.54 ± 0.28	-	0.12 ± 0.01	3.99 ± 0.04	2.94 ± 0.16
	Ext/Int	21.50 ± 0.43	-	0.47 ± 0.04	10.31 ± 0.03	24.22 ± 0.46
MA	Flex/Ex	21.15 ± 0.27	-	-	3.95 ± 0.06	15.03 ± 0.97
	Abd/Add	6.51 ± 0.28	-	-	3.97 ± 0.04	2.96 ± 0.16
	Ext/Int	21.48 ± 0.43	-	-	10.32 ± 0.04	24.20 ± 0.46
KAD	Flex/Ex	19.55 ± 0.22	-	-	-	18.92 ± 0.97
	Abd/Add	4.33 ± 0.23	-	-	-	5.92 ± 0.20
	Ext/Int	12.40 ± 0.34	-	-	-	15.03 ± 0.35
MiKneeSoTA	Flex/Ex	30.92 ± 0.39	-	-	-	-
	Abd/Add	8.29 ± 0.19	-	-	-	-
	Ext/Int	4.60 ± 0.20	-	-	-	-

Supplementary Table S81: RMS (RMSE vs. 0) of all datasets and the RMSEs for all combinations for the datasets shown above before REFRAME.

REFRAMEd		RMS	RMSE vs.			
			PiG wand	MA	KAD	MiKneeSoTA
PiG	Flex/Ex	20.46 ± 0.25	2.10 ± 0.28	2.08 ± 0.27	2.23 ± 0.28	4.45 ± 0.82
	Abd/Add	3.41 ± 0.18	1.91 ± 0.20	1.92 ± 0.20	1.98 ± 0.18	3.90 ± 0.28
	Ext/Int	8.09 ± 0.38	1.46 ± 0.17	1.42 ± 0.18	1.54 ± 0.18	8.23 ± 0.36
PiG wand	Flex/Ex	20.53 ± 0.27	-	0.20 ± 0.02	0.16 ± 0.01	3.34 ± 0.54
	Abd/Add	1.71 ± 0.28	-	0.11 ± 0.01	0.27 ± 0.01	2.17 ± 0.11
	Ext/Int	7.74 ± 0.43	-	0.47 ± 0.04	0.15 ± 0.01	7.90 ± 0.31
MA	Flex/Ex	20.51 ± 0.27	-	-	0.24 ± 0.02	3.30 ± 0.55
	Abd/Add	1.71 ± 0.28	-	-	0.28 ± 0.01	2.18 ± 0.10
	Ext/Int	7.67 ± 0.43	-	-	0.47 ± 0.04	7.84 ± 0.32
KAD	Flex/Ex	20.54 ± 0.22	-	-	-	3.24 ± 0.53
	Abd/Add	1.80 ± 0.23	-	-	-	2.19 ± 0.10
	Ext/Int	7.74 ± 0.34	-	-	-	7.91 ± 0.33
MiKneeSoTA	Flex/Ex	20.92 ± 0.39	-	-	-	-
	Abd/Add	0.92 ± 0.19	-	-	-	-
	Ext/Int	1.12 ± 0.20	-	-	-	-

Supplementary Table S82: RMS (RMSE vs. 0) of all datasets and the RMSEs for all combinations for the datasets shown above after REFRAME.

3.28 Left Knee of Subject 14

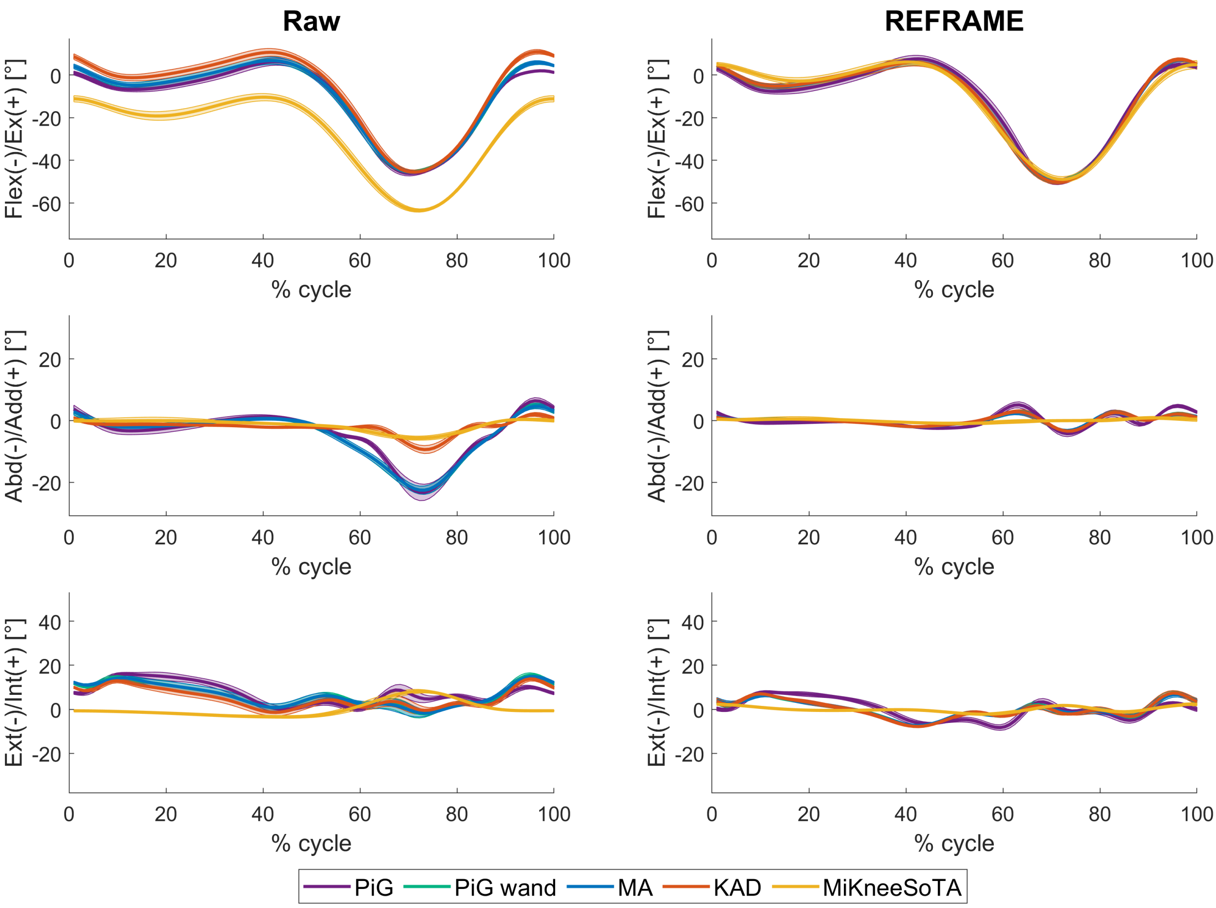

Supplementary Figure S29: Mean kinematics for all 5 markersets with standard deviation over all nine steps for knee 28 (subject 14, left). Left Column: raw kinematics, right column: REFRAMEd kinematics.

	Femur					Tibia				
markerset	PiG	PiG wand	MA	KAD	MiKneeSoTA	PiG	PiG wand	MA	KAD	MiKneeSoTA
Rx	-1.7 ± 0.4	0.5 ± 0.3	0.5 ± 0.3	4.4 ± 0.2	-15.9 ± 1.1	0.0 ± 0.0	0.0 ± 0.0	0.0 ± 0.0	0.0 ± 0.0	0.0 ± 0.0
Ry	6.4 ± 1.9	-0.8 ± 2.8	-0.8 ± 2.8	-2.5 ± 2.8	11.8 ± 0.9	6.3 ± 1.6	-0.6 ± 2.7	-0.7 ± 2.7	-1.5 ± 2.7	12.3 ± 1.0
Rz	23.3 ± 1.8	26.1 ± 1.2	26.1 ± 1.2	7.7 ± 1.2	1.7 ± 0.6	15.7 ± 1.4	17.8 ± 0.8	17.8 ± 0.8	1.5 ± 0.8	3.6 ± 0.8

Supplementary Table S83: Transformations of segment frames for REFRAMEd datasets in the curves above.

**Raw data**		RMS	RMSE vs.			
			PiG wand	MA	KAD	MiKneeSoTA
PiG	Flex/Ex	20.18 ± 0.51	1.76 ± 0.15	1.76 ± 0.15	4.84 ± 0.26	16.48 ± 0.95
	Abd/Add	8.67 ± 0.66	1.38 ± 0.21	1.39 ± 0.21	5.73 ± 0.33	6.48 ± 0.69
	Ext/Int	8.87 ± 0.73	3.87 ± 0.29	3.90 ± 0.29	3.86 ± 0.69	9.65 ± 0.84
PiG wand	Flex/Ex	19.99 ± 0.48	-	0.16 ± 0.01	3.31 ± 0.12	17.53 ± 0.98
	Abd/Add	8.93 ± 0.39	-	0.09 ± 0.01	6.04 ± 0.14	6.63 ± 0.37
	Ext/Int	8.31 ± 0.74	-	0.36 ± 0.05	1.81 ± 0.03	9.82 ± 1.00
MA	Flex/Ex	19.98 ± 0.48	-	-	3.32 ± 0.12	17.52 ± 0.98
	Abd/Add	8.90 ± 0.38	-	-	6.02 ± 0.14	6.60 ± 0.37
	Ext/Int	8.26 ± 0.74	-	-	1.85 ± 0.03	9.78 ± 0.99
KAD	Flex/Ex	19.85 ± 0.50	-	-	-	20.50 ± 1.02
	Abd/Add	3.32 ± 0.35	-	-	-	1.60 ± 0.16
	Ext/Int	6.95 ± 0.65	-	-	-	8.22 ± 0.95
MiKneeSoTA	Flex/Ex	32.81 ± 0.59	-	-	-	-
	Abd/Add	2.52 ± 0.29	-	-	-	-
	Ext/Int	3.52 ± 0.26	-	-	-	-

Supplementary Table S84: RMS (RMSE vs. 0) of all datasets and the RMSEs for all combinations for the datasets shown above before REFRAME.

**REFRAMEd**		RMS	RMSE vs.			
			PiG wand	MA	KAD	MiKneeSoTA
PiG	Flex/Ex	21.28 ± 0.51	1.84 ± 0.08	1.84 ± 0.08	1.92 ± 0.09	4.01 ± 0.82
	Abd/Add	2.29 ± 0.66	1.16 ± 0.15	1.19 ± 0.15	1.14 ± 0.14	2.23 ± 0.23
	Ext/Int	4.77 ± 0.73	2.96 ± 0.63	2.99 ± 0.62	3.03 ± 0.66	4.33 ± 0.35
PiG wand	Flex/Ex	21.70 ± 0.48	-	0.15 ± 0.01	0.17 ± 0.03	3.03 ± 0.66
	Abd/Add	1.52 ± 0.39	-	0.08 ± 0.01	0.11 ± 0.01	1.47 ± 0.14
	Ext/Int	3.95 ± 0.74	-	0.37 ± 0.05	0.26 ± 0.05	3.46 ± 0.24
MA	Flex/Ex	21.69 ± 0.48	-	-	0.24 ± 0.02	2.99 ± 0.66
	Abd/Add	1.49 ± 0.38	-	-	0.14 ± 0.01	1.45 ± 0.13
	Ext/Int	3.85 ± 0.74	-	-	0.44 ± 0.04	3.35 ± 0.26
KAD	Flex/Ex	21.81 ± 0.50	-	-	-	3.04 ± 0.63
	Abd/Add	1.55 ± 0.35	-	-	-	1.51 ± 0.13
	Ext/Int	4.09 ± 0.65	-	-	-	3.62 ± 0.28
MiKneeSoTA	Flex/Ex	21.73 ± 0.59	-	-	-	-
	Abd/Add	0.72 ± 0.29	-	-	-	-
	Ext/Int	1.30 ± 0.26	-	-	-	-

Supplementary Table S85: RMS (RMSE vs. 0) of all datasets and the RMSEs for all combinations for the datasets shown above after REFRAME.

3.29 Right Knee of Subject 15

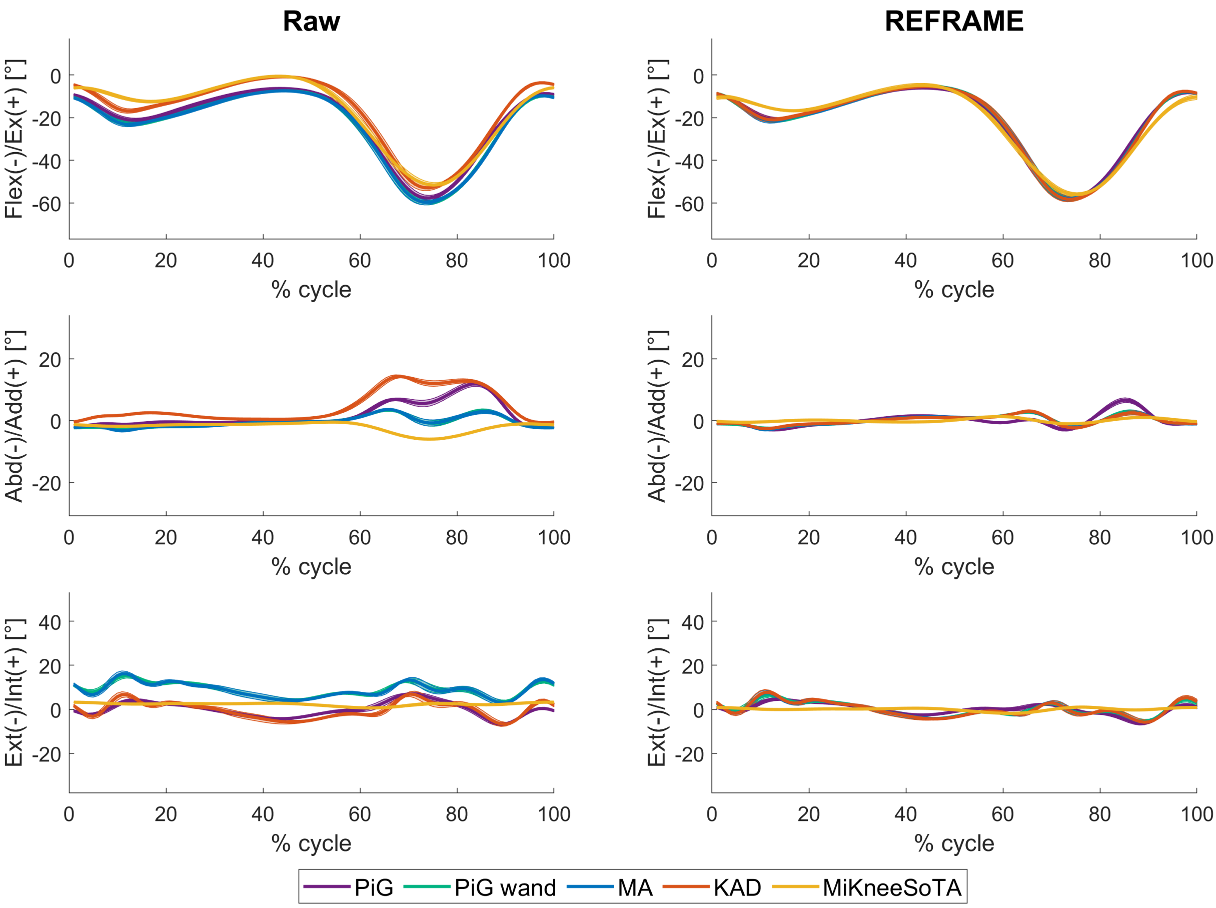

Supplementary Figure S30: Mean kinematics for all 5 markersets with standard deviation over all nine steps for knee 29 (subject 15, right). Left Column: raw kinematics, right column: REFRAMEd kinematics.

	Femur					Tibia				
markerset	PiG	PiG wand	MA	KAD	MiKneeSoTA	PiG	PiG wand	MA	KAD	MiKneeSoTA
Rx	-0.5 ± 0.1	-2.3 ± 0.2	-2.3 ± 0.2	3.9 ± 0.1	4.1 ± 1.1	0.0 ± 0.0	0.0 ± 0.0	0.0 ± 0.0	0.0 ± 0.0	0.0 ± 0.0
Ry	1.1 ± 1.2	0.6 ± 1.4	0.6 ± 1.5	-2.8 ± 1.5	2.0 ± 0.8	4.4 ± 1.0	2.5 ± 1.4	2.5 ± 1.4	-0.6 ± 1.4	2.2 ± 0.8
Rz	-14.5 ± 0.8	-4.1 ± 0.6	-4.1 ± 0.6	-18.1 ± 0.6	4.7 ± 0.6	-12.5 ± 1.1	-12.3 ± 0.7	-12.3 ± 0.7	-16.9 ± 0.7	2.6 ± 0.5

Supplementary Table S86: Transformations of segment frames for REFRAMEd datasets in the curves above.

**Raw data**		RMS	RMSE vs.			
			PiG wand	MA	KAD	MiKneeSoTA
PiG	Flex/Ex	27.12 ± 0.72	2.20 ± 0.05	2.26 ± 0.06	4.99 ± 0.03	5.37 ± 0.93
	Abd/Add	4.37 ± 0.26	3.57 ± 0.12	3.56 ± 0.12	3.31 ± 0.13	6.53 ± 0.32
	Ext/Int	3.41 ± 0.30	9.13 ± 0.15	9.13 ± 0.15	1.77 ± 0.11	4.30 ± 0.30
PiG wand	Flex/Ex	29.01 ± 0.70	-	0.23 ± 0.02	6.83 ± 0.03	6.92 ± 0.98
	Abd/Add	1.88 ± 0.09	-	0.16 ± 0.01	6.23 ± 0.15	3.23 ± 0.23
	Ext/Int	9.41 ± 0.20	-	0.50 ± 0.04	9.23 ± 0.03	7.28 ± 0.22
MA	Flex/Ex	29.05 ± 0.70	-	-	6.85 ± 0.03	6.98 ± 0.98
	Abd/Add	1.88 ± 0.10	-	-	6.21 ± 0.15	3.25 ± 0.23
	Ext/Int	9.44 ± 0.21	-	-	9.17 ± 0.04	7.33 ± 0.23
KAD	Flex/Ex	23.50 ± 0.71	-	-	-	3.45 ± 0.37
	Abd/Add	6.68 ± 0.21	-	-	-	9.14 ± 0.23
	Ext/Int	3.81 ± 0.42	-	-	-	4.61 ± 0.42
MiKneeSoTA	Flex/Ex	24.02 ± 0.68	-	-	-	-
	Abd/Add	2.61 ± 0.11	-	-	-	-
	Ext/Int	2.42 ± 0.18	-	-	-	-

Supplementary Table S87: RMS (RMSE vs. 0) of all datasets and the RMSEs for all combinations for the datasets shown above before REFRAME.

**REFRAMEd**		RMS	RMSE vs.			
			PiG wand	MA	KAD	MiKneeSoTA
PiG	Flex/Ex	27.33 ± 0.72	0.76 ± 0.04	0.84 ± 0.04	0.91 ± 0.04	2.98 ± 0.28
	Abd/Add	2.18 ± 0.26	1.46 ± 0.07	1.49 ± 0.07	1.53 ± 0.07	2.04 ± 0.21
	Ext/Int	2.57 ± 0.30	1.39 ± 0.09	1.63 ± 0.10	1.67 ± 0.10	2.73 ± 0.28
PiG wand	Flex/Ex	27.48 ± 0.70	-	0.24 ± 0.02	0.34 ± 0.02	2.97 ± 0.25
	Abd/Add	1.52 ± 0.09	-	0.16 ± 0.02	0.17 ± 0.01	1.29 ± 0.12
	Ext/Int	3.10 ± 0.20	-	0.50 ± 0.03	0.53 ± 0.04	3.07 ± 0.33
MA	Flex/Ex	27.50 ± 0.70	-	-	0.24 ± 0.01	3.05 ± 0.25
	Abd/Add	1.53 ± 0.10	-	-	0.15 ± 0.01	1.31 ± 0.13
	Ext/Int	3.29 ± 0.21	-	-	0.10 ± 0.01	3.27 ± 0.33
KAD	Flex/Ex	27.60 ± 0.71	-	-	-	2.99 ± 0.27
	Abd/Add	1.47 ± 0.21	-	-	-	1.23 ± 0.12
	Ext/Int	3.35 ± 0.42	-	-	-	3.33 ± 0.34
MiKneeSoTA	Flex/Ex	27.38 ± 0.68	-	-	-	-
	Abd/Add	0.61 ± 0.11	-	-	-	-
	Ext/Int	0.73 ± 0.18	-	-	-	-

Supplementary Table S88: RMS (RMSE vs. 0) of all datasets and the RMSEs for all combinations for the datasets shown above after REFRAME.

3.30 Left Knee of Subject 15

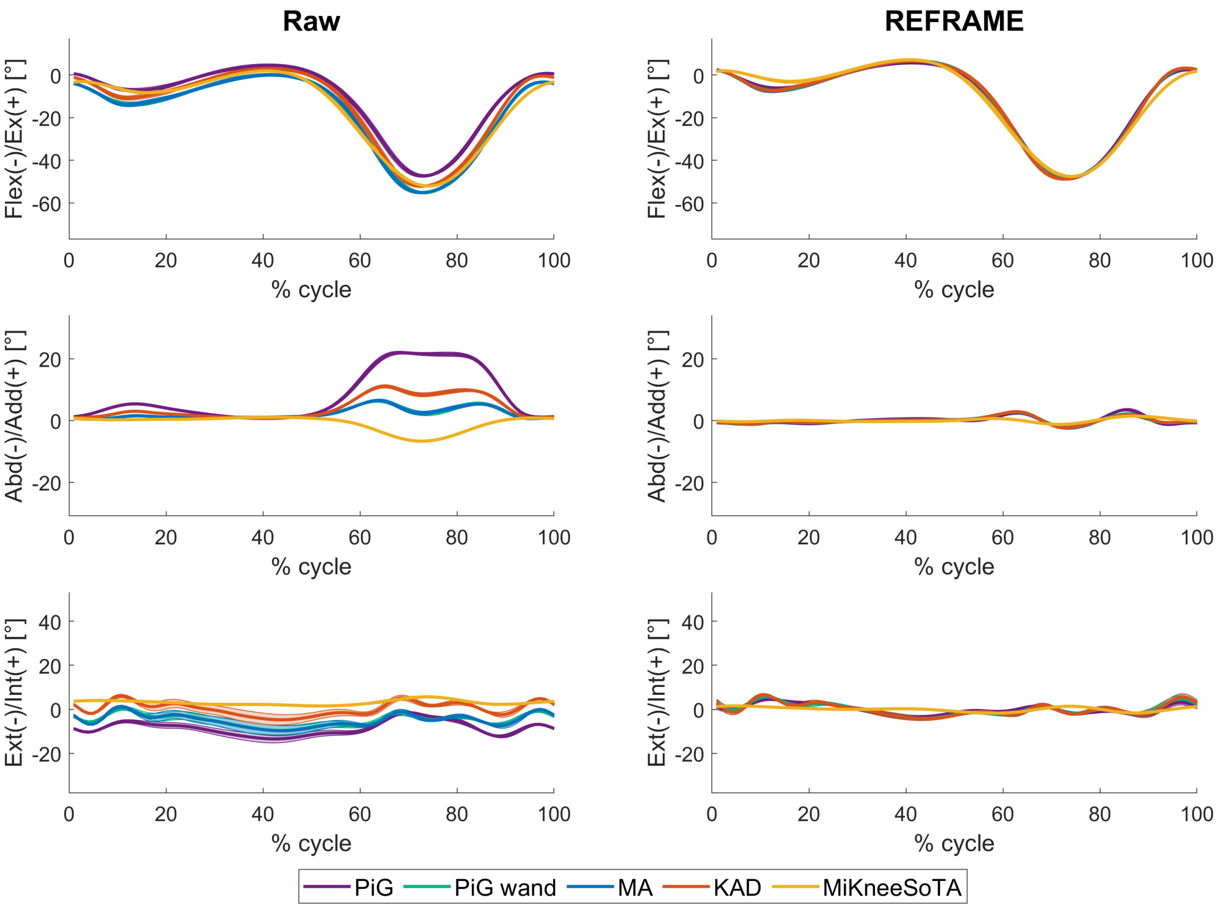

Supplementary Figure S31: Mean kinematics for all 5 markersets with standard deviation over all nine steps for knee 30 (subject 15, left). Left Column: raw kinematics, right column: REFRAMEd kinematics.

	Femur					Tibia				
markerset	PiG	PiG wand	MA	KAD	MiKneeSoTA	PiG	PiG wand	MA	KAD	MiKneeSoTA
Rx	-1.5 ± 0.2	-6.4 ± 0.2	-6.4 ± 0.2	-3.5 ± 0.1	-5.4 ± 0.6	0.0 ± 0.0	0.0 ± 0.0	0.0 ± 0.0	0.0 ± 0.0	0.0 ± 0.0
Ry	-0.5 ± 1.6	1.5 ± 2.0	1.5 ± 2.0	0.7 ± 2.0	4.9 ± 0.8	-3.4 ± 1.5	0.1 ± 2.0	0.1 ± 2.0	-1.2 ± 2.0	4.3 ± 0.9
Rz	-28.1 ± 0.8	-4.8 ± 1.0	-4.9 ± 1.0	-11.9 ± 1.1	6.2 ± 0.4	-18.1 ± 0.6	0.7 ± 0.7	0.6 ± 0.7	-11.3 ± 0.7	3.6 ± 0.4

Supplementary Table S89: Transformations of segment frames for REFRAMEd datasets in the curves above.

**Raw data**		RMS	RMSE vs.			
			PiG wand	MA	KAD	MiKneeSoTA
PiG	Flex/Ex	19.86 ± 0.33	6.71 ± 0.06	6.75 ± 0.06	3.80 ± 0.06	6.07 ± 0.48
	Abd/Add	11.22 ± 0.20	8.72 ± 0.10	8.70 ± 0.10	5.93 ± 0.07	13.45 ± 0.27
	Ext/Int	9.15 ± 0.69	4.12 ± 0.29	4.15 ± 0.29	9.00 ± 0.31	11.86 ± 0.70
PiG wand	Flex/Ex	25.22 ± 0.33	-	0.21 ± 0.01	3.10 ± 0.01	3.10 ± 0.47
	Abd/Add	2.82 ± 0.18	-	0.12 ± 0.01	2.79 ± 0.03	4.81 ± 0.30
	Ext/Int	5.68 ± 0.86	-	0.45 ± 0.03	5.27 ± 0.02	8.33 ± 0.88
MA	Flex/Ex	25.26 ± 0.33	-	-	3.11 ± 0.01	3.17 ± 0.46
	Abd/Add	2.83 ± 0.18	-	-	2.77 ± 0.03	4.83 ± 0.30
	Ext/Int	5.79 ± 0.86	-	-	5.28 ± 0.02	8.41 ± 0.88
KAD	Flex/Ex	23.08 ± 0.31	-	-	-	3.40 ± 0.29
	Abd/Add	5.40 ± 0.20	-	-	-	7.55 ± 0.29
	Ext/Int	3.28 ± 0.45	-	-	-	3.82 ± 0.67
MiKneeSoTA	Flex/Ex	24.18 ± 0.27	-	-	-	-
	Abd/Add	2.53 ± 0.11	-	-	-	-
	Ext/Int	3.28 ± 0.16	-	-	-	-

Supplementary Table S90: RMS (RMSE vs. 0) of all datasets and the RMSEs for all combinations for the datasets shown above before REFRAME.

**REFRAMEd**		RMS	RMSE vs.			
			PiG wand	MA	KAD	MiKneeSoTA
PiG	Flex/Ex	21.05 ± 0.33	0.56 ± 0.06	0.65 ± 0.05	0.65 ± 0.04	2.45 ± 0.33
	Abd/Add	1.32 ± 0.20	0.46 ± 0.06	0.50 ± 0.05	0.50 ± 0.05	1.07 ± 0.08
	Ext/Int	2.26 ± 0.69	0.88 ± 0.13	1.10 ± 0.13	1.09 ± 0.13	2.09 ± 0.29
PiG wand	Flex/Ex	21.23 ± 0.33	-	0.21 ± 0.02	0.23 ± 0.01	2.60 ± 0.31
	Abd/Add	1.14 ± 0.18	-	0.12 ± 0.02	0.12 ± 0.01	0.93 ± 0.07
	Ext/Int	2.67 ± 0.86	-	0.45 ± 0.03	0.46 ± 0.03	2.39 ± 0.34
MA	Flex/Ex	21.25 ± 0.33	-	-	0.11 ± 0.01	2.68 ± 0.31
	Abd/Add	1.12 ± 0.18	-	-	0.05 ± 0.02	0.92 ± 0.07
	Ext/Int	2.83 ± 0.86	-	-	0.07 ± 0.01	2.57 ± 0.29
KAD	Flex/Ex	21.31 ± 0.31	-	-	-	2.66 ± 0.29
	Abd/Add	1.13 ± 0.20	-	-	-	0.92 ± 0.07
	Ext/Int	2.85 ± 0.45	-	-	-	2.60 ± 0.29
MiKneeSoTA	Flex/Ex	21.24 ± 0.27	-	-	-	-
	Abd/Add	0.61 ± 0.11	-	-	-	-
	Ext/Int	1.01 ± 0.16	-	-	-	-

Supplementary Table S91: RMS (RMSE vs. 0) of all datasets and the RMSEs for all combinations for the datasets shown above after REFRAME.
